# Cam*K*-DB: A *k*-mer MinHash fingerprint database for reference-free genotyping of *Camellia* accessions

**DOI:** 10.1093/gigascience/giag088

**Published:** 2026-08-21

**Authors:** Feiquan Wang, Noor-ul Áin, Fang Wang, Shengcheng Zhang, Weilong Kong, Yutao Shi, Hua Feng, Bo Zhang, Xingtan Zhang

**Affiliations:** College of Tea and Food Sciences, Collaborative Innovation Center of Chinese Oolong Tea Industry, Wuyi University, Tea Engineering Research Center of Fujian Higher Education, Wuyishan Fujian 354300, China; State Key Laboratory of Tropical Crop Breeding, Shenzhen Branch, Guangdong Laboratory for Lingnan Modern Agriculture, Genome Analysis Laboratory of the Ministry of Agriculture and Rural Affairs, Agricultural Genomics Institute at Shenzhen, Chinese Academy of Agricultural Sciences, Shenzhen 518120, China; State Key Laboratory of Tropical Crop Breeding, Shenzhen Branch, Guangdong Laboratory for Lingnan Modern Agriculture, Genome Analysis Laboratory of the Ministry of Agriculture and Rural Affairs, Agricultural Genomics Institute at Shenzhen, Chinese Academy of Agricultural Sciences, Shenzhen 518120, China; State Key Laboratory of Tropical Crop Breeding, Shenzhen Branch, Guangdong Laboratory for Lingnan Modern Agriculture, Genome Analysis Laboratory of the Ministry of Agriculture and Rural Affairs, Agricultural Genomics Institute at Shenzhen, Chinese Academy of Agricultural Sciences, Shenzhen 518120, China; State Key Laboratory of Tropical Crop Breeding, Shenzhen Branch, Guangdong Laboratory for Lingnan Modern Agriculture, Genome Analysis Laboratory of the Ministry of Agriculture and Rural Affairs, Agricultural Genomics Institute at Shenzhen, Chinese Academy of Agricultural Sciences, Shenzhen 518120, China; College of Tea and Food Sciences, Collaborative Innovation Center of Chinese Oolong Tea Industry, Wuyi University, Tea Engineering Research Center of Fujian Higher Education, Wuyishan Fujian 354300, China; College of Tea and Food Sciences, Collaborative Innovation Center of Chinese Oolong Tea Industry, Wuyi University, Tea Engineering Research Center of Fujian Higher Education, Wuyishan Fujian 354300, China; College of Tea and Food Sciences, Collaborative Innovation Center of Chinese Oolong Tea Industry, Wuyi University, Tea Engineering Research Center of Fujian Higher Education, Wuyishan Fujian 354300, China; State Key Laboratory of Tropical Crop Breeding, Shenzhen Branch, Guangdong Laboratory for Lingnan Modern Agriculture, Genome Analysis Laboratory of the Ministry of Agriculture and Rural Affairs, Agricultural Genomics Institute at Shenzhen, Chinese Academy of Agricultural Sciences, Shenzhen 518120, China

**Keywords:** MinHash, *k*-mer, *Camellia sinensis*, alignment-free genomics, Jaccard similarity, germplasm identification, SQLite database

## Abstract

Tea (*Camellia sinensis* L.), a major global economic crop in Asia, poses challenges for genetic identification because its highly heterozygous, repetitive genome reduces the efficacy of conventional single-nucleotide polymorphism (SNP) and microsatellite markers, and interspecific hybridization further complicates the situation. To address these issues, CamK-DB was developed as a reference-free *Camellia* fingerprinting database built on MIKE MinHash sketches. We curated 418 candidate resequencing datasets, and built a database using standardized 5× genome-coverage fingerprints. Each accession is stored as a MIKE. jac fingerprint generated with *k* = 21 and recommended sketch/pre_cnt = 2000. Cam*K*-DB provides a command-line interface for data management and a custom C++ query engine that computes top-10 matches using Jaccard similarity, complemented by a QT-based graphical interface for interactive analysis. This resource offers a robust and scalable framework for precise and routine germplasm identification, genomic phylogenetic inference, and strategic breeding program design. Cam*K*-DB (database and code) is publicly available at https://github.com/sc-zhang/CamK-DB. Cam*K*-DB binaries are provided for Windows 10/11 and Linux (x86_64, glibc ≥ 2.27).

## Background

Tea plants (*Camellia sinensis*), famous for their outstanding foliage, are among the world’s most cherished non-alcoholic beverages. Beyond its scope as a beverage crop, the tea industry extends to different sectors, such as finished products, tea ware, and global expansions. Technically, *C. sinensis* (L.) is the principal cultivated species for tea production, sectioned as *C. sinensis* var. *sinensis* (small-leaf tea) and *C. sinensis* var. *assamica* (large-leaf tea). Additionally, several wild relatives, including *C. taliensis, C. irrawadiensis, C. tachangensis*, and *C. grandibracteata*, account for the genetic diversity in China. Tea leaves face many challenges in phenotypic characterization due to their dense foliage, architecture, and phenotypic similarity. Additionally, tea plants exhibit complex genomic features, including high heterozygosity (>2%), frequent interspecific hybridization, extensive vegetative propagation, and a genome dominated by repetitive sequences such as long terminal repeat (LTR) retrotransposons (~70% of the genome) [1]. These characteristics complicate traditional genetic analysis and cultivar identification. Thus, mislabeling and fraudulent substitution of tea varieties undermine consumer trust and economic stability, necessitating a robust anti-counterfeiting measure for the rapid and accurate identification of tea varieties.

Current methods for tea variety identification, such as morphological analysis and chemical profiling, are often time-consuming and lack the precision to distinguish closely related species and varieties [2,3]. Conventional methods of DNA fingerprinting relied on molecular markers (e.g. SSRs/microsatellites, amplified fragment length polymorphism), allowing cultivar identification and population genetics, but showed limited genome-wide resolution. Reduced representation approaches such as RAD-seq and genotyping-by-sequencing (GBS) based on short reads matured into alignment-based pipelines that infer SNPs against a reference genome [4,5]. Molecular markers, such as simple sequence repeats (SSRs) and single nucleotide polymorphisms (SNPs), are accurate and efficient but entail high genotyping costs and complexity [2,3]. These methods benefited from multiple-sequence-alignment tools (e.g. BLAST, ClustalW) that remain foundational and widely used [6]. Recently, *k*-mer-based analytical approaches have been extensively utilized in computational biology; however, the determination of an optimal *k*-mer length remains contingent upon heuristic optimization methodologies. Several hashing-based techniques, such as MinHash, Bloom filter, and Count-Min Sketch [7], have been developed or adopted to compute k-mer-based similarity methods efficiently. MinHash efficiently estimates genetic similarity by representing genomes as compact sketches, thereby significantly reducing the computational demands, rather than performing membership testing or frequency estimation. Although Bloom filters are highly memory-efficient and useful for filtering or indexing large *k*-mer collections, they do not directly provide an unbiased estimate of set similarity; false positives may inflate the apparent overlap between two samples unless additional correction or processing is applied. Similarly, Count-Min Sketch is primarily designed for approximate frequency estimation in streaming data and is therefore more appropriate for abundance-related queries than for direct set similarity comparisons. Inspired by these advancements, we previously developed the MinHash-based *k*-mer algorithm MIKE, tailored to process extensive genomic data, generate compact genetic signatures, and enable rapid similarity calculations [8]. Accordingly, CamK-DB adopts the MIKE MinHash framework for reference-free generation and comparison of compact genomic fingerprints. Tea genomics has recently been endowed with two chromosome-scale assemblies of *C. lanceoleosa* and wild oil-*Camellia* and a haplotype-resolved tetraploid *C. oleifera* genome, providing valuable insights into genome evolution, population structure, self-incompatibility, and agronomic traits [9–12]. Owing to the availability of large-scale genomic datasets, there is a dire need for a centralized and standardized repository that can facilitate the identification of different accessions and varieties irrespective of phenotypic and chemical methods. Many recent studies have contributed to the compilation of valuable datasets in various aspects of tea, such as an atlas of genomic variation, metabolites of tea cultivars, or aspects of tea plant biology [13,14]. Despite these recent advances, a publicly available *k-*mer fingerprint database for identifying tea accessions is still lacking, and Cam*K*-DB has been developed to address this gap. This alignment-free approach compares samples directly via their *k*-mer content, avoiding read mapping and multiple alignment. MinHash sketches transform large datasets into compact “signatures”, enabling rapid, accurate similarity queries with small storage footprints. As it helps in (i) reference-free operation, (ii) good performance at low-to-moderate coverage, (iii) fast search across many accessions, and (iv) easy sharing/indexing of compact sketches. Benefiting from this, we created a *k*-mer-based, low-cost, user-friendly database for germplasm identification. The base dataset is a spatially diverse resource of 418 accessions of different species of *Camellia*. Built upon QT architecture and SQLite, the database enables rapid identification of tea tree varieties, offering unprecedented accuracy and efficiency in cultivar authentication, hybrid detection, and evolutionary research.

### Theory behind database construction

Database construction was based on two key elements.

1. DNA Markers (*k*-mers): The foundation of the database lies in the genetic information obtained from 418 accessions of tea resequencing data, followed by using an alignment-free and more accurate approach, i.e. *k*-mer similarity for building a database. These fingerprints were generated using *k* = 21 based on our previously developed method MIKE, which employed rigorous evaluation with 40× sequence coverage using various *k* = 17, 19, 21, 25, and 27, and *k* = 21 provided the most stable balance between similarity-estimation accuracy and robustness. Larger *k* values showed increasing deviation from the expected values, likely because longer *k*-mers are more sensitive to sequencing errors and nucleotide variation. This trade-off is especially relevant for *Camellia* genomes, which are large, highly heterozygous, and repeat-rich. Shorter *k-*mers may increase chance matches in repetitive regions, whereas longer *k-*mers may reduce sensitivity because single-base differences disrupt the entire *k-*mer. Therefore, *k* = 21 was retained as the MIKE-supported foundational setting for standardized CamK-DB fingerprint generation. In this study, a *k*-mer refers to a contiguous DNA sequence of length *k* extracted from sequencing reads named as MIKE-derived unique fingerprints. Thus, Cam*K-*DB compares compact, standardized MIKE fingerprints. Briefly, after quality filtering and 5× coverage standardization, reads were decomposed into 21-mers. Identical 21-mers were deduplicated, and each 21-mer was divided into a 10-base prefix and an 11-base suffix. *K-*mers sharing the same 10-base prefix were grouped together, giving a fixed theoretical prefix space of 4^10^= 1,048,576 possible groups. For each prefix group, MIKE records a minimized hash-derived value from the corresponding suffixes. The resulting “.jac” fingerprint is therefore a compact prefix-MinHash representation of the sample rather than a table of MIKE-derived fingerprint elements. Because the fingerprint is represented in a standardized prefix feature space, the number of possible retained fingerprint elements is approximately fixed across samples, whereas sequencing-depth differences mainly affect which *k*-mers are recovered before minimization and therefore can influence similarity scores. To reduce this technical bias, all retained database accessions were standardized to 5× genome coverage before MIKE fingerprint generation. Coverage-matching analysis showed that similarity was highest when query and database fingerprints were generated at the same coverage Fig. 2A and B.
2. Jaccard Similarity (MinHash Signatures): MIKE harnessed the MinHash algorithm to create a compact and exclusive MinHash signature for each biological sample of tea. These MinHash fingerprints were processed to calculate the Jaccard coefficient, which is the ratio of the count of shared to unique *k*-mers between two samples. MIKE used this approximate Jaccard coefficient to calculate the evolutionary distance (d) between samples using a formula derived from the Poisson distribution, assuming single-base substitutions.

The Jaccard similarity is calculated with the formula below:


\begin{eqnarray*}
\textit{Jacard}\ \textit{Similarity} = \frac{{\left| {{S}_1 \cap {S}_2} \right|}}{{\left| {{S}_1 \cup {S}_2} \right|}}
\end{eqnarray*}

**Figure 1:**
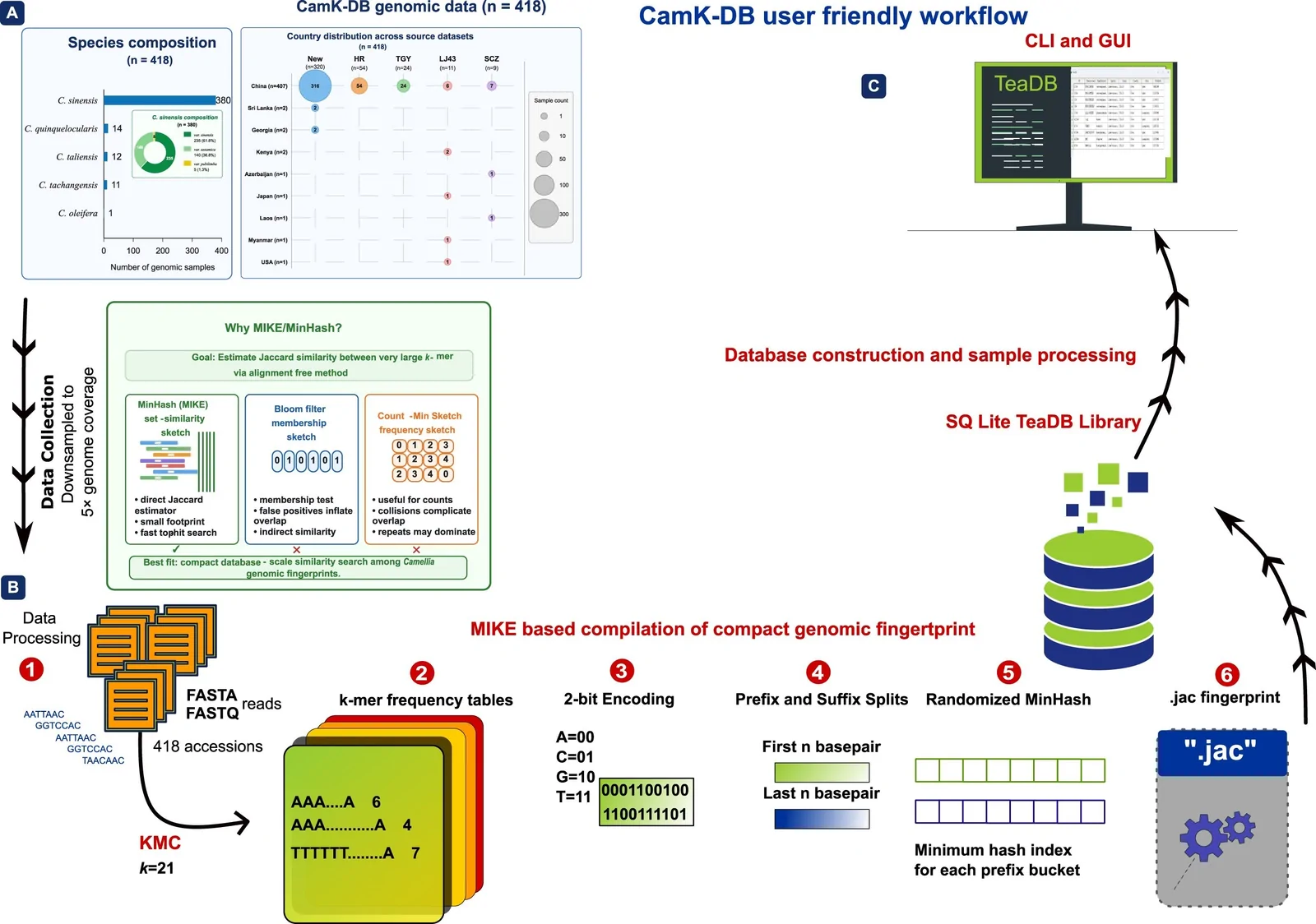
MIKE-based fingerprint generation and Jaccard similarity calculation. Quality-filtered reads are downsampled to 5× genome coverage and decomposed into 21-mers. Identical 21-mers are deduplicated, and each 21-mer is divided into a 10-bp prefix and an 11-bp suffix. Similar prefix sharing *k*-mers are grouped, and MIKE records a minimized hash-derived value from the corresponding suffix group. The resulting (prefix, MinHash [suffix]) elements are stored as “.jac” fingerprints and compared using Jaccard similarity. Query samples are processed using the same *k* = 21 and sketch/pre_cnt = 2000 settings before comparison with TEA.db.

For two samples, S₁ and S₂ represent the sets of MIKE-derived fingerprint elements generated from the two read datasets. A shared element is counted only when both the prefix and the MinHash (suffix) value match exactly between the two samples. Mismatches are not allowed. Where S_1_ denotes the *k*-mers set from sample 1, while S_2_ denotes the *k*-mers set from sample 2. The MIKE-derived fingerprint construction and Jaccard comparison are summarized in Fig. 1.

### Processing reads in MIKE

**Figure 2:**
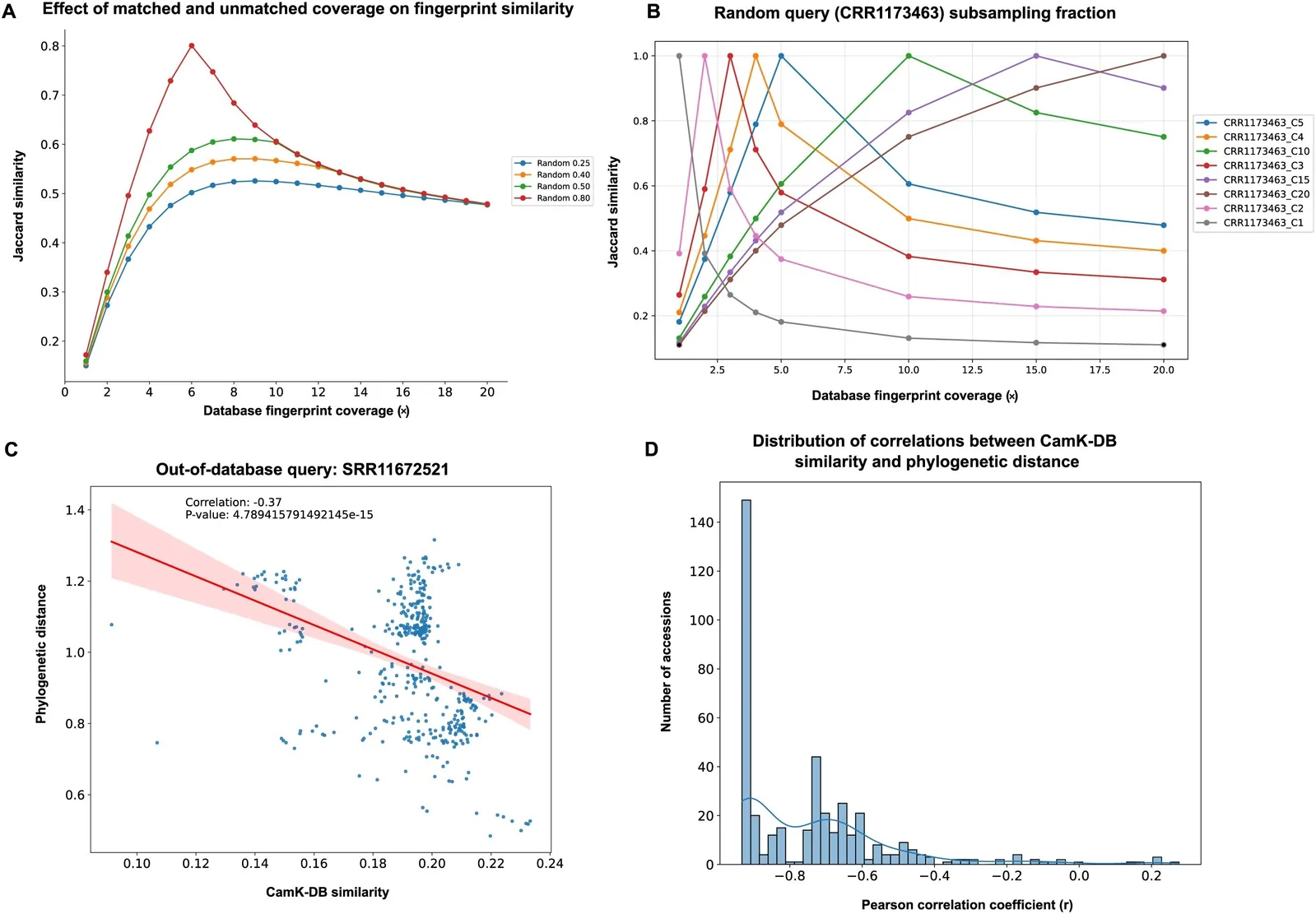
Validation of coverage standardization and biological relevance of CamK-DB similarity. (A) Effect of matched and unmatched sequencing coverage on MIKE fingerprint similarity for accession CRR1173463. (B) Random-subsampling analysis showing the effect of query subsampling level and database fingerprint coverage on similarity. (C) Relationship between Cam*K*-DB similarity and phylogenetic distance for the out-of-database accession SRR11672521. Phylogenetic distances were obtained from the published Camellia phylogenetic tree reported by Kong et al. [12]. (D) Distribution of Pearson correlation coefficients between Cam*K*-DB similarity and published phylogenetic distances. Negative values indicate that higher fingerprint similarity generally corresponds to shorter phylogenetic distance.

MIKE was developed to estimate sequence similarity based on *k*-mer frequency. We employed MIKE to enumerate all possible *k*-mers within a 2000-sketch size and then calculated the frequency of each *k*-mer. MinHash fingerprints were then derived from the *k*-mer representations of the lowest hash values. This signature fingerprint serves as a heuristic representation of a sequence, capturing its essential features. The resulting “.jac” files contained integer-count pairs encoding MinHash signatures, specific to each sample’s signature, comprising up to 1,048,576 hash buckets to capture both the presence and frequency of hashed genomic elements (Fig. 2).

### Sketch-size optimization

To evaluate the effect of sketch size on similarity estimation, we tested sketch/pre_cnt values of 100, 500, 1,000, 2000, 5,000, 8,000, and 10,000. For each setting, similarity estimates from the sketched MIKE fingerprint were compared with the full fingerprint comparison, and root mean square error (RMSE) was calculated across samples. Small sketch sizes produced higher RMSE, whereas increasing the sketch size reduced the error. The median RMSE became low and stable around sketch size 2000, while larger values provided little additional improvement. Runtime analysis showed that moderate sketch sizes had similar computational costs, whereas very large sketch sizes substantially increased query time. Therefore, sketch/pre_cnt = 2000 was selected as the recommended setting for the standardized Cam*K-*DB release Supplementary Fig. S2A and B.

### Construction of the database, GUI, and basic features

Following data collection, the database schema was constructed using an in-house Python script (create_db.py), which processed two primary inputs: a tab-delimited metadata file containing sample identifiers (ID), nomenclature (Chinese and English names), taxonomic classification (species and group), geographic provenance (country and region), and a directory of “.jac” files, each corresponding to a unique sample ID. The script generated an SQLite database file (Cam*K-*DB) designed to facilitate efficient fingerprint data retrieval through an SQLite-compatible graphical user interface (GUI). The schema featured a central tea_info table for storing dataset metadata, alongside individual tables for each sample organized by ID, which housed MinHash signatures and precomputed similarity strings. This architecture minimized reliance on cross-table joins during query filtering, thereby enhancing the computational efficiency for per-sample analytical operations while maintaining structural clarity and scalability for downstream applications.

### Effect of sequencing depth on fingerprint stability

Fingerprints were generated from accession CRR1173463 at 1×, 2×, 3×, 4×, 5×, 10×, 15×, and 20× genome coverage and compared across coverage levels Fig. 2A. Similarity values were highest when query and database fingerprints were generated at the same coverage, whereas mismatched coverage levels reduced similarity. This result supports the use of matched coverage during database construction and query processing.

### Data records

**Figure 3:**
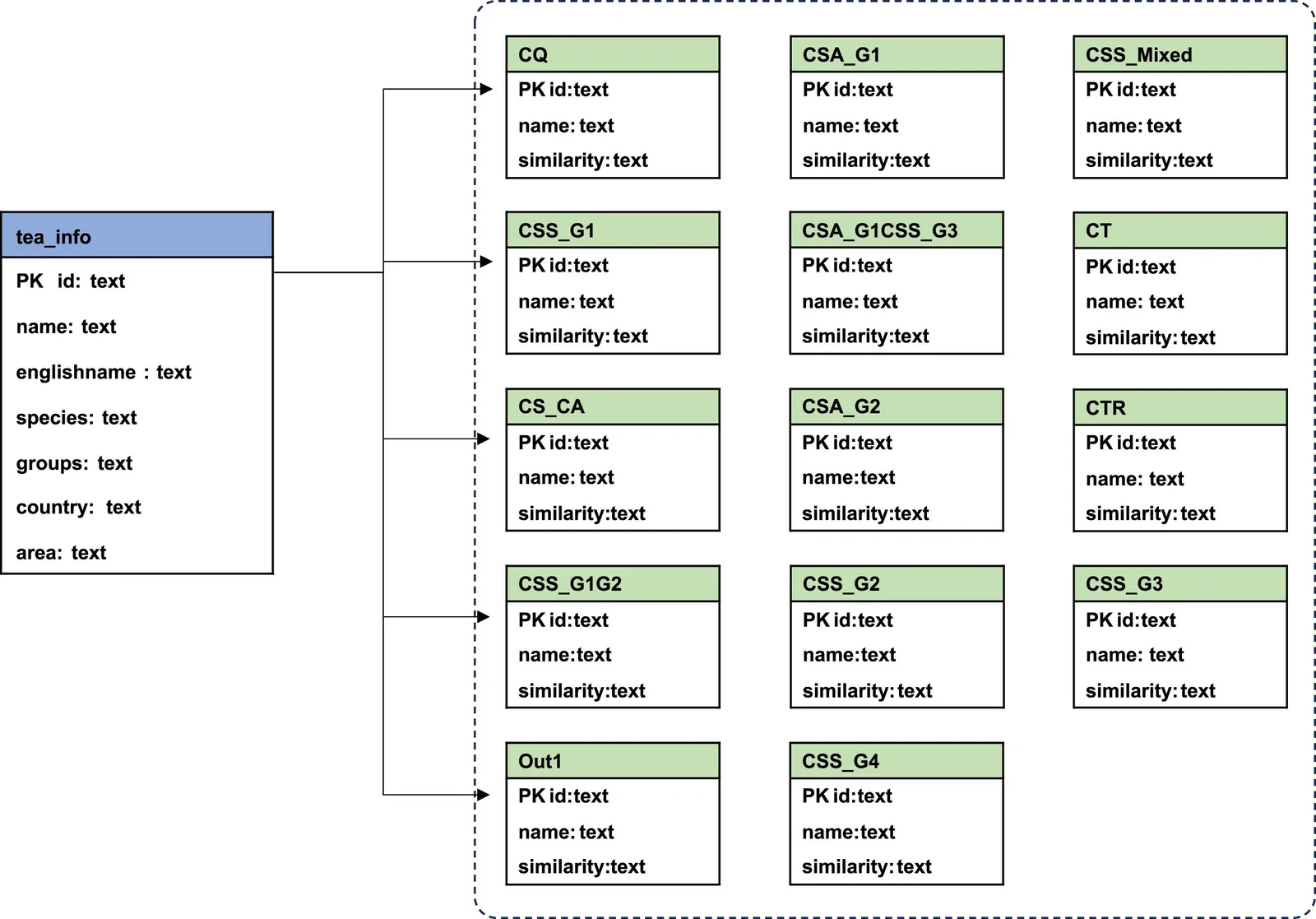
Architecture of the standardized Cam*K*-DB database. Cam*K*-DB contains 418 standardized *Camellia accessions* represented by MIKE-derived MinHash fingerprints generated at 5× genome coverage. The SQLite database includes a central tea_info metadata table linked to group-specific fingerprint tables. The fingerprint field stores encoded MIKE prefix-MinHash features used for Jaccard similarity calculation, rather than precomputed pairwise similarity values.

Cam*K-*DB is distributed as a single SQLite database file (TEA.db), which stores all derived MinHash fingerprints and sample-level metadata for the 418 *Camellia* accessions. The core data of Cam*K-*DB (∼2.258 GB [2,258,092,032 bytes]) consists of MinHash fingerprints derived from *k-*mers using Mike-generated “.jac” files that encode MinHash signatures as integer–count pairs. Each sample’s signature includes up to 1,048,576 hash buckets, capturing the presence and frequency of the hashed elements. This approach enables the efficient representation and comparison of genomic features through MinHash-based similarity estimation. This database was designed for efficient storage and retrieval. It uses a master table (tea_info) containing general information about each tea tree sample (ID, name, classification, and origin). The tea_info table contains one record for each of the 418 Camellia accessions and includes seven fields: id, name, English name, species, groups, country, and area. The 14 group-specific tables correspond to the genetic groups CQ, CSA.G1, CSA.G1–CSS.G3, CSA.G2, CSS.G1, CSS.G1–G2, CSS.G2, CSS.G3, CSS.G4, CSS. Mixed, CS.CA, CT, CTR, and Out1 (Fig. 3, Supplementary Table S2). Group names are recorded using dot notation in the tea_info table, whereas underscore-compatible names are used for the corresponding SQLite tables. The TEA.db file, together with the complete Cam*K-*DB source code, sample data, and GUI resources, is archived at Zenodo [22].

### Technical validation

We evaluated the reliability and biological interpretability of the standardized Cam*K-*DB release through a series of complementary validation analyses. These included accession-level self-identification, artificial two-source mixed-sample detection, concordance between Cam*K-*DB similarity and phylogenetic distance, out-of-database query assessment, sequencing-depth robustness, sketch-size sensitivity, and group-level representation.

### Self-identification of accessions represented in Cam*K-*DB

As an internal consistency assessment, we first assessed whether Cam*K-*DB could correctly recover accessions already represented in the standardized TEA.db database. Ten accessions, including PT57B, SRR12265461, SRR12265466, Y12A, Y28A, Y30A, Y31A, Y38A, YN6A, and ZH5A, were selected as query samples. For each accession, resequencing reads Whole Genome Sequencing (WGS) were queried against TEA.db. In all ten cases, the top-ranked database hit corresponded to the query accession itself (Table 1). Primary similarity values ranged from 0.544 to 0.607, with a mean of 0.583. By contrast, the second-ranked hits showed lower similarity values, ranging from 0.275 to 0.373, with a mean of 0.311. The mean top1–top2 similarity margin was 0.272, indicating clear separation between the matched accession and the closest non-identical database neighbor. These results demonstrate that, under the standardized 5× fingerprinting strategy, Cam*K-*DB can reliably recover represented accessions and distinguish them from related but non-identical entries in the database.

**Table 1:** Self-identification performance of standardized Cam*K*-DB for accessions represented in TEA.db. Ten accessions represented in the standardized database were queried against TEA.db using the same 5× MIKE fingerprint-generation workflow. In all cases, the top-ranked hit corresponded to the same accession as the query sample.

*Sample*	Primary match		Secondary match	
	ID	Similarity	ID	Similarity
PT57B	PT57B	0.573086	PT44B	0.275166
SRR12265461	SRR12265461	0.605142	K78A	0.301827
SRR12265466	SRR12265466	0.59301	K78A	0.311545
Y12A	Y12A	0.559994	K47A	0.318512
Y28A	Y28A	0.604648	Y15A	0.372634
Y30A	Y30A	0.603477	K78A	0.314028
Y31A	Y31A	0.579548	K47A	0.28647
Y38A	Y38A	0.607403	K78A	0.309742
YN6A	YN6A	0.543805	YN4A	0.33486
ZH5A	ZH5A	0.559406	K78A	0.289278

### Artificial two-source mixed-sample detection

We further evaluated whether Cam*K-*DB could recover known genomic contributors from artificial mixed-read datasets. Five database accessions were selected, and pairwise combinations of their resequencing reads were merged to generate ten two-source mixed datasets. The corresponding “.jac” fingerprints were generated and queried against TEA.db using the same standardized comparison framework. All ten artificial mixtures indicated two highest-ranked database records corresponded to the two accessions used to construct the mixed query dataset (Table 2). Across the ten mixtures, primary similarity values ranged from 0.386 to 0.504, with a mean of 0.434, whereas secondary similarity values ranged from 0.372 to 0.489, with a mean of 0.418. The mean top1–top2 margin was small, 0.015, consistent with the expectation that both source accessions contribute comparable genomic signals to the mixed-read fingerprint.

**Table 2:** Cam*K*-DB query results for artificial two-source mixed-read datasets. Artificial mixed-read datasets were generated by combining reads from pairs of accessions represented in the standardized Cam*K*-DB database. For all ten mixtures, the two highest-ranked database records corresponded to the two source accessions used to generate the query dataset. This result demonstrates that Cam*K*-DB can recover dual-source signals from artificial mixed-read data when both source accessions are present in the database. This analysis should be interpreted as mixed-sample signal detection and not as formal biological parentage or hybrid-origin inference.

*Sample1*	*Sample2*	Primary match		Secondary match	
		ID	Similarity	ID	Similarity
Y12A	PT57B	Y12A	0.385688	PT57B	0.377445
Y12A	Y28A	Y28A	0.500619	Y12A	0.481872
Y12A	Y38A	Y38A	0.504148	Y12A	0.482033
Y12A	YN6A	Y12A	0.414585	YN6A	0.410418
Y28A	PT57B	Y28A	0.39659	PT57B	0.373197
Y28A	Y38A	Y38A	0.492228	Y28A	0.488699
Y28A	YN6A	Y28A	0.426494	YN6A	0.406801
Y38A	PT57B	Y38A	0.398635	PT57B	0.371728
Y38A	YN6A	Y38A	0.427172	YN6A	0.403255
YN6A	PT57B	YN6A	0.389391	PT57B	0.385629

This analysis demonstrates that Cam*K-*DB can recover dual-source signals from artificial mixed-read datasets when both contributing accessions are represented in the database.

However, this *in silico* assessment should be interpreted as mixed-sample or two-source signal detection rather than formal biological parentage or hybrid-origin inference. A physical mixture of reads from two accessions does not recapitulate the genomic structure of a true biological hybrid, because hybrids inherit recombined haplotypes generated through meiosis and segregation. Therefore, formal inference of parentage, hybrid origin, or genealogical ancestry would require validated hybrid material, segregating progeny, or inheritance-aware analytical models.

### Concordance with phylogenetic distance and out-of-database query behavior

To assess whether CamK-DB similarity reflected biologically meaningful genetic relationships, we compared the similarity scores with phylogenetic distances obtained from the previously published Camellia phylogenetic tree of Kong et al. [12]. Most accessions showed negative correlations between CamK-DB similarity and phylogenetic distance, indicating that genetically closer accessions generally exhibited higher fingerprint similarity (Fig. 2D). These results support the biological relevance of CamK-DB similarity scores. We further assessed Cam*K-*DB behavior using an out-of-database query sample, SRR11672521, not being in the standardized TEA.db release. Although the exact accession was absent from the database, higher Cam*K-*DB similarity was associated with lower phylogenetic distance to database accessions (*r* = −0.37, *P* = 4.79 × 10⁻¹⁵; Fig. 2C). This result indicates that, when an exact database match is unavailable, CamK-DB can identify the closest available genomic relatives. Such results should be interpreted as nearest-neighbor relationships rather than definitive accession identification.

### Sequencing-depth robustness and sketch-size sensitivity

Because MIKE-derived fingerprints depend on the subset of *k-*mer-derived elements recovered from sequencing reads, sequencing depth can influence the resulting fingerprint composition and Jaccard similarity estimates. We therefore evaluated the effect of coverage matching on fingerprint similarity. Coverage-comparison analysis showed that similarity was highest when query and database fingerprints were generated at matched genome coverage and decreased when coverage differed (Fig. 2A and B). This result supports the use of standardized 5× genome-coverage fingerprints for both database construction and query processing, thereby reducing technical bias caused by sequencing-depth heterogeneity.

We also evaluated the effect of sketch size on similarity-estimation accuracy and runtime. Sketch/pre_cnt values of 100, 500, 1,000, 2,000, 5,000, 8,000, and 10,000 were tested by comparing sketched similarity estimates with full fingerprint comparisons. Small sketch sizes produced higher RMSE, whereas increasing the sketch size substantially reduced estimation error. The RMSE distribution reached a low and stable level around sketch/pre_cnt = 2000, while larger sketch sizes did not provide proportional improvement in accuracy. Runtime remained comparable among moderate sketch sizes but increased markedly at very large values. Together, these results support sketch/pre_cnt = 2000 as an appropriate balance between similarity-estimation accuracy and computational efficiency for standardized Cam*K-*DB queries (Supplementary Fig. S2A and B).

### Group-level representation of the standardized database

Finally, we evaluated the distribution of accessions across *Camellia* groups in the standardized 418-accession database. The retained accessions were distributed across multiple taxonomic and genetic groups, with a median group size of 16 accessions (Supplementary Fig. S2C). This indicates that the standardized Cam*K-*DB release preserves broad group-level representation for reference-free accession comparison. Nevertheless, some groups remain less densely represented than others, and future database expansions should prioritize additional sampling of underrepresented groups to improve resolution across the full diversity of *Camellia* germplasm.

## Methods

### Source resequencing datasets and licensing

Cam*K-*DB integrates whole-genome resequencing data from 418 *Camellia* accessions. Newly generated data for 320 accessions were produced as part of a recent large-scale genomic survey of *Camellia* germplasm and are deposited in the GSA database [23] (BioProject, accession PRJCA026608 [12]). Previously published resequencing data for 415 accessions were downloaded from the GSA and NCBI SRA repositories [23, 25] under BioProjects PRJNA665594 [15], and in the GSA database under the accessions PRJCA003090 [15], PRJNA646044 [16], PRJNA716079 [17], and PRJNA597714 [18]. All of these datasets were obtained from repositories that explicitly permit reuse for research purposes. In Cam*K-*DB, we only redistribute derived MinHash fingerprints, the SQLite database file, and sample-level metadata; we do not redistribute the original raw reads from third-party studies, thereby complying with the terms and conditions of the original data providers (Supplementary Tables S1 and  S5).

### Sequencing data processing

For the construction of database, this study utilized a dataset of 418 *Camellia* accessions, based on our recently published research on *Camellia’s* evolutionary history [12]. This publication encompassed an expanded dataset that included 802 newly sequenced reads and 523 previously sequenced reads. *Camellia* accessions selected in this work originated from 13 countries: Azerbaijan, China, Georgia, Japan, Kenya, Laos, Myanmar, Sri Lanka, and the USA.

However, we used 320 newly sequenced accessions and 98 publicly available accessions (total *n* = 418) after QC for Cam*K-*DB. The dataset included diverse *Camellia* species: *C. oleifera, C. quinquelocularis, C. sasanqua, C. sinensis* var. *assamica, C. sinensis* var. *pubilimba, C. sinensis* var. *sinensis, C. tachangensis* var. *remotiserrata*, and *C. taliensis*. Importantly, the *C. sinensis* accessions represent a mix of wild, elite, and landrace varieties, whereas the remaining species were included as wild samples. Among 418 selected accessions, we used 320 newly sequenced *Camellia* accessions from multiple countries, including Azerbaijan, China, Georgia, Iran, Laos, Myanmar, Sri Lanka, and Russia, from our sequenced resource (originally sequencing depth ranging from 9 to 23). Genomic DNA from the leaves was extracted using the DNeasy Plant Mini Kit (Qiagen), followed by 5 μg to construct libraries with an insert size of 500 bp. Each sample consisted of 150 bp paired-end reads on the DNBSEQ-T7 platform (Beijing Genomics Institute). Furthermore, to increase the sample diversity, resequencing data accessions of *Camellia* were downloaded from public databases, i.e. the National Genomics Data Center [24] and the National Center for Biotechnology Information [25], from the given research [15–19]. Raw sequencing reads were down sampled to 5× subjected to quality control and removal of low-quality reads and adapter sequences by Trimmomatic with default parameters, i.e. Trimmomatic PE -phred33 ILLUMINACLIP:adapters.fa:2:30:10 LEADING:3 TRAILING:3 SLIDINGWINDOW:4:15 MINLEN:50 [20]. The resulting high-quality reads were processed using the MIKE tool to generate minimum hash signatures for each sample (Supplementary Table S1). Across the 418 datasets, the mean percentages of Q20 and Q30 bases were 97.62% and 92.56%, respectively, while the mean GC content was 39.71%. The Q20, Q30, and GC-content values ranged from 92.95% to 99.12%, 85.48% to 96.83%, and 37.99% to 45.89%, respectively (Supplementary Tables S3 and S4). The number of retained reads per accession ranged from ~50.39 million to 102.14 million, with an average of 100.44 million reads. The corresponding sequence yield was 15 Gb on average per accession. Following adapter trimming and quality filtering, the high-quality reads were aligned to the chromosomal-scale *C. sinensis* TGY genome (∼3.15 Gb) [15] by BWA [21] with default parameters. The alignment results demonstrated high sequence similarity and minimal contamination. Genome coverage was high across all samples, exceeding 88% on average, with several samples achieving a genome anchorage >94% (Supplementary Tables S3 and  S4).

### User guide and input data specifications

Cam*K-*DB provides both command-line and graphical interfaces for reference-free identification of *Camellia* accessions using MIKE-derived MinHash fingerprints. The current standardized release of Cam*K-*DB contains 418 *Camellia* accessions, each processed under the same 5× genome-coverage sampling strategy. This standardization is important because MIKE/Jaccard similarity can be affected by sequencing-depth differences between the database fingerprint and the query fingerprint. Therefore, query samples should be processed using the same 5× coverage-normalized workflow before comparison with the TEA.db database.

### Input data

Cam*K-*DB accepts either raw sequencing files or precomputed MIKE fingerprint files. Supported input formats include FASTQ/FASTA files and MIKE-generated “.jac” files. The current release was validated primarily using short-read whole-genome resequencing data generated from Illumina-compatible or DNBSEQ paired-end platforms. For reliable comparison with the standardized TEA.db database, users should provide high-quality reads with sufficient usable depth to allow downsampling to 5× genome coverage. Query datasets with <5× usable coverage after quality filtering are not recommended for high-confidence identification.

### Read preprocessing and coverage normalization

Before fingerprint generation, raw reads should be filtered to remove adapter contamination, low-quality bases, and short reads. In this study, reads were processed using quality-control steps equivalent to adapter trimming, low-quality base removal, sliding-window quality filtering, and exclusion of reads <50 bp. After quality filtering, query reads should be downsampled to 5× genome coverage using the same genome-size estimate applied during database construction. This step is required because fingerprints generated at unmatched coverage levels may show reduced similarity even when they originate from the same accession.

### MIKE fingerprint generation

After preprocessing and 5× downsampling, query reads are converted into MIKE “.jac” fingerprint files using the same parameters used for database construction. The *k-*mer length should be set to *k* = 21, following the MIKE framework. The recommended sketch/pre_cnt value for Cam*K-*DB queries is 2000, based on sensitivity analysis showing a favorable balance between similarity-estimation accuracy and runtime. Query fingerprints should therefore be generated using *k* = 21, 5× genome coverage, and sketch/pre_cnt = 2000 to ensure consistency with the standardized TEA.db database.

### Command-line query

The command-line interface is suitable for batch analysis and high-throughput screening. A typical query uses the TEA.db database, a query “.jac” file, and the recommended sketch/pre_cnt value:

“query_db TEA.db query_sample.jac 2000”

The program compares the query fingerprint against the standardized Cam*K-*DB database and returns the top-ranked accessions according to Jaccard similarity. The output table includes accession identifiers, names, taxonomic or genetic group information, geographic metadata, and similarity scores.

### Graphical user interface

The GUI provides an interactive workflow for users who prefer not to run command-line queries. Users first select the standardized TEA.db database and then provide either a precomputed “.jac” fingerprint or a processed query file for fingerprint generation. After running the query, the GUI displays the top-ranked database accessions with their metadata and similarity scores. Results can be sorted by similarity to identify the closest database matches Supplementary Fig. S1.

### Interpretation of query results

Cam*K-*DB should be interpreted as a nearest-neighbor genomic fingerprinting system. A high top-ranked similarity score together with a clear separation between the first and second hits supports a confident exact or near-clonal match, provided that the accession is represented in the database. Moderate similarity may indicate a close relative or accession from the same genetic group. Low similarity or a small top1–top2 margin suggests that the query accession may be absent from the current database or poorly represented by available database accessions.

For samples whose exact identity or close relatives are not present in Cam*K-*DB, the top-ranked hits should not be interpreted as definitive identity assignments. Instead, they represent the closest available genomic neighbors within the standardized database. Users should therefore consider both the absolute similarity value and the top-hit margin when interpreting results.

## Limitations

The current standardized Cam*K-*DB release contains 418 accessions processed under a matched 5× genome-coverage strategy. Query fingerprints generated at arbitrary or unmatched coverage should not be directly compared with the standardized database, because coverage differences can reduce Jaccard similarity. Formal biological parentage or hybrid-origin inference is not supported by the current release; mixed-read analyses should be interpreted only as dual-source or mixed-sample signal detection. Future releases may incorporate additional accessions and further optimize parameters for specific *Camellia* groups or sequencing platforms.

## Availability of source code and requirements

Project name: CamK-DB

Project version: v1.0.1

Project home page: https://github.com/sc-zhang/CamK-DB

Operating system(s): Windows 10/11 and Linux (x86_64; glibc ≥ 2.27)

Programming language: C++17 and Python

Other requirements: SQLite3 and Qt 6; CMake ≥ 3.24 is required for source compilation. MIKE-generated .jac fingerprint files are required for CamK-DB queries and input samples should be down samples to 5x depth for authenticated comparisons.

License: MIT License

RRID: Not applicable

bio.tools ID: Not applicable

All software and analytical scripts developed for this study are publicly available under open-source licenses via GitHub repositories. Supporting software resources are available through the MIKE [26] and CamK-DB [27] GitHub repositories.

## Additional files

**Supplementary Figure S1**. CamK-DB graphical user interface workflow. Overview of the graphical workflow for selecting a query input, loading the standardized TEA.db database, processing or selecting a MIKE .jac fingerprint, performing the similarity search, and displaying the top-ranked accessions together with their identifiers, metadata, and Jaccard similarity values.

**Supplementary Figure S2**. Effects of sketch size on CamK-DB performance and group-level representation. (A) Root mean square error between similarity estimates generated using different sketch/pre_cnt values and those obtained from the full fingerprint comparison. (B) Query runtime across the tested sketch/pre_cnt values. The combined accuracy and runtime analyses support sketch/pre_cnt = 2000 as the recommended setting for CamK-DB queries. (C) Distribution of the 418 standardized accessions among the 14 genetic groups represented in CamK-DB. Group sizes ranged from 1 to 106 accessions, with a median of 16, demonstrating broad but uneven representation across groups.

**Supplementary Table S1**. Metadata and sequencing information for the *Camellia* accessions included in CamK-DB. List of the 418 accessions used for database construction, including accession identifiers, names, taxonomic assignments, genetic-group classifications, geographic origins, sequencing-data sources, and associated database information.

**Supplementary Table S2**. Distribution of CamK-DB accessions among genetic groups and SQLite tables. Summary of the 14 genetic groups represented in the standardized database, including the corresponding SQLite table names and the number and percentage of accessions assigned to each group.

**Supplementary Table S3**. Sequencing-read quality statistics for the 418 *Camellia* accessions. Accession-level quality-control metrics, including the number of retained reads, total retained bases, Q20 and Q30 percentages, GC content, and estimated post-processing sequencing depth.

**Supplementary Table S4**. Reference-mapping and genome-coverage statistics for the 418 Camellia accessions. Accession-level alignment statistics generated against the *C. sinensis* TGY reference genome, including mapping rate, mapped-read counts, genome-coverage breadth, and other relevant alignment-quality measurements.

**Supplementary Table S5**. List of Archive_Metadata, downloading links, and relevant information.

## Competing interests

The authors declare that they have no competing interests.

## Supplementary Material

giag088_Supplemental_Files

giag088_Authors_Response_To_Reviewer_Comments_Original_Submission

giag088_GIGA-D-25-00529_Original_Submission

giag088_GIGA-D-25-00529_Revision_1

giag088_Reviewer_1_Report_Original_Submission Reviewer 1 -- 1/20/2026

giag088_Reviewer_1_Report_Revision_1 Reviewer 1 -- 7/28/2026

giag088_Reviewer_2_Report_Original_Submission Reviewer 2 -- 1/27/2026

giag088_Reviewer_2_Report_Revision_1 Reviewer 2 -- 7/31/2026

## Data Availability

The CamK-DB dataset, including the TEA.db SQLite database, per-sample MinHash fingerprint files, and the main program, is archived in Zenodo [22]. Newly generated raw whole-genome resequencing data are publicly available in the Genome Sequence Archive (GSA) [23] under BioProject accession PRJCA026608. Previously published resequencing data used to construct CamK-DB are available under BioProject accessions PRJNA665594, PRJCA003090, PRJNA646044, PRJNA716079, and PRJNA597714 through the GSA and NCBI Sequence Read Archive (SRA) [23,25].
