# Supplementary material for: CamK-DB: A k-mer MinHash fingerprint database for reference-free genotyping of Camellia accessions
Source: Gigascience. 2026 Aug 21;15:giag088. doi: 10.1093/gigascience/giag088 (PMC13548848; doi:10.1093/gigascience/giag088)
Supplement: Supplementary file 1 [file giag088_authors_response_to_reviewer_comments_original_submission.pdf]

Author's Response To Reviewer Comments

Response to Reviewers
Manuscript ID: GIGA-D-25-00529
Title: CamK-DB: A k-mer MinHash fingerprint database for reference-free genotyping of Camellia accessions
Dear Editor and Reviewers,
We sincerely thank the Editor and both reviewers for their constructive comments and helpful suggestions. We have substantially revised the manuscript to improve its methodological rigor, parameter justification, database transparency, reproducibility, and practical user guidance. The major revisions include: (i) We redownloaded ~13TB raw data and reconstructed the final CamK-DB release as a 5× coverage-standardized database; (ii) clarification that 735 candidate resequencing datasets were initially curated, of which 418 accessions passed the quality and coverage requirements and were retained in the final standardized TEA.db database; (iii) expanded explanation of the MIKE/MinHash algorithm, including prefix/suffix grouping and exact matching of fingerprint elements; (iv) addition of coverage-matching and same-coverage replicate stability analyses; (v) addition of sketch-size sensitivity analysis supporting sketch/pre_cnt = 2000; (vi) revision of the mixed-read experiment as mixed-sample or dual-source signal detection rather than formal parentage inference; (vii) improved validation using phylogenetic-distance concordance and out-of-database query interpretation; and (viii) redesigned figures and expanded user-guide instructions.
We have also updated the public repository, supplementary tables, and figure files to reflect the revised database construction and validation strategy.
Best regards,
Xingtan Zhang
Research Professor
Agricultural Genomics Institute at Shenzhen
Shenzhen, 518120, Guangdong, China
E-mail: zhangxingtan@caas.cn

Reviewer #1
We sincerely thank Reviewer 1 for the constructive comments and valuable suggestions, which substantially improved the scientific clarity and presentation of the manuscript. We kindly invite the reviewer to examine the revised main and supplementary figures, which have been updated in response to these comments.

Major Comment 1: "Justification for Algorithm Selection (Lines 66-68): The manuscript mentions several hashing-based techniques (MinHash, Bloom filter, Count-Min Sketch) but provides no rationale for selecting MinHash over others, particularly Bloom filters, which are also known for efficiency in handling large genomic datasets. Please clarify the comparative advantages of MinHash in this specific context (e.g., for similarity estimation via Jaccard index vs. membership queries) and whether a formal comparison was conducted to justify this choice as optimal for the stated objectives of rapid, accurate germplasm identification and comparison."
Response: We thank the reviewer for this helpful suggestion. We have revised the Background and Methods sections to clarify why MinHash was selected for CamK-DB. The objective of CamK-DB is rapid similarity estimation and accession ranking, rather than membership querying or abundance estimation. In the MIKE framework, reads are decomposed into k-mers, deduplicated k-mer information is grouped by prefix, and each fingerprint element is represented as a `(prefix, minhash(suffix))` pair. Jaccard similarity is then calculated by comparing these MIKE-derived fingerprint-element sets between query and database samples. MinHash is well suited behind the rationale of our database because it provides a compact approximation of Jaccard set resemblance. In contrast, Bloom filters are mainly designed for approximate membership queries and may introduce false-positive overlap when adapted for similarity estimation, whereas Count-Min Sketch is mainly used for approximate frequency estimation rather than direct set-similarity comparison. Therefore, although these methods are useful for other k-mer applications, MinHash

is more directly aligned with CamK-DB's goal of reference-free similarity ranking. We did not perform a formal head-to-head benchmark against Bloom filters or Count-Min Sketch in this study. The choice was task-driven and based on MIKE, which was specifically developed for fast MinHash-based similarity estimation from sequencing reads Lines 63-77 and121-152.

Major Comment 2: "Choice of k-mer Size (k=21) (Lines 98-100): The selection of k=21 is stated as the default/recommended setting in MIKE but is not biologically or computationally justified within this study. Please provide the reasoning behind this specific value. Were other k-mer lengths evaluated (e.g., 15, 31, 51)? If so, please present a brief analysis (e.g., in supplementary material) showing how different k values affect discrimination power, runtime, or database size. If not, please acknowledge this as a limitation or standard parameter from prior work and discuss its suitability for tea genomes."

Response: We thank the reviewer for this helpful comment. We have revised the Methods section to better justify the use of k=21. This parameter was selected based on the original MIKE study, where simulated datasets with 40× coverage were tested using various k=17, 19, 21, 25, and 27. That analysis showed that k=21 provided a stable balance between similarity-estimation accuracy and robustness, while larger k values showed increasing deviation, likely because longer k-mers are more prone to sequencing errors and per nucleotide variation. We also added a Camellia-specific explanation. Tea genomes are large, highly heterozygous, and repeat-rich; therefore, shorter k-mers may produce non-specific matches in repetitive regions, whereas longer k-mers may reduce sensitivity because single-base differences disrupt the entire k-mer. Thus, k=21 was retained as the MIKE-supported criterion for CamK-DB fingerprint generation. We did not perform a new CamK-DB-specific k-mer sweep in this revision as it was pre-validated in our previous work Lines 106-119 and 381-388..

Major Comment 3: "Use of Unique k-mers and Data Heterogeneity (Lines 100-103; 225-234): The manuscript states that unique k-mers are used to build MinHash fingerprints. Given the noted variability in sequencing depth, data volume, mapping rate, and genome coverage among the 735 accessions (Table S1), it is crucial to address: λ What is the approximate count of unique k-mers per sample, and how does it vary? λ How does variability in sequencing depth and data quality impact the number and stability of unique k-mers extracted? λ What normalization or standardization steps (if any) were applied to mitigate biases introduced by such technical heterogeneity before sketch generation and similarity calculation?"

Response: We thank the reviewer for raising this important point. We have revised the manuscript to clarify the terminology. Originally the use of "unique k-mers" was potentially misleading. CamK-DB does not use unique k-mers in the sense of biological single-copy, accession-specific, or sample-private k-mers. Instead, MIKE first deduplicates k-mer sequence identities and then converts them into compact prefix-minhash fingerprint elements. Specifically, reads are decomposed into 21-mers. Each 21-mer is divided into a 10-base prefix and an 11-base suffix. K-mers with the same prefix are grouped, and MIKE records a minimized hash-derived value from the suffix group. Because the prefix length is 10 bases, the theoretical feature space is fixed at 4^{10} = 1,048,576 possible prefix groups. Thus, the `.jac` fingerprint is a compact list of prefix-minhash feature values rather than a direct list of raw unique k-mers. We have revised the Methods section to explain this clearly. We also addressed the reviewer's concern about sequencing-depth heterogeneity. Because different sequencing depths can lead to estimation of different subsets of k-mers before minimization, fingerprints generated from unequal coverage may show reduced similarity even for the same accession. We therefore tested fingerprints generated at different coverage levels and found that similarity was highest when query and database fingerprints were generated at matched coverage. To mitigate this bias, we rebuilt the final CamK-DB release using standardized 5× genome-coverage sampling. Of the 735 candidate datasets initially curated, 418 accessions met the quality and coverage requirements for standardized 5× fingerprint generation and were retained in the final TEA.db database. Query samples are now recommended to be downsampled to 5× before MIKE fingerprint generation. Lines 119-137, Table S1,S5, and S3.

Major Comment 4 : Jaccard Similarity Calculation and Its Robustness (Lines 107-115):
The method for calculating Jaccard similarity based on shared unique k-mers requires further elaboration:
λ How is a "shared unique k-mer" defined? Does it require an exact sequence match (presumably yes for k-mers), or are mismatches allowed?
λ Are the sets S1 and S2 (the unique k-mer sets from two samples) required to be of comparable size? If two samples have vastly different numbers of unique k-mers (e.g., due to different coverage), how does this affect the Jaccard index? Have the authors evaluated the sensitivity of the similarity score to such disparities?
Please describe any preprocessing, filtering, or scaling procedures implemented to ensure fair comparisons between samples with differing k-mer set sizes.

Response: We thank the reviewer for this important comment. We have revised the manuscript to clarify that CamK-DB does not calculate Jaccard similarity from raw "unique k-mer" sets in the biological sense of single-copy or accession-specific k-mers. Instead, CamK-DB uses MIKE-derived fingerprint-element sets. Briefly, reads are decomposed into 21-mers, identical 21-mers are deduplicated, and each 21-mer is divided into a 10-base prefix and an 11-base suffix. K-mers with the same prefix are grouped, and MIKE records a minimized hash-derived value from the suffix group. Each fingerprint element is therefore represented as a (prefix, minhash(suffix)) pair. A shared element requires an exact match of both the prefix and the minhash(suffix) value between the query and database fingerprint. However, mismatches are not allowed. In the revised manuscript, S_1 and S_2 are now defined as MIKE-derived fingerprint-element sets rather than raw unique k-mer sets, and Jaccard similarity is calculated as $|S_1 \cap S_2| / |S_1 \cup S_2|$.
To address the k-mer sizes, coverage related nuisance on Jaccard's index, we also applied a test to validate that if the coverage of reads should be comparable, We tested this by generating fingerprints from the same accession at different genome coverage levels [1,2,3,4,5,10,15,20] fold of genome size, then used these dataset to extract minhash fingerprint, which were used to construct database. The results showed that similarity was highest when query and database fingerprints were generated at the same coverage and decreased when coverage differed.
To reduce this technical bias and ensure fair comparison, we reconstructed the final CamK-DB release using standardized 5× genome-coverage sampling. Of the 735 candidate Camellia resequencing datasets initially curated, 418 accessions met the quality and coverage requirements for standardized 5× fingerprint generation and were retained in the final TEA.db database. Query samples are now recommended to be downsampled to 5× before MIKE fingerprint generation to ensure comparability with the standardized database. Lines 121-152, 187-192, 270-279, and 372-380.
Major Comment 5: "Rationale for Sketch Size (1000) (Lines 118-120): The use of a"1000-sketch size" is mentioned without justification. This parameter controls the resolution and accuracy of the MinHash approximation. λ What was the basis for choosing a sketch size of 1000? Was a sensitivity analysis performed with different sketch sizes (e.g., 500, 2000, 5000)? λ Please provide evidence or references showing that a size of 1000 provides an optimal balance between accuracy, storage, and computational speed for this specific dataset and application."
Response: We agree that this parameter required empirical justification. Therefore, we performed a sketch-size sensitivity analysis using values of 100, 500, 1000, 2000, 5000, 8000, and 10000. For each setting, we compared the sketched similarity estimates with the full fingerprint comparison and calculated RMSE, while also recording runtime. The results showed that RMSE decreased substantially as sketch size increased from very small values and became stable around 2000. Larger sketch sizes increased runtime without proportional improvement in accuracy. Based on this analysis, we revised the manuscript to recommend sketch/pre_cnt=2000 for high-confidence CamK-DB analyses. The abstract, Methods, User Guide, figure labels, and supplementary results have been updated accordingly Lines 162-172, 280-290, and 433-441.
Major Comment 6: "Clarity on Input Requirements and Tool Robustness (User Guide Section): The user guide effectively describes how to use CamK-DB but lacks critical specifications on what data is suitable as input. Minimum Requirements: Please explicitly state the required sequencing platform (or compatibility), recommended minimum sequencing depth, and necessary read length for reliable analysis. Data Quality Thresholds: Specify the tool's tolerances or requirements for input data quality (e.g., Q-score thresholds, adapter contamination levels). What QC steps are mandatory before running MIKE to generate the".jac" file? Performance under Variable Depth: How do sequencing depth and coverage influence runtime and, more importantly, the accuracy of identification (e.g., top-hit confidence, similarity score stability)? A brief analysis or statement on the tool's robustness to suboptimal data would greatly enhance its practical utility guide."
Response: We have expanded the User Guide by adding a dedicated "Input Data Specifications" subsection. The revised manuscript now states that CamK-DB supports FASTQ/FASTA files and precomputed MIKE.jac fingerprints, and that the standardized release was validated primarily using short-read whole-genome resequencing data. We specify the required preprocessing steps, including adapter removal, low-quality read trimming, and removal of reads shorter than 50 bp. We further state that query data should be downsampled to 5× genome coverage before MIKE fingerprint generation to ensure comparability with the standardized TEA.db database. The revised User Guide also explains that fingerprints generated from substantially different coverage levels may show reduced similarity, even when they originate from the same accession. These additions provide practical guidance on suitable input data, quality control, coverage normalization, and interpretation of results Lines 354-388 and 405-426.

Minor Comment 1: “Dataset Representativeness and Selection Criteria (Line 188): Please state the average sequencing depth for the entire set of 735 accessions. More importantly, justify the selection of these specific 735 accessions. Do they comprehensively represent the genetic, geographical, and phenotypic diversity of cultivated and wild Camellia? A brief discussion on the representativeness and potential biases of the dataset would be valuable.”

Response: We have revised the dataset description for 418 accessions retained in the final 5× standardized CamK-DB release. We added sequencing-quality attributes and other related information to Supplementary Table S1. We also added group-level representation information for the final 418 retained accessions. The revised Discussion acknowledges that although the retained accessions span multiple Camellia species, genetic groups, and geographic origins, the current standardized release is not exhaustive and may underrepresent some geographic regions or rare germplasm groups. Future releases will incorporate additional accessions that meet the 5× standardized fingerprinting requirements Lines 291-299, 315-329, 418-426, and 439-451.

Minor Comment 2: “Terminology Accuracy (Lines 217-220): The phrase”deep resequencing data with more than 8× sequencing depth” is misleading. In current genomics standards, “deep” resequencing typically implies coverage ≥30x. Please rephrase to “resequencing data with a minimum depth of 8×” or similar to avoid overstatement.”

Response: We agree and have replaced “deep resequencing data with more than 5× sequencing depth” with “resequencing data with a minimum depth of 5×” or equivalent wording throughout the manuscript Lines 327-340.

Minor Comment 3: “Completeness of Table S1: Table S1 appears to contain detailed metrics only for newly sequenced data, leaving fields blank for publicly sourced accessions. To ensure transparency and reproducibility, please supplement the table with available metrics (e.g., SRA accession-linked depth, read counts if obtainable) for all 735 accessions or clearly note the source of each datum.”

Response: We have expanded Supplementary Table S1, S3 and S5 to include available metadata and quality information for all 418 candidate datasets, including public accession identifiers, source references, estimated sequencing depth or read count where available, and retained/excluded status in the final standardized database.

Minor Comment 4: “Expanded User Guide: As noted in Major Comment 6, the User Guide section should be expanded to include a dedicated subsection on”Input Data Specifications” detailing platform, depth, quality control prerequisites, and file format requirements.”

Response: We have added the requested “Input Data Specifications” subsection to the User Guide. It describes accepted input formats, validated sequencing data type, quality-control requirements, 5× downsampling requirement, MIKE parameter settings, and interpretation of ranked results Lines 354-388 and 405-417.

Minor Comment 5: “Validity for Samples Outside the Database (Technical Validation): The validation convincingly shows performance for samples and mixtures within the database. Please comment briefly on the expected behavior or interpretive value when querying a sample whose true parents or close relatives are not in CamK-DB. How should users interpret the top hits in such scenarios?”

Response: We have added guidance for out-of-database queries. CamK-DB should be interpreted as a nearest-neighbor fingerprinting system. If the exact accession or a very close relative is absent from the database, the top hit should not be treated as a definitive identity assignment. Instead, the top-ranked accessions represent the closest available genomic neighbors under the current standardized database. We added an out-of-database query example showing that the highest-similarity database hits corresponded to accessions with relatively low genetic distance, supporting the use of CamK-DB for nearest-neighbor interpretation when exact matches are absent. Lines 255-269 and 405-417

Minor Comment 6: “Abbreviation Formatting (Lines 58-59): The first instance of an abbreviation (e.g., likely”” or similar from context) should be spelled out in full.”

Response: We have revised the manuscript to spell out abbreviations at first use and added an alphabetical abbreviation list Lines 54-61

Minor Comment 7: “Formatting Consistency: Line 103:”k-mers” - the ‘k’ should be italicized (k-mers). Line 107: “MinHash” - ensure consistent formatting; it should not be italicized if it is not standard elsewhere. Line 115: “S_2” should use subscript for the numeral. Add a space: “sample 2”.”

Response: We have corrected formatting throughout the manuscript. The term k-mer is now formatted consistently, MinHash is presented consistently, S1 and S2 are formatted with subscripts where appropriate, and “sample2” has been corrected to “sample 2”. Lines 63-80 and 106-152

Minor Comment 8: “Figure Reference (Lines 110-112): The text referencing”Figure 1” describes a

mathematical formula, but the current Figure 1 appears to depict database schema. Please verify all figure citations and ensure they accurately correspond to the described content. The formula for Jaccard similarity might be better placed in the main text or a dedicated methodology figure."

Response: We thank the reviewer for identifying this mismatch. We have handles in text figure citations and re-organized the figures. Lines 147-152 and 557-582.

Reviewer #2

We sincerely thank Reviewer 2 for the careful evaluation of our manuscript and the insightful recommendations that helped strengthen the methodology and technical validation. We kindly ask the reviewer to review the updated figures and supplementary materials, in which the requested analyses and clarifications have been incorporated.

Major Comment 1: "Justification of Algorithmic Parameters. The manuscript utilizes the MIKE algorithm with default parameters (k=21 and sketch size=1000) but falls short of providing a sufficient theoretical or empirical basis for these choices. Given that k-mer-based analyses are highly sensitive to parameter selection—particularly in the context of the tea genome, which is characterized by high heterozygosity and a high proportion of repetitive elements—the mere adoption of default settings is inadequate. The authors should elaborate on the underlying algorithmic mechanics, such as the specific hash functions employed, and discuss how the selected k-mer size balances the trade-off between specificity and the noise introduced by sequencing errors or genomic complexity."

Response: We agree and have substantially expanded the algorithmic description. The revised Methods now explain that MIKE decomposes reads into k-mers, encodes k-mers, groups them by prefix, and represents each group using the minimum hash value of the suffix. These encoded (prefix, minhash(suffix)) fingerprint elements are stored as .jac files and compared using Jaccard similarity. We also expanded the rationale for k=21, explaining the trade-off between short k-mers, which may be non-specific in repetitive genomes, and long k-mers, which are more sensitive to sequencing errors and heterozygous variation. In addition, we performed sketch-size sensitivity analysis and revised the recommended sketch/pre_cnt value from 1000 to 2000 based on RMSE and runtime results. The revised Figure 2 now illustrates the MIKE fingerprint-generation process in greater technical detail. Lines 106-172 and 557-565

Major Comment 2: "Rigor of Validation Strategy. The technical validation presented in the study is currently insufficient as it relies heavily on"self-identification," where accessions used to construct the database are simply re-queried against it. This circular approach essentially confirms the database's internal consistency but fails to demonstrate the tool's robustness and generalizability when faced with independent, external datasets. To truly validate the utility of CamK-DB in a real-world setting, the authors should perform cross-validation or, ideally, test the system with newly sequenced samples that were strictly excluded from the database construction process to prove it can accurately identify samples it has not "seen" before."

Response: We thank the reviewer for this important comment. We agree that self-identification alone is insufficient to demonstrate the broader utility of CamK-DB. In the revised manuscript, we now present self-identification only as an internal consistency control and added additional validation analyses to evaluate whether CamK-DB similarity scores reflect biological relatedness and whether the database can provide meaningful nearest-neighbor information for samples not included in TEA.db.

First, using the standardized 5× database, we queried ten accessions represented in TEA.db and found that the same accession was recovered as the top-ranked hit in all cases. The primary similarities ranged from 0.544 to 0.607, and the mean top1–top2 similarity margin was 0.272. This confirms internal recovery of represented accessions under the standardized workflow.

Second, we compared CamK-DB similarity profiles with genetic distances derived from the published Camellia phylogenetic tree. Because genetically close samples are expected to have higher CamK-DB similarity and lower phylogenetic distance, a negative correlation between similarity and distance supports the biological relevance of the database-derived similarity scores. The revised analysis showed that most samples displayed negative correlations between CamK-DB similarity and phylogenetic distance, indicating that the similarity scores are broadly consistent with phylogenetic relationships.

Third, we evaluated an out-of-database query sample, SRR11672521, which was not included in the standardized CamK-DB release. Although the exact accession was absent from the database, higher CamK-DB similarity was associated with lower phylogenetic distance to database accessions ($r = -0.37$, $P = 4.79 \times 10^{-15}$). This supports the use of CamK-DB as a nearest-neighbor fingerprinting system when exact matches are absent, while also clarifying that top hits should not be interpreted as definitive identities if the query accession or a close relative is not represented in the database.

Finally, we assessed group-level representation in the standardized 418-accession database. The retained accessions remained distributed across the major Camellia groups included in CamK-DB, with a median group size of 16, supporting the utility of the standardized database for broad group-level comparison. These additions have been incorporated into the revised Technical Validation, User Guide, and Discussion sections. Figure 2C, D, Lines 212-299, 405-417, and 602-615

Major Comment 3: “Limitations in Parentage Inference Methodology. The assessment of parentage inference is methodologically limited by the use of in silico mixed reads to simulate hybridization. While this approach effectively demonstrates the tool’s ability to deconvolve mixed genomic signals from two different sources, it does not accurately reflect true biological hybridization. Biological hybrids undergo meiotic recombination and segregation, resulting in a unique k-mer composition that differs significantly from a simple linear physical mixture of parental reads. The authors must clarify this distinction in the text and explicitly acknowledge that their current simulation primarily tests the detection of mixed samples rather than the inference of complex genealogical ancestry or hybrid origins.”

Response: We completely agree with the reviewer. We have revised this section throughout the manuscript. The experiment is no longer described as formal parentage inference or biological hybrid identification. Instead, it is now presented as a mixed-sample or dual-source signal detection test.

In the revised analysis, artificial mixed-read datasets were generated by combining reads from pairs of accessions represented in the standardized database. In all ten mixtures, the two highest-ranked database records corresponded to the two source accessions used to generate the query dataset. The primary similarity values ranged from 0.386 to 0.504, and the secondary similarity values ranged from 0.372 to 0.489. This demonstrates that CamK-DB can recover dual-source signals from artificial mixed-read data when both source accessions are present in the database.

We now explicitly state that physical mixing of reads from two accessions does not model true biological hybridization, because biological hybrids inherit recombined haplotypes following meiosis and segregation. Therefore, this analysis should be interpreted as mixed-sample signal detection, not as formal biological parentage or hybrid-origin inference. Formal hybrid-origin or parentage inference would require validated F1 progeny or inheritance-aware analyses. Lines 233-254, 418-424, and 608-615

Minor Comment 4: “Database Architecture Clarity (Figure 1). The description of the database architecture in Figure 1 requires significantly more detail to be transparent and reproducible. Specifically, the manuscript does not adequately explain the rationale behind partitioning the database into 13 distinct grouping tables, nor does it define the cryptic abbreviations used for these sub-tables. A clear mapping key or a more descriptive legend is necessary to help readers understand the logical organization and taxonomy of the stored data.”

Response: We have improved the explanation about this figure which is now Figure 3, and explained the information of retained 418 standardized accessions, the central tea_info metadata table, the group-specific fingerprint tables, and the relationship between metadata and MIKE-derived fingerprint records. We also added abbreviation key for all group-table names and explained the rationale for group-level partitioning. Lines 193-211, 447-451, and 577-582. Figure 1.

Minor Comment 5: “Quality of Visualizations (Figure 2). Figure 2 is currently too schematic and lacks the necessary technical depth to fully illustrate the study’s workflow. It fails to capture critical details of the MIKE algorithm, such as the hashing process or sketch generation, and the visual presentation is somewhat rudimentary for a high-impact publication. The authors should refine this figure to provide a more comprehensive and professionally polished representation of the computational pipeline that visually communicates the complexity of the methods used.”

Response: We agree and have completely redesigned Figure 2 which is Figure 1 now. The revised figure now presents the full MIKE-based fingerprint-generation workflow, including read preprocessing, 5× coverage standardization, KMC k-mer counting, k=21 setting, prefix/suffix grouping, MinHash-based sketching, .jac fingerprint generation, SQLite database construction, and Jaccard-based top-hit ranking. The figure has been redesigned with clearer panel structure, consistent terminology, and publication-quality formatting. Lines 106-172 and 557-565 , Figure 1

Cookie Preference Center

We use cookies which are necessary to make our site work. We may also use additional cookies to analyze, improve and personalize our content and your digital experience. For more information, see our Cookie Policy.

You may choose not to allow some types of cookies. However, blocking some types may impact your experience of our site and the services we are able to offer. See the different category headings below to find out more or change your settings.

Allow all

Manage Consent Preferences

Strictly Necessary Cookies

Always active

These cookies are necessary for the website to function and cannot be switched off in our systems. They are usually only set in response to actions made by you which amount to a request for services, such as setting your privacy preferences, logging in or filling in forms. You can set your browser to block or alert you about these cookies, but some parts of the site will not then work.

Functional Cookies

These cookies enable the website to provide enhanced functionality and personalisation. They may be set by us or by third party providers whose services we have added to our pages. If you do not allow these cookies then some or all of these services may not function properly.

Performance Cookies

These cookies allow us to count visits and traffic sources so we can measure and improve the performance of our site. They help us to know which pages are the most and least popular and see how visitors move around the site.

Targeting Cookies

These cookies may be set through our site by our advertising partners. They may be used by those companies to build a profile of your interests and show you relevant adverts on other sites. If you do not allow these cookies, you will experience less targeted advertising.

Cookie List

Clear

Apply Cancel

Consent Leg.Interest

Confirm my choices

Power
