# Supplementary material for: CamK-DB: A k-mer MinHash fingerprint database for reference-free genotyping of Camellia accessions
Source: Gigascience. 2026 Aug 21;15:giag088. doi: 10.1093/gigascience/giag088 (PMC13548848; doi:10.1093/gigascience/giag088)
Supplement: Supplementary file 2 [file giag088_giga-d-25-00529_original_submission.pdf]

GigaScience

CamK-DB: A k-mer MinHash fingerprint database for reference-free genotyping of Camellia accessions

--Manuscript Draft--

**Manuscript Number:**	GIGA-D-25-00529
**Full Title:**	CamK-DB: A k-mer MinHash fingerprint database for reference-free genotyping of Camellia accessions
**Article Type:**	Technical Note
**Funding Information:**	National Natural Science Foundation of China grant (32222019) — Prof. Xingtan Zhang<br>Shenzhen Science and Technology Program (RCYX20210706092103024) — Prof. Xingtan Zhang<br>Open Fund of Collaborative Innovation Center of Chinese Oolong Tea Industry (2025W 01) — Prof. Xingtan Zhang<br>Project of Science and Technology Plan of Nanping city (N2024Z007) — Prof. Xingtan Zhang<br>Project of Science and Technology Plan of Fujian Province (2023N5013) — Prof. Xingtan Zhang<br>Project of Science and Technology Plan of Fujian Province (2024N3013) — Prof. Xingtan Zhang<br>Scientific Research Fund of Wuyi University (2018N2004) — Prof. Xingtan Zhang<br>Scientific Research Fund of Wuyi University (2021L3058) — Prof. Xingtan Zhang<br>Special Funds for Colleges and Universities in Fujian Province to Improve the Level of Running Schools (No.77 [2022]) — Prof. Xingtan Zhang
**Abstract:**	Tea (Camellia sinensis L.), a major global economic crop in Asia, poses challenges for genetic identification because its highly heterozygous, repetitive genome reduces the efficacy of conventional single-nucleotide polymorphism (SNP) and microsatellite markers. Interspecific hybridization further blurs genetic boundaries, promotes adulteration, and compromises breeding programs and commercial supply chains. To address these issues, we developed CamK-DB, a DNA fingerprinting database employing the MinHash-based k-mer algorithm (MIKE). The repository integrates genomic signatures from 735 tea accessions spanning diverse geographical origins and phenotypic characteristics, derived from whole-genome resequencing data. Each accession is represented by compact MinHash fingerprints (k-mer size = 21, sketch size = 1000 hash values per genome) stored in a standardized SQLite relational database with associated taxonomic metadata. CamK-DB provides a command-line interface for data management and a custom C++ query engine that computes top-10 matches using Jaccard similarity, complemented by a QT-based graphical interface for interactive analysis. This resource offers a robust and scalable framework for precise and routine germplasm identification, genomic phylogenetic inference, and strategic breeding program design.
**Corresponding Author:**	Xingtan Zhang<br>Chinese Academy of Agricultural Sciences Agricultural Genomes Institute at Shenzhen<br>Shenzhen, --- Select One --- CHINA
**Corresponding Author Secondary Information:**	
**Corresponding Author's Institution:**	Chinese Academy of Agricultural Sciences Agricultural Genomes Institute at Shenzhen

Powered by Editorial Manager® and ProduXion Manager® from Aries Systems Corporation

Corresponding Author's Secondary Institution:	
First Author:	Feiquan Wang
First Author Secondary Information:	
Order of Authors:	Feiquan Wang
	Noor ul Ain, Ph.D.
	Fang Wang, Ph.D.
	Shengcheng Zhang, Ph.D.
	Weilong Kong, Ph.D.
	Yutao Shi, Ph.D.
	Hua Feng, Ph.D.
	Bo Zhang, Ph.D.
	Xingtan Zhang, Ph.D.
Order of Authors Secondary Information:	
Additional Information:	
Question	Response
Are you submitting this manuscript to a special series or article collection?	No
**Experimental design and statistics**<br><br>Full details of the experimental design and statistical methods used should be given in the Methods section, as detailed in our Minimum Standards Reporting Checklist. Information essential to interpreting the data presented should be made available in the figure legends.<br><br>Have you included all the information requested in your manuscript?	No
If not, please give reasons for any omissions below.<br><br>as follow-up to "**Experimental design and statistics**<br><br>Full details of the experimental design and statistical methods used should be given in the Methods section, as detailed in our Minimum Standards Reporting Checklist. Information essential to interpreting the	This submission is a Technical Note describing a computational/database resource and its validation. There were no new wet-lab experiments, interventions, randomization, blinding, or health-related outcomes, and no hypothesis-testing statistics (e.g., p-values/power calculations) were required for the aims of this work. All applicable methodological details are provided in the Methods and figure legends, including data sources, preprocessing steps, algorithm parameters (e.g., k-mer size/sketch size), similarity metric, and the validation/benchmarking procedure. Items from the Minimum Standards Reporting Checklist that relate to experimental interventions or inferential statistical testing are not applicable to this study.

data presented should be made available in the figure legends.<br><br>Have you included all the information requested in your manuscript?<br><br>"	
**Resources**<br><br>A description of all resources used, including antibodies, cell lines, animals and software tools, with enough information to allow them to be uniquely identified, should be included in the Methods section. Authors are strongly encouraged to cite Research Resource Identifiers (RRIDs) for antibodies, model organisms and tools, where possible.<br><br>Have you included the information requested as detailed in our Minimum Standards Reporting Checklist?	Yes
**Availability of data and materials**<br><br>All datasets and code on which the conclusions of the paper rely must be either included in your submission or deposited in publicly available repositories (where available and ethically appropriate), referencing such data using a unique identifier in the references and in the "Availability of Data and Materials" section of your manuscript.<br><br>Have you have met the above requirement as detailed in our Minimum Standards Reporting Checklist?	Yes
GigaScience has policies and guidelines in place for the use of generative AI-writing tools such as ChatGPT. If you have used such writing tools to assist with writing the manuscript this must be declared and cited in the text. Authors should not list AI-writing tools and other AI-assisted technologies as an author or	Yes

co-author and should acknowledge that they are fully responsible for text generated or refined by AI-writing tools.<p><br><br>A summary of use (particularly in the introduction or among methods) needs to be included at the end of the paper, and the outputs should also be included as a supplementary file hosted in GigaDB or other open repositories. Please read our guidelines for more information. <p><br><br>By submitting to GigaScience, you are aware of the journal's AI-writing tools policy, and if you have declared use of such tools below, you have acknowledged this where appropriate in your manuscript and have made a summary of use and outputs available. <p><br><b>AI-assisted writing tools have been used in the preparation of this manuscript?	

 Click here to access/download;LaTeX - Other (clo, cls, sty etc.);data links.docx

We have uploaded TEA.db file for CamK-DB, and the main program to zenodo, the doi is: https://doi.org/10.5281/zenodo.15588935

Data availability

The raw sequences data in this paper are publicly available in the GSA database (https://bigd.big.ac.cn/gsa/, BioProject, accession PRJCA026608 (Kong et al., 2025)). The other data generated in the previous studies are publicly available in the National Center for Biotechnology Information database under the accessions PRJNA665594 (Zhang et al., 2021) and PRJCA011312 (Lei et al., 2022), and in the GSA database under the accessions PRJCA003090 (Zhang et al., 2021), PRJNA646044 (Wang et al., 2020), PRJNA716079 (Lu et al., 2021),and PRJNA597714(Xia et al., 2020).

Kong, W., Kong, X., Xia, Z., Li, Xiaofeng, Wang, F., Shan, R., Chen, Z., You, X., Zhao, Y., Hu, Y., Zheng, S., Zhong, S., Zhang, S., Zhang, Yanbing, Fang, K., Wang, Y., Liu, H., Zhang, Yazhen, Li, Xinlei, Wu, H., Chen, G.B., Zhang, X., Chen, C., 2025. Genomic analysis of 1,325 Camellia accessions sheds light on agronomic and metabolic traits for tea plant improvement. Nat Genet 57, 997–1007. https://doi.org/10.1038/S41588-025-02135-Z;SUBJMETA=208,2491,449,457,631;KWRD=PLANT+GENETICS,POPULATION+GENETICS

Lei, Y., Yang, L., Duan, S., Ning, S., Li, D., Wang, Z., Xiang, G., Yang, L., Wang, C., Zhang, Shiyu, Zhang, Shuangyan, Ye, S., Kui, L., Singh, P., Sheng, J., Dong, Y., 2022. Whole-genome resequencing reveals the origin of tea in Lincang. Front Plant Sci 13, 984422. https://doi.org/10.3389/FPLS.2022.984422/BIBTEX

Lu, L., Chen, H., Wang, X., Zhao, Y., Yao, X., Xiong, B., Deng, Y., Zhao, D., 2021. Genome-level diversification of eight ancient tea populations in the Guizhou and Yunnan regions identifies candidate genes for core agronomic traits. Hortic Res 8, 1–10. https://doi.org/10.1038/S41438-021-00617-9;SUBJMETA=208,2491,449,631,726;KWRD=GENOME,PLANT+GENETICS

Wang, Xinchao, Feng, H., Chang, Y., Ma, C., Wang, Liyuan, Hao, X., Li, A., Cheng, H., Wang, Lu, Cui, P., Jin, J., Wang, Xiaobo, Wei, K., Ai, C., Zhao, S., Wu, Z., Li, Y., Liu, B., Wang, G.D., Chen, L., Ruan, J., Yang, Y., 2020. Population sequencing enhances understanding of tea plant evolution. Nature Communications 2020 11:1 11, 1–10. https://doi.org/10.1038/s41467-020-18228-8

Xia, E., Tong, W., Hou, Y., An, Y., Chen, L., Wu, Q., Liu, Y., Yu, J., Li, F., Li, R., Li, P., Zhao, H., Ge, R., Huang, J., Mallano, A.I., Zhang, Y., Liu, S., Deng, W., Song, C., Zhang, Zhaoliang, Zhao, J., Wei, S., Zhang, Zhengzhu, Xia, T., Wei, C., Wan, X., 2020. The Reference Genome of Tea Plant and Resequencing of 81 Diverse Accessions Provide Insights into Its Genome Evolution and Adaptation. Mol Plant 13, 1013–1026.

https://doi.org/10.1016/J.MOLP.2020.04.010

Zhang, X., Chen, S., Shi, L., Gong, D., Zhang, S., Zhao, Q., Zhan, D., Vasseur, L., Wang, Y., Yu, J., Liao, Z., Xu, X., Qi, R., Wang, W., Ma, Yunran, Wang, P., Ye, N., Ma, D., Shi, Y., Wang, H., Ma, X., Kong, X., Lin, J., Wei, L., Ma, Yaying, Li, R., Hu, G., He, H., Zhang, L., Ming, R., Wang, G., Tang, H., You, M., 2021. Haplotype-resolved genome assembly provides insights into evolutionary history of the tea plant Camellia sinensis. Nat Genet 53, 1250–1259. https://doi.org/10.1038/S41588-021-00895-Y;SUBJMETA=208,212,2491,449,457,631;KWRD=GENOMICS,PLANT+GENETICS,POPULATION+GENETICS

Manuscript Click here to access/download;Manuscript;Technical Note.docx

CamK-DB: A *k*-mer MinHash fingerprint database for reference-free genotyping of *Camellia* accessions

Feiquan Wang[1,#], Noor-ul-Áin[2,#], Fang Wang[2,#], Shengcheng Zhang[2,#], Weilong Kong[2], Yutao Shi[1], Hua Feng[1], Bo Zhang[1,*], Xingtan Zhang[2,*]

[1] College of Tea and Food Sciences, Collaborative Innovation Center of Chinese Oolong Tea Industry, Wuyi University, Tea Engineering Research Center of Fujian Higher Education, Wuyishan Fujian, 354300, China

[2] State Key Laboratory of Tropical Crop Breeding, Shenzhen Branch, Guangdong Laboratory for Lingnan Modern Agriculture, Genome Analysis Laboratory of the Ministry of Agriculture and Rural Affairs, Agricultural Genomics Institute at Shenzhen, Chinese Academy of Agricultural Sciences, Shenzhen, 518120, China

# These authors contributed equally

* Correspondance to Xingtan Zhang (zhangxingtan@caas.cn) and Bo Zhang (Zhangbo@wuyiu.edu.cn)

Abstract

Tea (*Camellia sinensis* L.), a major global economic crop in Asia, poses challenges for genetic identification because its highly heterozygous, repetitive genome reduces the efficacy of conventional single-nucleotide polymorphism (SNP) and microsatellite markers. Interspecific hybridization further blurs genetic boundaries, promotes adulteration, and compromises breeding programs and commercial supply chains. To address these issues, we developed Cam*K*-DB, a DNA fingerprinting database employing the MinHash-based *k*-mer algorithm (MIKE). The repository integrates genomic signatures from 735 tea accessions spanning diverse geographical origins and phenotypic characteristics, derived from whole-genome resequencing data. Each accession is represented by compact MinHash fingerprints (*k*-mer size = 21, sketch size = 1000 hash values per genome) stored in a standardized SQLite relational database with associated taxonomic metadata. Cam*K*-DB provides a command-line interface for data management and a custom C++ query engine that computes top-10 matches using Jaccard similarity, complemented by a QT-based graphical interface for interactive analysis. This resource offers a robust and scalable framework for precise and routine germplasm identification, genomic phylogenetic inference, and strategic breeding program design.

Availability and implementation: Cam*K*-DB (database and code) is publicly available at (https://github.com/sc-zhang/CamK-DB). Cam*K*-DB binaries are provided for Windows 10/11 and Linux (x86_64, glibc ≥ 2.27)

Keywords: MinHash; *k*-mer, *Camellia sinensis*, alignment-free genomics, Jaccard similarity, germplasm identification, SQLite database

Findings

Background

Tea plants (*Camellia sinensis*), famous for their outstanding foliage, are among the world's most cherished non-alcoholic beverages. Beyond its scope as a beverage crop, the tea industry extends to different sectors, such as finished products, tea ware, and global expansions. Technically, *C. sinensis* (L.) is the principal cultivated species for tea production, sectioned as *C. sinensis* var. *sinensis* (small-leaf tea) and *C. sinensis* var. *assamica* (large-leaf tea). Additionally, several wild relatives, including *C. taliensis*, *C. irrawadiensis*, *C. tachangensis*, and *C. grandibracteata*, account for the genetic diversity in China. Tea leaves face many challenges in phenotypic characterization due to their dense foliage, architecture, and phenotypic similarity. Additionally, tea plants exhibit complex genomic features, including high heterozygosity (>2%), frequent interspecific hybridization, extensive vegetative propagation, and a genome dominated by repetitive sequences such as long terminal repeat (LTR) retrotransposons (approximately 70% of the genome)[1]. These characteristics complicate traditional genetic analysis and cultivar identification. Thus, mislabeling and fraudulent substitution of tea varieties undermine consumer trust and economic stability, necessitating a robust anti-counterfeiting measure for the rapid and accurate identification of tea varieties.

Current methods for tea variety identification, such as morphological analysis and chemical profiling, are often time-consuming and lack the precision to distinguish closely related species and varieties [2,3]. Conventional methods of DNA fingerprinting relied on molecular markers (e.g., SSRs/microsatellites, AFLPs), allowing cultivar identification and population genetics, but showed limited genome-wide resolution. Reduced representation approaches such as RAD-seq and genotyping-by-sequencing (GBS) based on short reads matured into alignment-based pipelines that infer SNPs against a reference genome [4,5]. Molecular markers, such as Simple Sequence Repeats (SSRs) and Single Nucleotide Polymorphisms (SNPs), are accurate and

efficient but pose high costs and complexity in genotyping [2,3]. These methods benefited from multiple-sequence-alignment tools (e.g., BLAST, ClustalW) that remain foundational and widely used [6]. Recently, *k*-mer-based analytical approaches have been extensively utilized in computational biology; however, the determination of an optimal *k*-mer length remains contingent upon heuristic optimization methodologies. Several hashing-based techniques, such as MinHash, Bloom filter, and Count-Min Sketch [7], have been developed or adopted to efficiently compute *k*-mer-based similarity methods. MinHash efficiently estimates genetic similarity by representing genomes as compact sketches, thereby significantly reducing the computational demands. Inspired by these advancements, we previously developed the MinHash-based *k*-mer algorithm MIKE, tailored to process extensive genomic data, generate compact genetic signatures, and enable rapid similarity calculations [8].

Tea genomics has recently been endowed with two chromosome-scale assemblies of *C. lanceoleosa* and wild oil-Camellia and a haplotype-resolved tetraploid *C. oleifera* genome, providing valuable insights into genome evolution, population structure, self-incompatibility, and agronomic traits [9–12]. Owing to the availability of large-scale genomic datasets, there is a dire need for a centralized and standardized repository that can facilitate the identification of different accessions and varieties irrespective of phenotypic and chemical methods. Many recent studies have contributed to the compilation of valuable datasets in various aspects of tea, such as an atlas of genomic variation, metabolites of tea cultivars, or aspects of tea plant biology [13,14]. Despite these recent advances, a publicly available *k*-mer fingerprint database for identifying tea accessions is still lacking, and Cam*K*-DB has been developed to address this gap. This alignment-free approach compares samples directly via their *k*-mer content, avoiding read mapping and multiple alignment. MinHash sketches transform large datasets into compact "signatures," enabling rapid, accurate similarity queries with small storage footprints. As it helps in (i) reference-free operation, (ii) good performance at low-to-moderate coverage, (iii) fast search across many accessions, and (iv) easy sharing/indexing of compact sketches. Benefitting from this, we created a *k*-mer based, low-cost, user-friendly database for germplasm identification. The base dataset is a spatially diverse resource of 735 accessions of different species of *Camellia*. Built upon QT architecture and SQLite, the database enables rapid identification of tea tree varieties, offering unprecedented accuracy and efficiency in cultivar authentication, hybrid detection, and evolutionary research.

Theory behind Database Construction

Database construction was based on two key elements.

1. DNA Markers (*k*-mers): The foundation of the database lies in the genetic information obtained from 735 accessions of tea resequencing data. We used an alignment-free and more accurate approach, *k*-mer similarity, by creating substrings of length k = 21 in MIKE. We used the algorithm's default and recommended setting k=21 to generate the fingerprint. Real *k*-mers, which are short DNA sequences of length *k* that appear in a single location (e.g., at a single genomic position, in a single species from a larger set of species), are called "*unique k-mers*" [15]. We then created MinHash fingerprints of these unique k-mers by generating prefixes and suffixes and creating a one-hot vector. The process was extended to create an index of nonzero elements, followed by a permutation to reduce dimensionality and estimate genetic similarity.

2. Jaccard Similarity (MinHash Signatures): MIKE harnessed the *MinHash* algorithm to create a compact and exclusive MinHash signature for each biological sample of tea. These MinHash fingerprints were processed to calculate the Jaccard coefficient, which is the ratio of the count of shared to unique *k*-mers between two samples. MIKE used this approximate *Jaccard coefficient* to calculate the evolutionary distance (d) between samples using a formula derived from the Poisson distribution, assuming single-base substitutions (Figure 1).

   The Jaccard Similarity is calculated with the formula below:

$$Jacard\ Similarity = \frac{|S_1 \cap S_2|}{|S_1 \cup S_2|}$$

   Where S_1 denoted the *k*-mers set from sample 1, while S2 denoted the *k*-mers set from sample2.

Processing reads in MIKE

MIKE was developed to estimate sequence similarity based on *k*-mer frequency. We employed MIKE to enumerate all possible *k*-mers within a 1000-sketch size and then calculated the frequency of each *k*-mer. MinHash fingerprints were then derived from the *k*-mer representations of the lowest hash values. This signature fingerprint serves as a heuristic representation of a sequence, capturing its essential features. The resulting ".jac" files contained integer–count pairs encoding MinHash signatures, specific to each

sample's signature, comprising up to 1,048,576 hash buckets to capture both the presence and frequency of hashed genomic elements (Figure 2).

Construction of the database, GUI, and basic features

Following data collection, the database schema was constructed using an in-house Python script (create_db.py), which processed two primary inputs: a tab-delimited metadata file containing sample identifiers (ID), nomenclature (Chinese and English names), taxonomic classification (species and group), geographic provenance (country and region), and a directory of". jac" files, each corresponding to a unique sample ID. The script generated an SQLite database file (Cam*K*-DB) designed to facilitate efficient fingerprint data retrieval through an SQLite-compatible graphical user interface (GUI). The schema featured a central tea_info table for storing dataset metadata, alongside individual tables for each sample organized by ID, which housed MinHash signatures and precomputed similarity strings. This architecture minimized reliance on cross-table joins during query filtering, thereby enhancing the computational efficiency for per-sample analytical operations, while maintaining structural clarity and scalability for downstream applications.

The core data of Cam*K*-DB (~3.9 GB, (3,901,136,896 bytes)) consists of MinHash fingerprints derived from unique *k*-mers using Mike-generated ".jac" files that encode MinHash signatures as integer–count pairs. Each sample's signature includes up to 1,048,576 hash buckets, capturing the presence and frequency of the hashed elements. This approach enables the efficient representation and comparison of genomic features through MinHash-based similarity estimation. This database was designed for efficient storage and retrieval. It uses a master table (tea_info) containing general information about each tea tree sample (ID, name, classification, and origin). To reduce storage space and improve query speed, the database was divided into 14 sub-tables (one master table and 13 grouping tables), each storing the MinHash signatures for a specific group of tea trees. This allows faster searches within particular groups. The master table and sub-tables were linked using a tea tree ID as a foreign key. This database design was optimized for fast searching and comparison of the MinHash signatures (Figure 3).

Data Records

Cam*K*-DB is distributed as a single SQLite database file (TEA.db), which stores all derived MinHash fingerprints and sample-level metadata for the 735 Camellia accessions. The central table, tea_info, contains one row per accession and includes

fields such as sample ID, cultivar names, species, variety or group, country and region of origin, and other basic metadata. For each logical group (for example species, region, or other user-defined subset), an additional table was created with three fields: id, name, and similarity. The id field is the primary key (PK) of the group table and, at the same time, acts as a foreign key referencing the corresponding accession in tea_info. The similarity field stores the MinHash *k*-mer sketch generated by MIKE and is used to compute the Jaccard's similarity between each stored sample and a query. The TEA.db file, together with the complete Cam*K*-DB source code, sample data, and GUI resources, is archived at Zenodo (DOI: 10.5281/zenodo.15588935).

Technical Validation

To assess the self-identification performance of Cam*K*-DB, we randomly selected seven accessions (C5A, ZH-1A, SRR12322236, SRR12322243, SRR12333999, SRR12337253, and SRR12337254) that were already present in the database. For each accession, we downloaded the resequencing data and constructed the corresponding ".jac" file. When these files were used as queries against Cam*K*-DB, the primary hit was always the same accession as the query sample, with Jaccard similarity values ranging from 0.961 to 0.998 (mean = 0.975; Table 1). Contrary, the secondary matches showed markedly lower similarity, ranging from 0.295 to 0.605 (mean = 0.389). These results show that Cam*K*-DB can distinguish identical from non-identical samples and can be used to determine whether a newly sequenced sample is already contained in the database. Table 1.

To validate whether Cam*K*-DB can be used to identify parental accessions of a sample when both parents are contained in Cam*K*-DB, we randomly selected 5 accessions from Cam*K*-DB, and down-sampled their resequencing data to 10x coverage. For every pair, we combined their down-sampled reads to generate mixed two-sample resequencing datasets and constructed the ".jac" file. The two database records with the highest similarity scores always corresponded to the two constituent parental accessions (Table 2). Across all ten two-sample mixtures, the primary similarity values ranged from 0.509 to 0.578 (mean ≈ 0.532), and the corresponding secondary similarity values ranged from 0.488 to 0.554 (mean ≈ 0.510). These results indicate that Cam*K*-DB can be used to trace recent ancestry and to infer parental accessions of a sample, provided that the parental lines are represented in the database.

Methods

Source resequencing datasets and licensing

Cam*K*-DB integrates whole-genome resequencing data from 735 *Camellia* accessions. Newly generated data for 320 accessions were produced as part of a recent large-scale genomic survey of Camellia germplasm and are deposited in the GSA database (https://bigd.big.ac.cn/gsa/, BioProject, accession PRJCA026608 [12]). Previously published resequencing data for 415 accessions were downloaded from the GSA and NCBI SRA repositories under BioProjects PRJNA665594 [16] and PRJCA011312 [17], and in the GSA database under the accession PRJCA003090 [16], PRJNA646044 [18], PRJNA716079 [19], and PRJNA597714 [20]. All of these datasets were obtained from repositories that explicitly permit reuse for research purposes. In Cam*K*-DB, we only redistribute derived MinHash fingerprints, the SQLite database file, and sample-level metadata; we do not redistribute the original raw reads from third-party studies, thereby complying with the terms and conditions of the original data providers.

Sequencing data processing

For the construction of database, this study utilized a large-scale dataset of 735 *Camellia* accessions, based on our recently published research on *Camellia's* evolutionary history. (DOI: 10.1038/s41588-025-02135-z) [21]. This publication encompassed an expanded dataset that included 802 newly sequenced reads and 523 previously sequenced reads. *Camellia* accessions originated from 13 countries: Azerbaijan, China, Georgia, Iran, Japan, Kenya, Laos, Myanmar, Nepal, Russia, Sri Lanka, the USA, and Vietnam. However, we used 320 newly sequenced accessions and 415 publicly available accessions (total n = 735) after QC for Cam*K*-DB. The dataset included diverse *Camellia* species: *C. oleifera*, *C. quinquelocularis*, *C. sasanqua*, *C. sinensis* var. *assamica*, *C. sinensis* var. *pubilimba*, *C. sinensis* var. *sinensis*, *C. tachangensis* var. *remotiserrata*, and *C. taliensis*. Importantly, the *C. sinensis* accessions represent a mix of wild, elite, and landrace varieties, whereas the remaining other species were included as wild samples. Among 735 selected accessions, we used 320 newly sequenced *Camellia* accessions from multiple countries, including Azerbaijan, China, Georgia, Iran, Laos, Myanmar, Sri Lanka, and Russia, from our sequenced resource (sequencing depth ranging between 9-23) (Table S1). Genomic DNA from the leaves was extracted using the DNeasy Plant Mini Kit (Qiagen), followed by 5 µg to construct libraries with an insert size of 500 bp. Each sample consisted of 150 bp paired-end reads on the DNBSEQ-T7 platform (Beijing Genomics Institute). Furthermore, to increase the sample diversity, 415 deep resequencing data with more than 8× sequencing depth were downloaded from public *Camellia* accessions (National Genomics Data Center, https://ngdc.cncb.ac.cn/) and (National Center for Biotechnology Information, https://www.ncbi.nlm.nih.gov/) from the given research [16–20]. Raw sequencing reads were subjected to quality control and removal of low-quality reads and adapter sequences by Trimmomatic with default parameters i.e., Trimmomatic PE -phred33 ILLUMINACLIP:adapters.fa:2:30:10 LEADING:3 TRAILING:3 SLIDINGWINDOW:4:15

MINLEN:50 [22]. The resulting high-quality reads were processed using the MIKE tool to generate minimum hash signatures for each sample (Table S1). Quality assessment revealed high read quality, with Q20 and Q30 scores averaging 98.0% and 93.5%, respectively. The average GC content was approximately 40.1%, which was consistent with the expected GC content of *C. sinensis* genomes. The number of high-quality reads per sample ranged from ~205 million to ~580 million, yielding a total base count per sample ranging from ~30 Gb to ~87 Gb. Following adapter trimming and quality filtering, the high-quality reads were aligned to the chromosomal-scale *C. sinensis* TGY genome (~3.15 Gb) [23] by BWA [24] with default parameters. The alignment results demonstrated high sequence similarity and minimal contamination, with an average genomic mapping rate of 99.5%. Genome coverage was uniformly high across all samples, exceeding 88% on average, with several samples achieving a genome anchorage greater than 94% (Table S1).

User guide

A dual-interface system comprising both a graphical user interface (GUI) and a command-line interface (CLI) was developed to enable flexible, efficient querying of the Cam*K*-DB database. CLI, accessible via the syntax /path/to/query_db Cam*K*-DBsample.jac [pre_cnt], complements GUI by enabling high-throughput scriptable database interactions. Here, Cam*K*-DB specifies the target database, sample.jac denotes the query fingerprint, and the optional [pre_cnt] parameter constrains the number of hash buckets compared, thereby reducing the computational overhead for preliminary analyses. Underlying both interfaces is the query_db module, which employs three core functions to optimize the similarity calculations. A max-heap-based algorithm dynamically retains the top 10 most identical samples during database scans, thus minimizing the sorting costs.

The GUI, built using the QT framework, provides an intuitive platform for users to query the database by name, taxonomic classification, geographic region, or sequencing data (in the FASTQ format) to identify unknown tea tree varieties and their closest genetic relatives. Query results are displayed in a sortable table, detailing key attributes, such as tea tree name (Chinese and English), species, taxonomic group, geographic origin, and similarity score. To streamline computational workflows, the GUI automates database detection, prompting users to locate or regenerate the db/Cam*K*-Db file if missing. Query execution begins by selecting an ".jac" fingerprint file via the "Select" button, followed by parallelized processing initiated by the "Query" button. During the analysis, user inputs are temporarily disabled, and a frameless model loading dialog provides visual feedback, ensuring a simplistic and user-friendly experience (Figure 2).

Supplementary data

Supplementary Table S1. List of all tea accessions, quality metrics, and their related information used in database construction.

Author Contributions

Conceptualization: X.Z., F.W. and B.Z.; Methodology and algorithm development: F.W. and Noor; Database and GUI development: S.Z.; Validation and analysis: W.K., Y.S. and H.F.; Writing: Noor and F.W: Author B; Writing-review & editing: F.W. and X.Z.

Data availability

The CamK-DB dataset, including the TEA.db SQLite database, per-sample MinHash fingerprint files and the main program, is archived on Zenodo (DOI: 10.5281/zenodo.15588935).

Newly generated raw whole-genome resequencing data are publicly available in the GSA database (https://bigd.big.ac.cn/gsa/) under BioProject accession PRJCA026608. Previously published resequencing data used to construct Cam*K*-DB can be accessed under BioProjects PRJNA665594, PRJCA011312, PRJCA003090, PRJNA646044, PRJNA716079 and PRJNA597714 in GSA and NCBI SRA.

Code Availability

All software and analytical scripts developed for this study are publicly available under open-source licenses via GitHub repositories:

-MIKE toolkit:

(https://github.com/Argonum-Clever2/mike)

Cam*K*-DB database and GUI:

(https://github.com/sc-zhang/CamK-DB)

Data access note

We have uploaded a database file "TEA.db" for Cam*K*-DB and the main program to zenodo, the doi is:

https://doi.org/10.5281/zenodo.15588935

Competing Interests

The authors declare no competing interests.

Funding Information

This work was supported by National Natural Science Foundation of China grant (No. 32222019 to X.Z.) and Shenzhen Science and Technology Program (Grant No. RCYX20210706092103024 to X.Z.), the Open Fund of Collaborative Innovation Center of Chinese Oolong Tea Industry (2025W 01), the Project of Science and Technology Plan of Nanping city (N2024Z007), the Project of Science and Technology Plan of Fujian Province (2023N5013, 2024N3013), the Scientific Research Fund of Wuyi University (2018N2004, 2021L3058), the Special Funds for Colleges and Universities in Fujian Province to Improve the Level of Running Schools (No.77 [2022]).

References

1. Zhang, Z. B. *et al.* Understanding the Origin and Evolution of Tea (Camellia sinensis [L.]): Genomic Advances in Tea. *J Mol Evol* 91, 156–168 (2023).
2. Shen, Y. *et al.* Development of Genome-Wide Intron Length Polymorphism (ILP) Markers in Tea Plant (Camellia sinensis) and Related Applications for Genetics Research. *Int J Mol Sci* 25, 3241 (2024).
3. Wang, X., Xin, B., Guo, M., Yu, G. & Wang, J. GWASTool: A web pipeline for detecting SNP-phenotype associations. *Fundamental Research* 4, 761–769 (2024).
4. Baird, N. A. *et al.* Rapid SNP Discovery and Genetic Mapping Using Sequenced RAD Markers. *PLoS One* 3, e3376 (2008).
5. Elshire, R. J. *et al.* A Robust, Simple Genotyping-by-Sequencing (GBS) Approach for High Diversity Species. *PLoS One* 6, e19379 (2011).
6. Boratyn, G. M. *et al.* BLAST: a more efficient report with usability improvements. *Nucleic Acids Res* 41, W29–W33 (2013).
7. Liu, S. & Koslicki, D. CMash: fast, multi-resolution estimation of k-mer-based Jaccard and containment indices. *Bioinformatics* 38, i28–i35 (2022).
8. Wang, F. *et al.* MIKE: an ultrafast, assembly-, and alignment-free approach for phylogenetic tree construction. *Bioinformatics* 40, (2024).
9. Lin, P. *et al.* The genome of oil-Camellia and population genomics analysis provide insights into seed oil domestication. *Genome Biol* 23, 1–21 (2022).
10. Zhang, L. *et al.* The tetraploid Camellia oleifera genome provides insights into evolution, agronomic traits, and genetic architecture of oil Camellia plants. *Cell Rep* 43, (2024).
11. Gong, W. *et al.* Chromosome-level genome of Camellia lanceoleosa provides a valuable resource for understanding genome evolution and self-incompatibility. *Plant Journal* 110, 881–898 (2022).

12. Kong, W. *et al.* Genomic analysis of 1,325 Camellia accessions sheds light on agronomic and metabolic traits for tea plant improvement. *Nat Genet* 57, 997–1007 (2025).

13. An, Y., Zhang, X., Jiang, S., Zhao, J. & Zhang, F. TeaPVs: a comprehensive genomic variation database for tea plant (Camellia sinensis). *BMC Plant Biol* 22, 1–7 (2022).

14. Chen, J. D. *et al.* TeaGVD: A comprehensive database of genomic variations for uncovering the genetic architecture of metabolic traits in tea plants. *Front Plant Sci* 13, (2022).

15. Zentgraf, J. & Rahmann, S. Swiftly Identifying Strongly Unique k-Mers. in *Leibniz International Proceedings in Informatics, LIPIcs* vol. 312 (Schloss Dagstuhl- Leibniz-Zentrum fur Informatik GmbH, Dagstuhl Publishing, 2024).

16. Zhang, X. *et al.* Haplotype-resolved genome assembly provides insights into evolutionary history of the tea plant Camellia sinensis. *Nat Genet* 53, 1250–1259 (2021).

17. Lei, Y. *et al.* Whole-genome resequencing reveals the origin of tea in Lincang. *Front Plant Sci* 13, 984422 (2022).

18. Wang, X. *et al.* Population sequencing enhances understanding of tea plant evolution. *Nature Communications 2020 11:1* 11, 1–10 (2020).

19. Lu, L. *et al.* Genome-level diversification of eight ancient tea populations in the Guizhou and Yunnan regions identifies candidate genes for core agronomic traits. *Hortic Res* 8, 1–10 (2021).

20. Xia, E. *et al.* The Reference Genome of Tea Plant and Resequencing of 81 Diverse Accessions Provide Insights into Its Genome Evolution and Adaptation. *Mol Plant* 13, 1013–1026 (2020).

21. Kong, W. *et al.* Genomic analysis of 1,325 Camellia accessions sheds light on agronomic and metabolic traits for tea plant improvement. *Nat Genet* https://doi.org/10.1038/s41588-025-02135-z (2025) doi:10.1038/s41588-025-02135-z.

22. Bolger, A. M., Lohse, M. & Usadel, B. Trimmomatic: a flexible trimmer for Illumina sequence data. *Bioinformatics* 30, 2114–2120 (2014).

23. Zhang, X. *et al.* Haplotype-resolved genome assembly provides insights into evolutionary history of the tea plant Camellia sinensis. *Nature Genetics 2021 53:8* 53, 1250–1259 (2021).

24. Li, H. & Durbin, R. Fast and accurate short read alignment with Burrows–Wheeler transform. *Bioinformatics* 25, 1754–1760 (2009).

Figure Captions

Figure 1. The relationships among all tables are organized around the central tea_info table, which stores metadata for every sample. For each group (e.g. species, region or other logical subset), an additional table was created with three fields: id, name and similarity. The id field

is the primary key (PK) of the group table and simultaneously acts as a foreign key referencing the corresponding sample in tea_info. The similarity field stores the MinHash *k*-mer sketch produced by MIKE, which is used to compute Jaccard similarity between each stored sample and a query.

Figure 2. Regional information of the dataset and framework for the construction and implementation of the database.

Figure 3. Cam*K*-DB graphical user interface (GUI) workflow for varietal identification (steps 1-6). Pictures are arranged from top-to-bottom. Step 1 - Choose query input. Select either a local FASTQ/FASTA file or an existing sample from the database, designated as the query. Step 2 automates database detection or prompts for creating "Tea.db" file and GUI interface provides a selection bar for the following step. Step 3- Open ".minhash.jac "file for processing. Step 5 - Run similarity search and inspect results. Launch the search to obtain nearest neighbors. Step 6 - The results table shows each candidate's sample ID, name, and Jaccard similarity (higher is closer).

Table1. Query result of samples contained in the database

Sample	***Primary match***		***Secondary match***	
	**ID**	**similarity**	**ID**	**similarity**
C5A	C5A	0.997897	C3A	0.338155
ZH-1A	ZH-1A	0.992727	ZH-4A	0.364367
SRR12322236	SRR12322236	0.968909	SRR11672538	0.605417
SRR12322243	SRR12322243	0.968369	SRR12265472	0.366153
SRR12333999	SRR12333999	0.965834	SRR12329554	0.35455
SRR12337253	SRR12337253	0.960978	SRR12322233	0.295263
SRR12337254	SRR12337254	0.96791	SRR12328812	0.401559

Table2. Query results for two-sample mixed resequencing data

Sample1	***Sample2***	***Primary match***		***Secondary match***	
		**ID**	**similarity**	**ID**	**similarity**
SRR12322236	**SRR12322243**	SRR12322243	0.578255	SRR12322236	0.553881
SRR12322236	**SRR12333999**	SRR12333999	0.526315	SRR12322236	0.488366
SRR12322236	**SRR12337253**	SRR12337253	0.537477	SRR12322236	0.492161

SRR12322236	**SRR12337254**	SRR12337254	0.549704	SRR12322236	0.513578
SRR12322243	**SRR12333999**	SRR12333999	0.516018	SRR12322243	0.505456
SRR12322243	**SRR12337253**	SRR12337253	0.531123	SRR12322243	0.506955
SRR12322243	**SRR12337254**	SRR12337254	0.538876	SRR12322243	0.525018
SRR12333999	**SRR12337253**	SRR12337253	0.509444	SRR12333999	0.501213
SRR12333999	**SRR12337254**	SRR12333999	0.511309	SRR12337254	0.508364
SRR12337253	**SRR12337254**	SRR12337253	0.518642	SRR12337254	0.507205

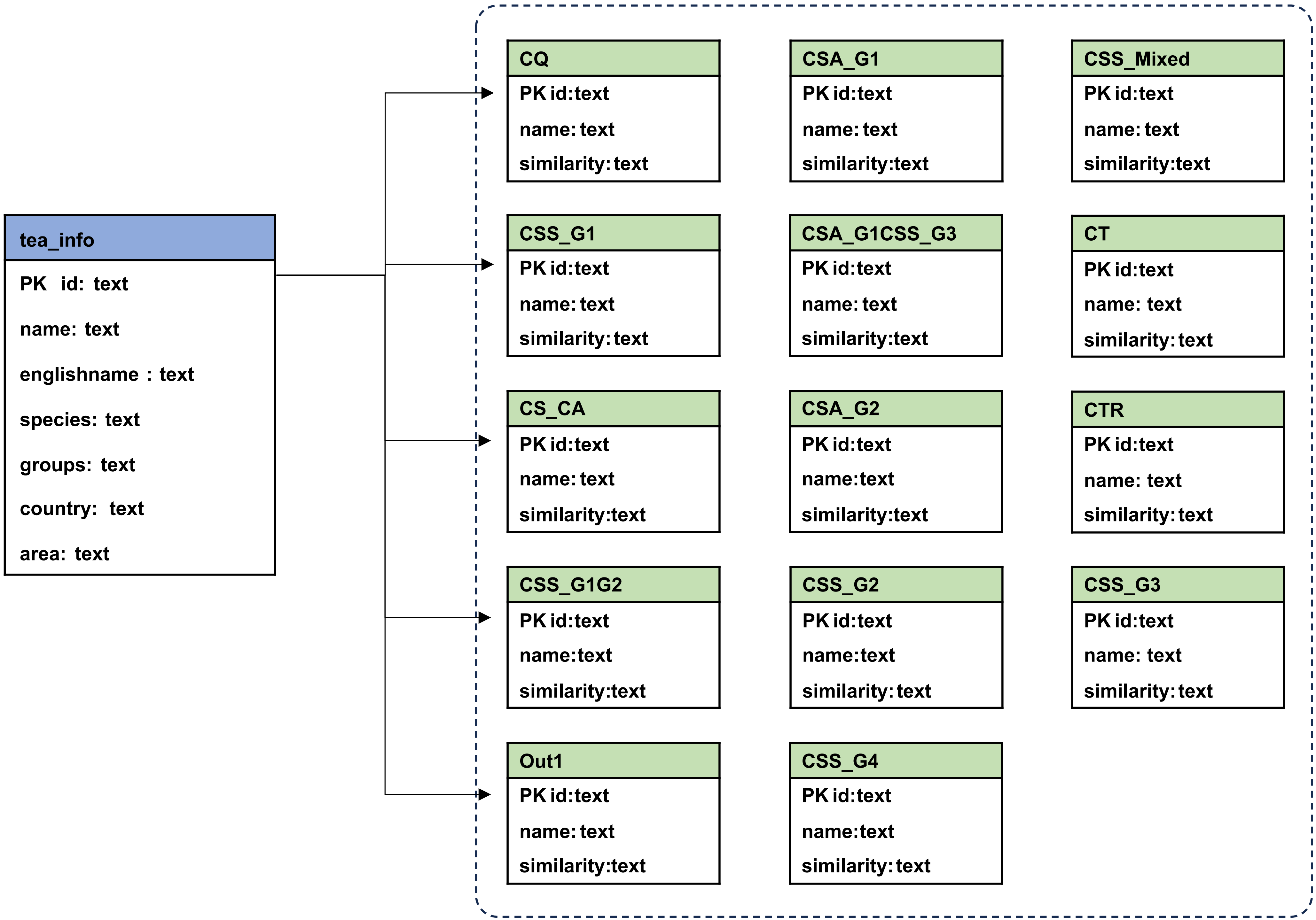
tea_info
PK id: text
name: text
englishname : text
species: text
groups: text
country: text
area: text
CQ
PK id:text
name: text
similarity: text
CSA_G1
PK id:text
name: text
similarity:text
CSS_Mixed
PK id:text
name: text
similarity:text
CSS_G1
PK id:text
name: text
similarity: text
CSA_G1CSS_G3
PK id:text
name: text
similarity:text
CT
PK id:text
name: text
similarity: text
CS_CA
PK id:text
name: text
similarity:text
CSA_G2
PK id:text
name:text
similarity:text
CTR
PK id:text
name: text
similarity: text
CSS_G1G2
PK id:text
name:text
similarity:text
CSS_G2
PK id:text
name:text
similarity: text
CSS_G3
PK id:text
name: text
similarity: text
Out1
PK id:text
name: text
similarity:text
CSS_G4
PK id:text
name:text
similarity: text

Figure

Click here to access/download;Figure;Figure-2.pdf

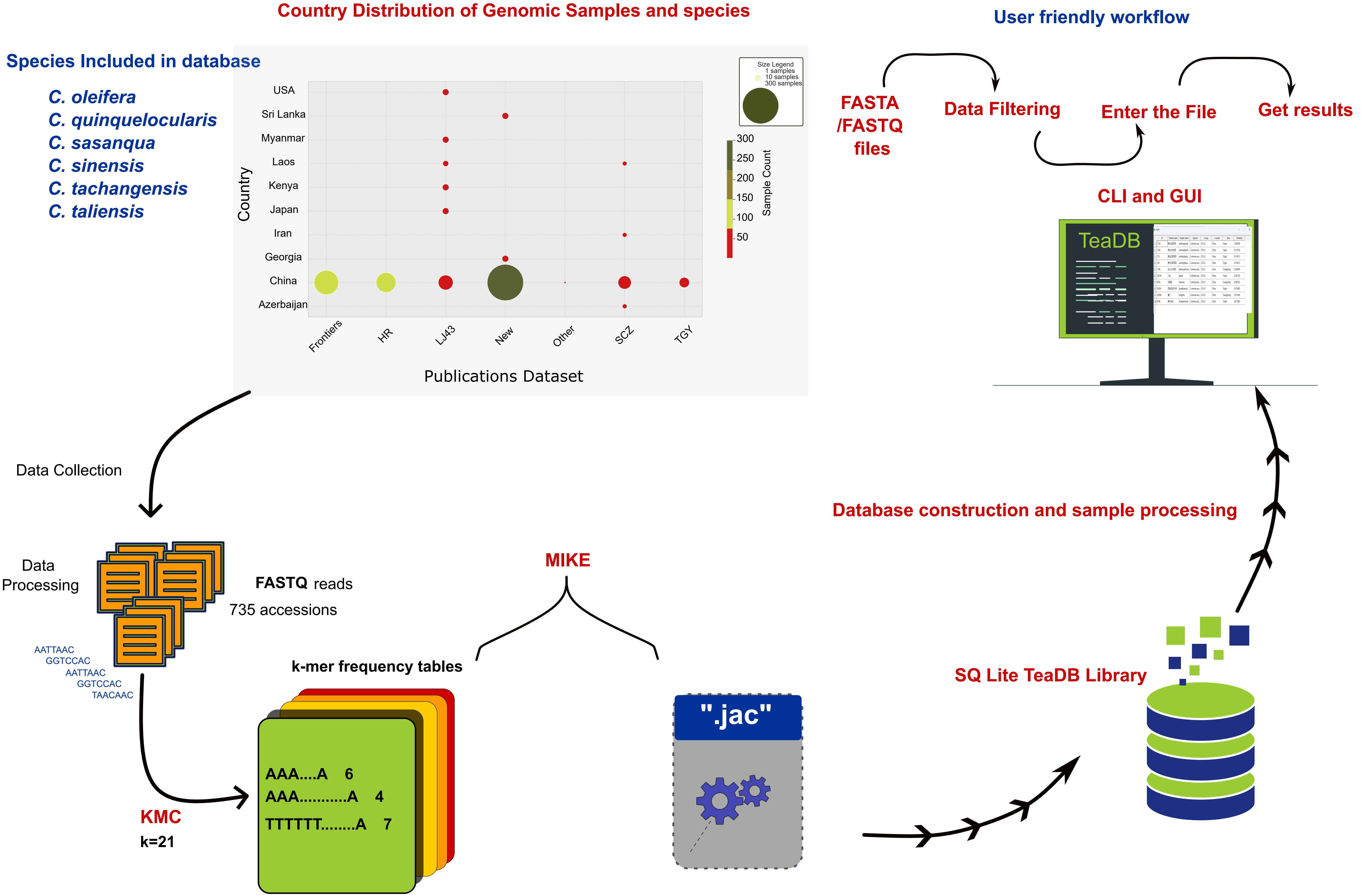

Figure
Click here to access/download;Figure;Figure-3.pdf

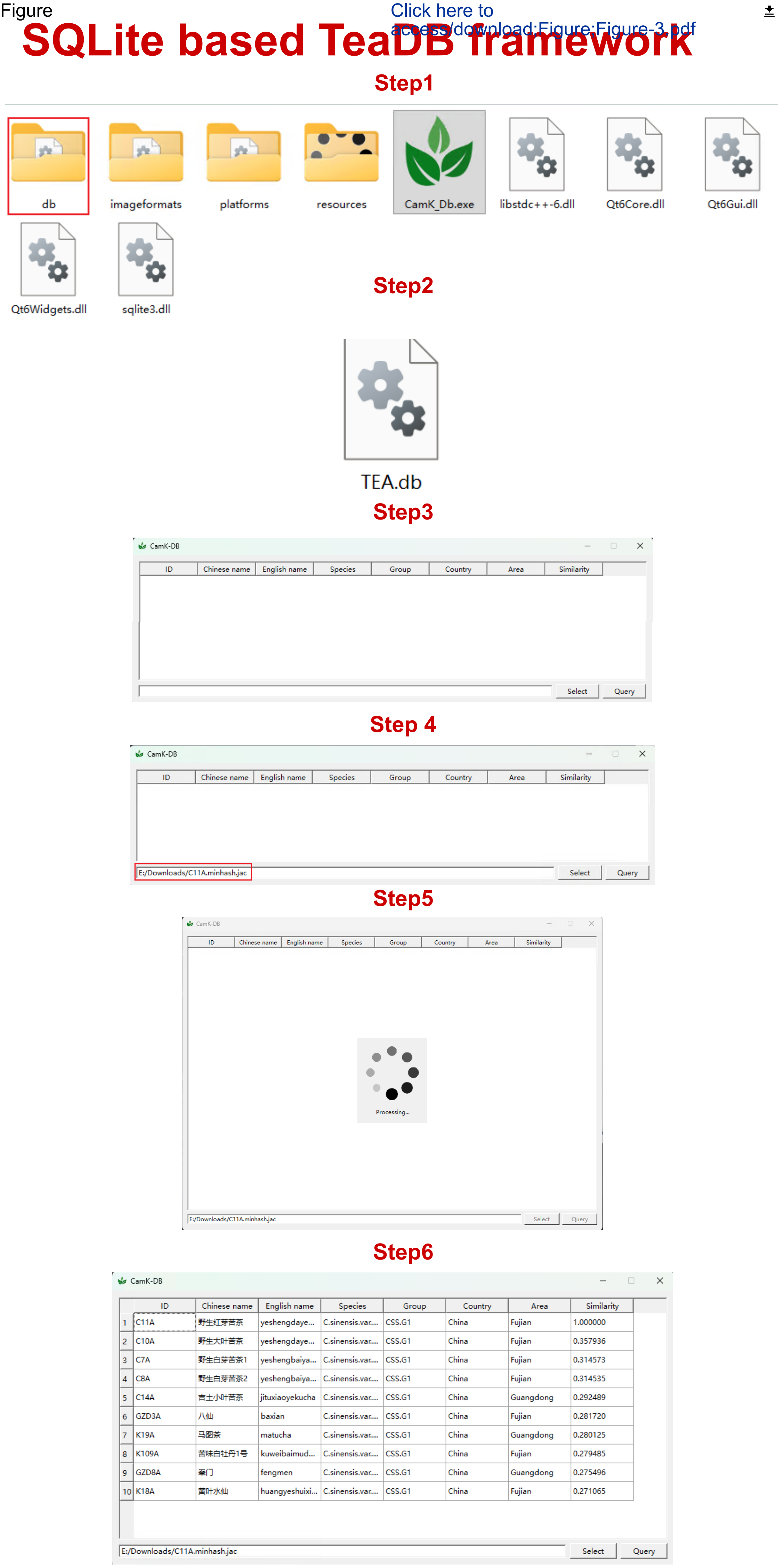

Click here to access/download
Supplementary Material
Supplementry file-1.xlsx

2025-12-30

Editor-in-Chief

GigaScience,

Dear Editors,

I am pleased to submit our manuscript entitled **"Cam*K*-DB: A *k*-mer MinHash fingerprint database for reference-free genotyping of *Camellia* accessions"** for consideration in *GigaScience*. This work describes the development and validation of TeaDB, a comprehensive DNA-fingerprinting resource built from whole-genome resequencing of **735 Camellia accessions** spanning 8 countries, and represented by compact MinHash sketches (k-mer = 21; sketch size = 1,000) stored in an SQLite database linked to rich phenotypic and geographic metadata. The database enables rapid, alignment-free similarity estimation via Jaccard coefficients, overcoming computational barriers of traditional methods.

This manuscript has not been previously published or submitted elsewhere. All authors approve this submission and declare no competing interests. We believe TeaDB's scalable, alignment-free framework will be a valuable community resource for cultivar authentication, phylogenetic analysis, and breeding strategy development.

Thank you for considering our submission. We look forward to your feedback.

Best regards,

Xingtan Zhang
Research Professor
Agricultural Genomics Institute at Shenzhen
Shenzhen, 518120, Guangdong, China
E-mail: zhangxingtan@caas.cn
