# Supplementary material for: CamK-DB: A k-mer MinHash fingerprint database for reference-free genotyping of Camellia accessions
Source: Gigascience. 2026 Aug 21;15:giag088. doi: 10.1093/gigascience/giag088 (PMC13548848; doi:10.1093/gigascience/giag088)
Supplement: Supplementary file 3 [file giag088_giga-d-25-00529_revision_1.pdf]

GigaScience

CamK-DB: A k-mer MinHash fingerprint database for reference-free genotyping of Camellia accessions

--Manuscript Draft--

**Manuscript Number:**	GIGA-D-25-00529R1
**Full Title:**	CamK-DB: A k-mer MinHash fingerprint database for reference-free genotyping of Camellia accessions
**Article Type:**	Technical Note
**Funding Information:**	National Natural Science Foundation of China grant (32222019) — Prof. Xingtan Zhang<br>Shenzhen Science and Technology Program (RCYX20210706092103024) — Prof. Xingtan Zhang<br>Open Fund of Collaborative Innovation Center of Chinese Oolong Tea Industry (2025W 01) — Prof. Xingtan Zhang<br>Project of Science and Technology Plan of Nanping city (N2024Z007) — Prof. Xingtan Zhang<br>Project of Science and Technology Plan of Fujian Province (2023N5013) — Prof. Xingtan Zhang<br>Project of Science and Technology Plan of Fujian Province (2024N3013) — Prof. Xingtan Zhang<br>Scientific Research Fund of Wuyi University (2018N2004) — Prof. Xingtan Zhang<br>Scientific Research Fund of Wuyi University (2021L3058) — Prof. Xingtan Zhang<br>Special Funds for Colleges and Universities in Fujian Province to Improve the Level of Running Schools (No.77 [2022]) — Prof. Xingtan Zhang
**Abstract:**	Tea (Camellia sinensis L.), a major global economic crop in Asia, poses challenges for genetic identification because its highly heterozygous, repetitive genome reduces the efficacy of conventional single-nucleotide polymorphism (SNP) and microsatellite markers, and interspecific hybridization further complicates the situation. To address these issues, CamK-DB was developed as a reference-free Camellia fingerprinting database built on MIKE MinHash sketches. We curated 418 candidate resequencing datasets, and built a database using standardized 5× genome-coverage fingerprints. Each accession is stored as a MIKE .jac fingerprint generated with k=21 and recommended sketch/pre_cnt=2000. CamK-DB provides a command-line interface for data management and a custom C++ query engine that computes top-10 matches using Jaccard similarity, complemented by a QT-based graphical interface for interactive analysis. This resource offers a robust and scalable framework for precise and routine germplasm identification, genomic phylogenetic inference, and strategic breeding program design.
**Corresponding Author:**	Xingtan Zhang<br>Chinese Academy of Agricultural Sciences Agricultural Genomes Institute at Shenzhen<br>Shenzhen, --- Select One --- CHINA
**Corresponding Author Secondary Information:**	
**Corresponding Author's Institution:**	Chinese Academy of Agricultural Sciences Agricultural Genomes Institute at Shenzhen
**Corresponding Author's Secondary Institution:**	

Powered by Editorial Manager® and ProduXion Manager® from Aries Systems Corporation

First Author:	Feiquan Wang
First Author Secondary Information:	
Order of Authors:	Feiquan Wang
	Noor ul Ain, Ph.D.
	Fang Wang, Ph.D.
	Shengcheng Zhang, Ph.D.
	Weilong Kong, Ph.D.
	Yutao Shi, Ph.D.
	Hua Feng, Ph.D.
	Bo Zhang, Ph.D.
	Xingtan Zhang, Ph.D.
Order of Authors Secondary Information:	
Response to Reviewers:	Response to Reviewers<br>Manuscript ID: GIGA-D-25-00529<br>Title: CamK-DB: A k-mer MinHash fingerprint database for reference-free genotyping of Camellia accessions<br>Dear Editor and Reviewers,<br>We sincerely thank the Editor and both reviewers for their constructive comments and helpful suggestions. We have substantially revised the manuscript to improve its methodological rigor, parameter justification, database transparency, reproducibility, and practical user guidance. The major revisions include: (i) We redownloaded ~13TB raw data and reconstructed the final CamK-DB release as a 5× coverage-standardized database; (ii) clarification that 735 candidate resequencing datasets were initially curated, of which 418 accessions passed the quality and coverage requirements and were retained in the final standardized TEA.db database; (iii) expanded explanation of the MIKE/MinHash algorithm, including prefix/suffix grouping and exact matching of fingerprint elements; (iv) addition of coverage-matching and same-coverage replicate stability analyses; (v) addition of sketch-size sensitivity analysis supporting sketch/pre_cnt = 2000; (vi) revision of the mixed-read experiment as mixed-sample or dual-source signal detection rather than formal parentage inference; (vii) improved validation using phylogenetic-distance concordance and out-of-database query interpretation; and (viii) redesigned figures and expanded user-guide instructions.<br>We have also updated the public repository, supplementary tables, and figure files to reflect the revised database construction and validation strategy.<br>Best regards,<br>Xingtan Zhang<br>Research Professor<br>Agricultural Genomics Institute at Shenzhen<br>Shenzhen, 518120, Guangdong, China<br>E-mail: zhangxingtan@caas.cn<br><br>Reviewer #1<br>We sincerely thank Reviewer 1 for the constructive comments and valuable suggestions, which substantially improved the scientific clarity and presentation of the manuscript. We kindly invite the reviewer to examine the revised main and supplementary figures, which have been updated in response to these comments.<br><br>Major Comment 1: “Justification for Algorithm Selection (Lines 66-68): The manuscript mentions several hashing-based techniques (MinHash, Bloom filter, Count-Min Sketch) but provides no rationale for selecting MinHash over others, particularly Bloom filters, which are also known for efficiency in handling large genomic datasets. Please clarify the comparative advantages of MinHash in this specific context (e.g., for similarity estimation via Jaccard index vs. membership queries) and whether a formal comparison was conducted to justify this choice as optimal for the stated objectives of rapid, accurate germplasm identification and comparison.”

Powered by Editorial Manager® and ProduXion Manager® from Aries Systems Corporation

Response: We thank the reviewer for this helpful suggestion. We have revised the Background and Methods sections to clarify why MinHash was selected for CamK-DB. The objective of CamK-DB is rapid similarity estimation and accession ranking, rather than membership querying or abundance estimation. In the MIKE framework, reads are decomposed into k-mers, deduplicated k-mer information is grouped by prefix, and each fingerprint element is represented as a `(prefix, minhash(suffix))` pair. Jaccard similarity is then calculated by comparing these MIKE-derived fingerprint-element sets between query and database samples. MinHash is well suited behind the rationale of our database because it provides a compact approximation of Jaccard set resemblance. In contrast, Bloom filters are mainly designed for approximate membership queries and may introduce false-positive overlap when adapted for similarity estimation, whereas Count-Min Sketch is mainly used for approximate frequency estimation rather than direct set-similarity comparison. Therefore, although these methods are useful for other k-mer applications, MinHash is more directly aligned with CamK-DB's goal of reference-free similarity ranking. We did not perform a formal head-to-head benchmark against Bloom filters or Count-Min Sketch in this study. The choice was task-driven and based on MIKE, which was specifically developed for fast MinHash-based similarity estimation from sequencing reads Lines 63-77 and121-152.

Major Comment 2: "Choice of k-mer Size (k=21) (Lines 98-100): The selection of k=21 is stated as the default/recommended setting in MIKE but is not biologically or computationally justified within this study. Please provide the reasoning behind this specific value. Were other k-mer lengths evaluated (e.g., 15, 31, 51)? If so, please present a brief analysis (e.g., in supplementary material) showing how different k values affect discrimination power, runtime, or database size. If not, please acknowledge this as a limitation or standard parameter from prior work and discuss its suitability for tea genomes."

Response: We thank the reviewer for this helpful comment. We have revised the Methods section to better justify the use of k=21. This parameter was selected based on the original MIKE study, where simulated datasets with 40× coverage were tested using various k=17, 19, 21, 25, and 27. That analysis showed that k=21 provided a stable balance between similarity-estimation accuracy and robustness, while larger k values showed increasing deviation, likely because longer k-mers are more prone to sequencing errors and per nucleotide variation. We also added a Camellia-specific explanation. Tea genomes are large, highly heterozygous, and repeat-rich; therefore, shorter k-mers may produce non-specific matches in repetitive regions, whereas longer k-mers may reduce sensitivity because single-base differences disrupt the entire k-mer. Thus, k=21 was retained as the MIKE-supported criterion for CamK-DB fingerprint generation. We did not perform a new CamK-DB-specific k-mer sweep in this revision as it was pre-validated in our previous work Lines 106-119 and 381-388..

Major Comment 3: "Use of Unique k-mers and Data Heterogeneity (Lines 100-103; 225-234): The manuscript states that unique k-mers are used to build MinHash fingerprints. Given the noted variability in sequencing depth, data volume, mapping rate, and genome coverage among the 735 accessions (Table S1), it is crucial to address: What is the approximate count of unique k-mers per sample, and how does it vary? How does variability in sequencing depth and data quality impact the number and stability of unique k-mers extracted? What normalization or standardization steps (if any) were applied to mitigate biases introduced by such technical heterogeneity before sketch generation and similarity calculation?"

Response: We thank the reviewer for raising this important point. We have revised the manuscript to clarify the terminology. Originally the use of "unique k-mers" was potentially misleading. CamK-DB does not use unique k-mers in the sense of biological single-copy, accession-specific, or sample-private k-mers. Instead, MIKE first deduplicates k-mer sequence identities and then converts them into compact prefix-minhash fingerprint elements. Specifically, reads are decomposed into 21-mers. Each 21-mer is divided into a 10-base prefix and an 11-base suffix. K-mers with the same prefix are grouped, and MIKE records a minimized hash-derived value from the suffix group. Because the prefix length is 10 bases, the theoretical feature space is fixed at 4^{10} = 1,048,576 possible prefix groups. Thus, the `.jac` fingerprint is a compact list of prefix-minhash feature values rather than a direct list of raw unique k-mers. We have revised the Methods section to explain this clearly. We also addressed the reviewer's concern about sequencing-depth heterogeneity. Because different sequencing depths can lead to estimation of different subsets of k-mers before minimization, fingerprints generated from unequal coverage may show reduced similarity even for the same

accession. We therefore tested fingerprints generated at different coverage levels and found that similarity was highest when query and database fingerprints were generated at matched coverage. To mitigate this bias, we rebuilt the final CamK-DB release using standardized 5× genome-coverage sampling. Of the 735 candidate datasets initially curated, 418 accessions met the quality and coverage requirements for standardized 5× fingerprint generation and were retained in the final TEA.db database. Query samples are now recommended to be downsampled to 5× before MIKE fingerprint generation. Lines 119-137, Table S1,S5, and S3.

Major Comment 4 : Jaccard Similarity Calculation and Its Robustness (Lines 107-115):

The method for calculating Jaccard similarity based on shared unique k-mers requires further elaboration:

How is a "shared unique k-mer" defined? Does it require an exact sequence match (presumably yes for k-mers), or are mismatches allowed?

Are the sets S1 and S2 (the unique k-mer sets from two samples) required to be of comparable size? If two samples have vastly different numbers of unique k-mers (e.g., due to different coverage), how does this affect the Jaccard index? Have the authors evaluated the sensitivity of the similarity score to such disparities?

Please describe any preprocessing, filtering, or scaling procedures implemented to ensure fair comparisons between samples with differing k-mer set sizes.

Response: We thank the reviewer for this important comment. We have revised the manuscript to clarify that CamK-DB does not calculate Jaccard similarity from raw “unique k-mer” sets in the biological sense of single-copy or accession-specific k-mers. Instead, CamK-DB uses MIKE-derived fingerprint-element sets. Briefly, reads are decomposed into 21-mers, identical 21-mers are deduplicated, and each 21-mer is divided into a 10-base prefix and an 11-base suffix. K-mers with the same prefix are grouped, and MIKE records a minimized hash-derived value from the suffix group. Each fingerprint element is therefore represented as a (prefix, minhash(suffix)) pair. A shared element requires an exact match of both the prefix and the minhash(suffix) value between the query and database fingerprint. However, mismatches are not allowed. In the revised manuscript, S_1 and S_2 are now defined as MIKE-derived fingerprint-element sets rather than raw unique k-mer sets, and Jaccard similarity is calculated as $|S_1 \cap S_2| / |S_1 \cup S_2|$.

To address the k-mer sizes, coverage related nuisance on Jaccard’s index, we also applied a test to validate that if the coverage of reads should be comparable, We tested this by generating fingerprints from the same accession at different genome coverage levels [1,2,3,4,5,10,15,20] fold of genome size, then used these dataset to extract minhash fingerprint, which were used to construct database. The results showed that similarity was highest when query and database fingerprints were generated at the same coverage and decreased when coverage differed.

To reduce this technical bias and ensure fair comparison, we reconstructed the final CamK-DB release using standardized 5× genome-coverage sampling. Of the 735 candidate Camellia resequencing datasets initially curated, 418 accessions met the quality and coverage requirements for standardized 5× fingerprint generation and were retained in the final TEA.db database. Query samples are now recommended to be downsampled to 5× before MIKE fingerprint generation to ensure comparability with the standardized database. Lines 121-152, 187-192, 270-279, and 372-380.

Major Comment 5: “Rationale for Sketch Size (1000) (Lines 118-120): The use of a”1000-sketch size” is mentioned without justification. This parameter controls the resolution and accuracy of the MinHash approximation. What was the basis for choosing a sketch size of 1000? Was a sensitivity analysis performed with different sketch sizes (e.g., 500, 2000, 5000)? Please provide evidence or references showing that a size of 1000 provides an optimal balance between accuracy, storage, and computational speed for this specific dataset and application.”

Response: We agree that this parameter required empirical justification. Therefore, we performed a sketch-size sensitivity analysis using values of 100, 500, 1000, 2000, 5000, 8000, and 10000. For each setting, we compared the sketched similarity estimates with the full fingerprint comparison and calculated RMSE, while also recording runtime. The results showed that RMSE decreased substantially as sketch size increased from very small values and became stable around 2000. Larger sketch sizes increased runtime without proportional improvement in accuracy. Based on this analysis, we revised the manuscript to recommend sketch/pre_cnt=2000 for high-confidence CamK-DB analyses. The abstract, Methods, User Guide, figure labels, and supplementary results have been updated accordingly Lines 162-172, 280-290, and

	433-441.<br>Major Comment 6: “Clarity on Input Requirements and Tool Robustness (User Guide Section): The user guide effectively describes how to use CamK-DB but lacks critical specifications on what data is suitable as input. Minimum Requirements: Please explicitly state the required sequencing platform (or compatibility), recommended minimum sequencing depth, and necessary read length for reliable analysis. Data Quality Thresholds: Specify the tool’s tolerances or requirements for input data quality (e.g., Q-score thresholds, adapter contamination levels). What QC steps are mandatory before running MIKE to generate the”.jac” file? Performance under Variable Depth: How do sequencing depth and coverage influence runtime and, more importantly, the accuracy of identification (e.g., top-hit confidence, similarity score stability)? A brief analysis or statement on the tool’s robustness to suboptimal data would greatly enhance its practical utility guide.”<br>Response: We have expanded the User Guide by adding a dedicated “Input Data Specifications” subsection. The revised manuscript now states that CamK-DB supports FASTQ/FASTA files and precomputed MIKE.jac fingerprints, and that the standardized release was validated primarily using short-read whole-genome resequencing data. We specify the required preprocessing steps, including adapter removal, low-quality read trimming, and removal of reads shorter than 50 bp. We further state that query data should be downsampled to 5× genome coverage before MIKE fingerprint generation to ensure comparability with the standardized TEA.db database. The revised User Guide also explains that fingerprints generated from substantially different coverage levels may show reduced similarity, even when they originate from the same accession. These additions provide practical guidance on suitable input data, quality control, coverage normalization, and interpretation of results Lines 354-388 and 405-426.<br>Minor Comment 1: “Dataset Representativeness and Selection Criteria (Line 188): Please state the average sequencing depth for the entire set of 735 accessions. More importantly, justify the selection of these specific 735 accessions. Do they comprehensively represent the genetic, geographical, and phenotypic diversity of cultivated and wild Camellia? A brief discussion on the representativeness and potential biases of the dataset would be valuable.”<br>Response: We have revised the dataset description for 418 accessions retained in the final 5× standardized CamK-DB release. We added sequencing-quality attributes and other related information to Supplementary Table S1. We also added group-level representation information for the final 418 retained accessions. The revised Discussion acknowledges that although the retained accessions span multiple Camellia species, genetic groups, and geographic origins, the current standardized release is not exhaustive and may underrepresent some geographic regions or rare germplasm groups. Future releases will incorporate additional accessions that meet the 5× standardized fingerprinting requirements Lines 291-299, 315-329, 418-426, and 439-451.<br>Minor Comment 2: “Terminology Accuracy (Lines 217-220): The phrase”deep resequencing data with more than 8× sequencing depth” is misleading. In current genomics standards, “deep” resequencing typically implies coverage ≥30x. Please rephrase to “resequencing data with a minimum depth of 8×” or similar to avoid overstatement.”<br>Response: We agree and have replaced “deep resequencing data with more than 5× sequencing depth” with “resequencing data with a minimum depth of 5×” or equivalent wording throughout the manuscript Lines 327-340.<br>Minor Comment 3: “Completeness of Table S1: Table S1 appears to contain detailed metrics only for newly sequenced data, leaving fields blank for publicly sourced accessions. To ensure transparency and reproducibility, please supplement the table with available metrics (e.g., SRA accession-linked depth, read counts if obtainable) for all 735 accessions or clearly note the source of each datum.”<br>Response: We have expanded Supplementary Table S1, S3 and S5 to include available metadata and quality information for all 418 candidate datasets, including public accession identifiers, source references, estimated sequencing depth or read count where available, and retained/excluded status in the final standardized database.<br>Minor Comment 4: “Expanded User Guide: As noted in Major Comment 6, the User Guide section should be expanded to include a dedicated subsection on”Input Data Specifications” detailing platform, depth, quality control prerequisites, and file format requirements.”<br>Response: We have added the requested “Input Data Specifications” subsection to the

User Guide. It describes accepted input formats, validated sequencing data type, quality-control requirements, 5× downsampling requirement, MIKE parameter settings, and interpretation of ranked results Lines 354-388 and 405-417.

Minor Comment 5: "Validity for Samples Outside the Database (Technical Validation): The validation convincingly shows performance for samples and mixtures within the database. Please comment briefly on the expected behavior or interpretive value when querying a sample whose true parents or close relatives are not in CamK-DB. How should users interpret the top hits in such scenarios?"

Response: We have added guidance for out-of-database queries. CamK-DB should be interpreted as a nearest-neighbor fingerprinting system. If the exact accession or a very close relative is absent from the database, the top hit should not be treated as a definitive identity assignment. Instead, the top-ranked accessions represent the closest available genomic neighbors under the current standardized database. We added an out-of-database query example showing that the highest-similarity database hits corresponded to accessions with relatively low genetic distance, supporting the use of CamK-DB for nearest-neighbor interpretation when exact matches are absent. Lines 255-269 and 405-417

Minor Comment 6: "Abbreviation Formatting (Lines 58-59): The first instance of an abbreviation (e.g., likely"" or similar from context) should be spelled out in full."

Response: We have revised the manuscript to spell out abbreviations at first use and added an alphabetical abbreviation list Lines 54-61

Minor Comment 7: "Formatting Consistency: Line 103:"k-mers" - the 'k' should be italicized (k-mers). Line 107: "MinHash" - ensure consistent formatting; it should not be italicized if it is not standard elsewhere. Line 115: "S_2" should use subscript for the numeral. Add a space: "sample 2"."

Response: We have corrected formatting throughout the manuscript. The term k-mer is now formatted consistently, MinHash is presented consistently, S1 and S2 are formatted with subscripts where appropriate, and "sample2" has been corrected to "sample 2". Lines 63-80 and 106-152

Minor Comment 8: "Figure Reference (Lines 110-112): The text referencing"Figure 1" describes a mathematical formula, but the current Figure 1 appears to depict database schema. Please verify all figure citations and ensure they accurately correspond to the described content. The formula for Jaccard similarity might be better placed in the main text or a dedicated methodology figure."

Response: We thank the reviewer for identifying this mismatch. We have handles in text figure citations and re-organized the figures. Lines 147-152 and 557-582.

Reviewer #2

We sincerely thank Reviewer 2 for the careful evaluation of our manuscript and the insightful recommendations that helped strengthen the methodology and technical validation. We kindly ask the reviewer to review the updated figures and supplementary materials, in which the requested analyses and clarifications have been incorporated.

Major Comment 1: "Justification of Algorithmic Parameters. The manuscript utilizes the MIKE algorithm with default parameters (k=21 and sketch size=1000) but falls short of providing a sufficient theoretical or empirical basis for these choices. Given that k-mer-based analyses are highly sensitive to parameter selection—particularly in the context of the tea genome, which is characterized by high heterozygosity and a high proportion of repetitive elements—the mere adoption of default settings is inadequate. The authors should elaborate on the underlying algorithmic mechanics, such as the specific hash functions employed, and discuss how the selected k-mer size balances the trade-off between specificity and the noise introduced by sequencing errors or genomic complexity."

Response: We agree and have substantially expanded the algorithmic description. The revised Methods now explain that MIKE decomposes reads into k-mers, encodes k-mers, groups them by prefix, and represents each group using the minimum hash value of the suffix. These encoded (prefix, minhash(suffix)) fingerprint elements are stored as .jac files and compared using Jaccard similarity. We also expanded the rationale for k=21, explaining the trade-off between short k-mers, which may be non-specific in repetitive genomes, and long k-mers, which are more sensitive to sequencing errors and heterozygous variation. In addition, we performed sketch-size sensitivity analysis and revised the recommended sketch/pre_cnt value from 1000 to 2000 based on RMSE and runtime results. The revised Figure 2 now illustrates the MIKE fingerprint-generation process in greater technical detail. Lines 106-172 and 557-565

Powered by Editorial Manager® and ProduXion Manager® from Aries Systems Corporation

Major Comment 2: “Rigor of Validation Strategy. The technical validation presented in the study is currently insufficient as it relies heavily on”self-identification,” where accessions used to construct the database are simply re-queried against it. This circular approach essentially confirms the database’s internal consistency but fails to demonstrate the tool’s robustness and generalizability when faced with independent, external datasets. To truly validate the utility of CamK-DB in a real-world setting, the authors should perform cross-validation or, ideally, test the system with newly sequenced samples that were strictly excluded from the database construction process to prove it can accurately identify samples it has not “seen” before.”
Response: We thank the reviewer for this important comment. We agree that self-identification alone is insufficient to demonstrate the broader utility of CamK-DB. In the revised manuscript, we now present self-identification only as an internal consistency control and added additional validation analyses to evaluate whether CamK-DB similarity scores reflect biological relatedness and whether the database can provide meaningful nearest-neighbor information for samples not included in TEA.db.
First, using the standardized 5× database, we queried ten accessions represented in TEA.db and found that the same accession was recovered as the top-ranked hit in all cases. The primary similarities ranged from 0.544 to 0.607, and the mean top1–top2 similarity margin was 0.272. This confirms internal recovery of represented accessions under the standardized workflow.
Second, we compared CamK-DB similarity profiles with genetic distances derived from the published Camellia phylogenetic tree. Because genetically close samples are expected to have higher CamK-DB similarity and lower phylogenetic distance, a negative correlation between similarity and distance supports the biological relevance of the database-derived similarity scores. The revised analysis showed that most samples displayed negative correlations between CamK-DB similarity and phylogenetic distance, indicating that the similarity scores are broadly consistent with phylogenetic relationships.
Third, we evaluated an out-of-database query sample, SRR11672521, which was not included in the standardized CamK-DB release. Although the exact accession was absent from the database, higher CamK-DB similarity was associated with lower phylogenetic distance to database accessions ($r = -0.37$, $P = 4.79 \times 10^{-15}$). This supports the use of CamK-DB as a nearest-neighbor fingerprinting system when exact matches are absent, while also clarifying that top hits should not be interpreted as definitive identities if the query accession or a close relative is not represented in the database.
Finally, we assessed group-level representation in the standardized 418-accession database. The retained accessions remained distributed across the major Camellia groups included in CamK-DB, with a median group size of 16, supporting the utility of the standardized database for broad group-level comparison. These additions have been incorporated into the revised Technical Validation, User Guide, and Discussion sections. Figure 2C, D, Lines 212-299, 405-417, and 602-615
Major Comment 3: “Limitations in Parentage Inference Methodology. The assessment of parentage inference is methodologically limited by the use of in silico mixed reads to simulate hybridization. While this approach effectively demonstrates the tool’s ability to deconvolve mixed genomic signals from two different sources, it does not accurately reflect true biological hybridization. Biological hybrids undergo meiotic recombination and segregation, resulting in a unique k-mer composition that differs significantly from a simple linear physical mixture of parental reads. The authors must clarify this distinction in the text and explicitly acknowledge that their current simulation primarily tests the detection of mixed samples rather than the inference of complex genealogical ancestry or hybrid origins.”
Response: We completely agree with the reviewer. We have revised this section throughout the manuscript. The experiment is no longer described as formal parentage inference or biological hybrid identification. Instead, it is now presented as a mixed-sample or dual-source signal detection test.
In the revised analysis, artificial mixed-read datasets were generated by combining reads from pairs of accessions represented in the standardized database. In all ten mixtures, the two highest-ranked database records corresponded to the two source accessions used to generate the query dataset. The primary similarity values ranged from 0.386 to 0.504, and the secondary similarity values ranged from 0.372 to 0.489. This demonstrates that CamK-DB can recover dual-source signals from artificial mixed-read data when both source accessions are present in the database.
We now explicitly state that physical mixing of reads from two accessions does not

Powered by Editorial Manager® and ProduXion Manager® from Aries Systems Corporation

	model true biological hybridization, because biological hybrids inherit recombined haplotypes following meiosis and segregation. Therefore, this analysis should be interpreted as mixed-sample signal detection, not as formal biological parentage or hybrid-origin inference. Formal hybrid-origin or parentage inference would require validated F1 progeny or inheritance-aware analyses. Lines 233-254, 418-424, and 608-615<br>Minor Comment 4: "Database Architecture Clarity (Figure 1). The description of the database architecture in Figure 1 requires significantly more detail to be transparent and reproducible. Specifically, the manuscript does not adequately explain the rationale behind partitioning the database into 13 distinct grouping tables, nor does it define the cryptic abbreviations used for these sub-tables. A clear mapping key or a more descriptive legend is necessary to help readers understand the logical organization and taxonomy of the stored data."<br>Response: We have improved the explanation about this figure which is now Figure 3, and explained the information of retained 418 standardized accessions, the central tea_info metadata table, the group-specific fingerprint tables, and the relationship between metadata and MIKE-derived fingerprint records. We also added abbreviation key for all group-table names and explained the rationale for group-level partitioning. Lines 193-211, 447-451, and 577-582. Figure 1.<br>Minor Comment 5: "Quality of Visualizations (Figure 2). Figure 2 is currently too schematic and lacks the necessary technical depth to fully illustrate the study's workflow. It fails to capture critical details of the MIKE algorithm, such as the hashing process or sketch generation, and the visual presentation is somewhat rudimentary for a high-impact publication. The authors should refine this figure to provide a more comprehensive and professionally polished representation of the computational pipeline that visually communicates the complexity of the methods used."<br>Response: We agree and have completely redesigned Figure 2 which is Figure 1 now. The revised figure now presents the full MIKE-based fingerprint-generation workflow, including read preprocessing, 5× coverage standardization, KMC k-mer counting, k=21 setting, prefix/suffix grouping, MinHash-based sketching, .jac fingerprint generation, SQLite database construction, and Jaccard-based top-hit ranking. The figure has been redesigned with clearer panel structure, consistent terminology, and publication-quality formatting. Lines 106-172 and 557-565 , Figure 1
**Additional Information:**	
**Question**	**Response**
Are you submitting this manuscript to a special series or article collection?	No
**Experimental design and statistics**<br><br>Full details of the experimental design and statistical methods used should be given in the Methods section, as detailed in our Minimum Standards Reporting Checklist. Information essential to interpreting the data presented should be made available in the figure legends.<br><br>Have you included all the information requested in your manuscript?	No
If not, please give reasons for any omissions below.	This submission is a Technical Note describing a computational/database resource and its validation. There were no new wet-lab experiments, interventions, randomization, blinding, or health-related outcomes, and no hypothesis-testing statistics (e.g., p-values/power calculations) were required for the aims of this work. All

as follow-up to "**Experimental design and statistics**<br><br>Full details of the experimental design and statistical methods used should be given in the Methods section, as detailed in our Minimum Standards Reporting Checklist. Information essential to interpreting the data presented should be made available in the figure legends.<br><br>Have you included all the information requested in your manuscript?<br><br>"	applicable methodological details are provided in the Methods and figure legends, including data sources, preprocessing steps, algorithm parameters (e.g., k-mer size/sketch size), similarity metric, and the validation/benchmarking procedure. Items from the Minimum Standards Reporting Checklist that relate to experimental interventions or inferential statistical testing are not applicable to this study.
**Resources**<br><br>A description of all resources used, including antibodies, cell lines, animals and software tools, with enough information to allow them to be uniquely identified, should be included in the Methods section. Authors are strongly encouraged to cite Research Resource Identifiers (RRIDs) for antibodies, model organisms and tools, where possible.<br><br>Have you included the information requested as detailed in our Minimum Standards Reporting Checklist?	Yes
**Availability of data and materials**<br><br>All datasets and code on which the conclusions of the paper rely must be either included in your submission or deposited in publicly available repositories (where available and ethically appropriate), referencing such data using a unique identifier in the references and in the "Availability of Data and Materials" section of your manuscript.<br><br>Have you have met the above requirement as detailed in our Minimum	Yes

Standards Reporting Checklist?	
GigaScience has policies and guidelines in place for the use of generative AI-writing tools such as ChatGPT. If you have used such writing tools to assist with writing the manuscript this must be declared and cited in the text. Authors should not list AI-writing tools and other AI-assisted technologies as an author or co-author and should acknowledge that they are fully responsible for text generated or refined by AI-writing tools.<p><br><br>A summary of use (particularly in the introduction or among methods) needs to be included at the end of the paper, and the outputs should also be included as a supplementary file hosted in GigaDB or other open repositories. Please read our guidelines for more information. <p><br><br>By submitting to GigaScience, you are aware of the journal's AI-writing tools policy, and if you have declared use of such tools below, you have acknowledged this where appropriate in your manuscript and have made a summary of use and outputs available. <p><br><b>AI-assisted writing tools have been used in the preparation of this manuscript?	Yes

Manuscript

Click here to access/download;Manuscript;Revised_Article-81726.docx

Cam*K*-DB: A *k*-mer MinHash fingerprint database for reference-free genotyping of *Camellia* accessions

Feiquan Wang[1,#], Noor-ul-Áin[2,#], Fang Wang[2,#], Shengcheng Zhang[2,#], Weilong Kong[2], Yutao Shi[1], Hua Feng[1], Bo Zhang[1,*], Xingtan Zhang[2,*]

[1] College of Tea and Food Sciences, Collaborative Innovation Center of Chinese Oolong Tea Industry, Wuyi University, Tea Engineering Research Center of Fujian Higher Education, Wuyishan Fujian, 354300, China

[2] State Key Laboratory of Tropical Crop Breeding, Shenzhen Branch, Guangdong Laboratory for Lingnan Modern Agriculture, Genome Analysis Laboratory of the Ministry of Agriculture and Rural Affairs, Agricultural Genomics Institute at Shenzhen, Chinese Academy of Agricultural Sciences, Shenzhen, 518120, China

# These authors contributed equally

* Correspondence to Xingtan Zhang (zhangxingtan@caas.cn) and Bo Zhang (Zhangbo@wuyiu.edu.cn)

Fei quan WANG [0009-0003-1337-4052]; Noor-ul-Áin [0009-0008-6240-3274]; Fang Wang [0009-0007-9005-2072]; Shengcheng Zhang [0000-0002-3949-4269]; Weilong Kong [0000-0002-6066-0508]; Yutao Shi [0000-0003-2560-7143]; Hua Feng [0009-0000-4854-3512]; Bo Zhang [0009-0003-7777-5373]; Xingtan Zhang [0000-0002-5207-0882].

Abstract

Tea (*Camellia sinensis* L.), a major global economic crop in Asia, poses challenges for genetic identification because its highly heterozygous, repetitive genome reduces the efficacy of conventional single-nucleotide polymorphism (SNP) and microsatellite markers, and interspecific hybridization further complicates the situation. To address these issues, CamK-DB was developed as a reference-free *Camellia* fingerprinting database built on MIKE MinHash sketches. We curated 418 candidate resequencing datasets, and built a database using standardized 5× genome-coverage fingerprints. Each accession is stored as a MIKE .jac fingerprint generated with k=21 and recommended sketch/pre_cnt=2000. Cam*K*-DB provides a command-line interface for data management and a custom C++ query engine that computes top-10 matches using Jaccard similarity, complemented by a QT-based graphical interface for

interactive analysis. This resource offers a robust and scalable framework for precise and routine germplasm identification, genomic phylogenetic inference, and strategic breeding program design. Cam*K*-DB (database and code) is publicly available at (https://github.com/sc-zhang/CamK-DB). Cam*K*-DB binaries are provided for Windows 10/11 and Linux (x86_64, glibc $\geq$ 2.27).

Keywords: MinHash; *k*-mer, *Camellia sinensis*, alignment-free genomics, Jaccard similarity, germplasm identification, SQLite database

Background

Tea plants (*Camellia sinensis*), famous for their outstanding foliage, are among the world's most cherished non-alcoholic beverages. Beyond its scope as a beverage crop, the tea industry extends to different sectors, such as finished products, tea ware, and global expansions. Technically, *C. sinensis* (L.) is the principal cultivated species for tea production, sectioned as *C. sinensis* var. *sinensis* (small-leaf tea) and *C. sinensis* var. *assamica* (large-leaf tea). Additionally, several wild relatives, including *C. taliensis*, *C. irrawadiensis*, *C. tachangensis*, and *C. grandibracteata*, account for the genetic diversity in China. Tea leaves face many challenges in phenotypic characterization due to their dense foliage, architecture, and phenotypic similarity. Additionally, tea plants exhibit complex genomic features, including high heterozygosity (>2%), frequent interspecific hybridization, extensive vegetative propagation, and a genome dominated by repetitive sequences such as long terminal repeat (LTR) retrotransposons (approximately 70% of the genome) [1]. These characteristics complicate traditional genetic analysis and cultivar identification. Thus, mislabeling and fraudulent substitution of tea varieties undermine consumer trust and economic stability, necessitating a robust anti-counterfeiting measure for the rapid and accurate identification of tea varieties.

Current methods for tea variety identification, such as morphological analysis and chemical profiling, are often time-consuming and lack the precision to distinguish closely related species and varieties [2,3]. Conventional methods of DNA fingerprinting relied on molecular markers (e.g., SSRs/microsatellites, Amplified fragment length polymorphism), allowing cultivar identification and population genetics, but showed limited genome-wide resolution. Reduced representation approaches such as RAD-seq and genotyping-by-sequencing (GBS) based on short reads matured into alignment-based pipelines that infer SNPs against a reference genome [4,5]. Molecular markers,

such as Simple Sequence Repeats (SSRs) and Single Nucleotide Polymorphisms (SNPs), are accurate and efficient but entail high genotyping costs and complexity [2,3]. These methods benefited from multiple-sequence-alignment tools (e.g., BLAST, ClustalW) that remain foundational and widely used [6]. Recently, *k*-mer-based analytical approaches have been extensively utilized in computational biology; however, the determination of an optimal *k*-mer length remains contingent upon heuristic optimization methodologies. Several hashing-based techniques, such as MinHash, Bloom filter, and Count-Min Sketch [7], have been developed or adopted to compute k-mer-based similarity methods efficiently. MinHash efficiently estimates genetic similarity by representing genomes as compact sketches, thereby significantly reducing the computational demands, rather than to perform membership testing or frequency estimation. Although Bloom filters are highly memory-efficient and useful for filtering or indexing large *k*-mer collections, they do not directly provide an unbiased estimate of set similarity; false positives may inflate the apparent overlap between two samples unless additional correction or processing is applied. Similarly, Count-Min Sketch is primarily designed for approximate frequency estimation in streaming data and is therefore more appropriate for abundance-related queries than for direct set similarity comparisons. Inspired by these advancements, we previously developed the MinHash-based *k*-mer algorithm MIKE, tailored to process extensive genomic data, generate compact genetic signatures, and enable rapid similarity calculations [8]. Accordingly, CamK-DB adopts the MIKE MinHash framework for reference-free generation and comparison of compact genomic fingerprints. Tea genomics has recently been endowed with two chromosome-scale assemblies of *C. lanceoleosa* and wild oil-*Camellia* and a haplotype-resolved tetraploid *C. oleifera* genome, providing valuable insights into genome evolution, population structure, self-incompatibility, and agronomic traits [9–12]. Owing to the availability of large-scale genomic datasets, there is a dire need for a centralized and standardized repository that can facilitate the identification of different accessions and varieties irrespective of phenotypic and chemical methods. Many recent studies have contributed to the compilation of valuable datasets in various aspects of tea, such as an atlas of genomic variation, metabolites of tea cultivars, or aspects of tea plant biology [13,14]. Despite these recent advances, a publicly available *k*-mer fingerprint database for identifying tea accessions is still lacking, and Cam*K*-DB has been developed to address this gap. This alignment-free approach compares samples directly via their *k*-mer content,

avoiding read mapping and multiple alignment. MinHash sketches transform large datasets into compact "signatures," enabling rapid, accurate similarity queries with small storage footprints. As it helps in (i) reference-free operation, (ii) good performance at low-to-moderate coverage, (iii) fast search across many accessions, and (iv) easy sharing/indexing of compact sketches. Benefitting from this, we created a *k*-mer based, low-cost, user-friendly database for germplasm identification. The base dataset is a spatially diverse resource of 418 accessions of different species of *Camellia*. Built upon QT architecture and SQLite, the database enables rapid identification of tea tree varieties, offering unprecedented accuracy and efficiency in cultivar authentication, hybrid detection, and evolutionary research.

Theory behind Database Construction

Database construction was based on two key elements.

1. DNA Markers (*k*-mers): The foundation of the database lies in the genetic information obtained from 418 accessions of tea resequencing data followed by using an alignment-free and more accurate approach, i.e., *k*-mer similarity for building a database. These fingerprints were generated using *k*=21 based on our previously developed method MIKE, which employed rigorous evaluation with 40× sequence coverage using various *k*=17, 19, 21, 25, and 27, and *k*=21 provided the most stable balance between similarity-estimation accuracy and robustness. Larger *k* values showed increasing deviation from the expected values, likely because longer *k*-mers are more sensitive to sequencing errors and nucleotide variation. This trade-off is especially relevant for *Camellia* genomes, which are large, highly heterozygous, and repeat-rich. Shorter *k*-mers may increase chance matches in repetitive regions, whereas longer *k*-mers may reduce sensitivity because single-base differences disrupt the entire *k*-mer. Therefore, k=21 was retained as the MIKE-supported foundational setting for standardized CamK-DB fingerprint generation. In this study, a *k*-mer refers to a contiguous DNA sequence of length *k* extracted from sequencing reads named as MIKE-derived unique fingerprints. Thus, Cam*K*-DB compares compact standardized MIKE fingerprints. Briefly, after quality filtering and 5× coverage standardization, reads were decomposed into 21-mers. Identical 21-mers were deduplicated, and each 21-mer was divided into a 10-base prefix and an 11-base sufsfix. *K*-mers sharing the same 10-base prefix were grouped together, giving a fixed theoretical prefix space of 4^10= 1,048,576 possible groups. For each prefix group, MIKE records a minimized hash-derived value from the corresponding suffixes. The resulting `.jac` fingerprint is therefore a compact prefix-MinHash representation of the sample rather than a table of MIKE-derived fingerprint elements. Because the fingerprint

is represented in a standardized prefix feature space, the number of possible retained fingerprint elements is approximately fixed across samples, whereas sequencing-depth differences mainly affect which *k*-mers are recovered before minimization and therefore can influence similarity scores. To reduce this technical bias, all retained database accessions were standardized to 5× genome coverage before MIKE fingerprint generation Coverage-matching analysis showed that similarity was highest when query and database fingerprints were generated at the same coverage Fig. 2A, B.

2. Jaccard Similarity (MinHash Signatures): MIKE harnessed the MinHash algorithm to create a compact and exclusive MinHash signature for each biological sample of tea. These MinHash fingerprints were processed to calculate the Jaccard coefficient, which is the ratio of the count of shared to unique *k*-mers between two samples. MIKE used this approximate Jaccard coefficient to calculate the evolutionary distance (d) between samples using a formula derived from the Poisson distribution, assuming single-base substitutions.

The Jaccard similarity is calculated with the formula below:

$$Jacard\ Similarity = \frac{|S_1 \cap S_2|}{|S_1 \cup S_2|}$$

For two samples, S_1 and S_2 represent the sets of MIKE-derived fingerprint elements generated from the two read datasets. A shared element is counted only when both the prefix and the MinHash (suffix) value match exactly between the two samples. Mismatches are not allowed. Where S_1 denoted the *k*-mers set from sample 1, while S_2 denoted the *k*-mers set from sample 2. The MIKE-derived fingerprint construction and Jaccard comparison are summarized in Fig. 1.

Processing reads in MIKE

MIKE was developed to estimate sequence similarity based on *k*-mer frequency. We employed MIKE to enumerate all possible *k*-mers within a 2000-sketch size and then calculated the frequency of each *k*-mer. MinHash fingerprints were then derived from the *k*-mer representations of the lowest hash values. This signature fingerprint serves as a heuristic representation of a sequence, capturing its essential features. The resulting ".jac" files contained integer-count pairs encoding MinHash signatures, specific to each sample's signature, comprising up to 1,048,576 hash buckets to capture both the presence and frequency of hashed genomic elements (Fig 2).

Sketch-size optimization

To evaluate the effect of sketch size on similarity estimation, we tested sketch/pre_cnt values of 100, 500, 1000, 2000, 5000, 8000, and 10000. For each setting, similarity estimates from the sketched MIKE fingerprint were compared with the full fingerprint comparison, and RMSE was calculated across samples. Small sketch sizes produced higher RMSE, whereas increasing the sketch size reduced the error. The median RMSE became low and stable around sketch size 2000, while larger values provided little additional improvement. Runtime analysis showed that moderate sketch sizes had similar computational cost, whereas very large sketch sizes substantially increased query time. Therefore, sketch/pre_cnt = 2000 was selected as the recommended setting for the standardized Cam*K*-DB release Fig.S2 A, B.

Construction of the database, GUI, and basic features

Following data collection, the database schema was constructed using an in-house Python script (create_db.py), which processed two primary inputs: a tab-delimited metadata file containing sample identifiers (ID), nomenclature (Chinese and English names), taxonomic classification (species and group), geographic provenance (country and region), and a directory of". jac" files, each corresponding to a unique sample ID. The script generated an SQLite database file (Cam*K*-DB) designed to facilitate efficient fingerprint data retrieval through an SQLite-compatible graphical user interface (GUI). The schema featured a central tea_info table for storing dataset metadata, alongside individual tables for each sample organized by ID, which housed MinHash signatures and precomputed similarity strings. This architecture minimized reliance on cross-table joins during query filtering, thereby enhancing the computational efficiency for per-sample analytical operations, while maintaining structural clarity and scalability for downstream applications.

Effect of sequencing depth on fingerprint stability

Fingerprints were generated from accession CRR1173463 at 1×, 2×, 3×, 4×, 5×, 10×, 15×, and 20× genome coverage and compared across coverage levels Fig2A. Similarity values were highest when query and database fingerprints were generated at the same coverage, whereas mismatched coverage levels reduced similarity. This result supports the use of matched coverage during database construction and query processing.

Data Records

Cam*K*-DB is distributed as a single SQLite database file (TEA.db), which stores all derived MinHash fingerprints and sample-level metadata for the 418 *Camellia* accessions. The core data of Cam*K*-DB (~2.258 GB, (2,258,092,032 bytes)) consists of MinHash fingerprints derived from *k*-mers using Mike-generated ".jac" files that encode MinHash signatures as integer–count pairs. Each sample's signature includes up to 1,048,576 hash buckets, capturing the presence and frequency of the hashed elements. This approach enables the efficient representation and comparison of genomic features through MinHash-based similarity estimation. This database was designed for efficient storage and retrieval. It uses a master table (tea_info) containing general information about each tea tree sample (ID, name, classification, and origin). The tea_info table contains one record for each of the 418 Camellia accessions and includes seven fields: id, name, English name, species, groups, country, and area. The 14 group-specific tables correspond to the genetic groups CQ, CSA.G1, CSA.G1–CSS.G3, CSA.G2, CSS.G1, CSS.G1–G2, CSS.G2, CSS.G3, CSS.G4, CSS. Mixed, CS.CA, CT, CTR, and Out1 (Table S2). Group names are recorded using dot notation in the tea_info table, whereas underscore-compatible names are used for the corresponding SQLite tables. The TEA.db file, together with the complete Cam*K*-DB source code, sample data, and GUI resources, is archived at Zenodo [22].

Technical Validation

We evaluated the reliability, and biological interpretability of the standardized Cam*K*-DB release through a series of complementary validation analyses. These included accession-level self-identification, artificial two-source mixed-sample detection, concordance between Cam*K*-DB similarity and phylogenetic distance, out-of-database query assessment, sequencing-depth robustness, sketch-size sensitivity, and group-level representation.

Self-identification of accessions represented in Cam*K*-DB

As an internal consistency assessment, we first accessed whether Cam*K*-DB could correctly recover accessions already represented in the standardized TEA.db database. Ten accessions, including PT57B, SRR12265461, SRR12265466, Y12A, Y28A, Y30A, Y31A, Y38A, YN6A, and ZH5A, were selected as query samples. For each accession, resequencing reads (WGS) were queried against TEA.db. In all ten cases, the top-ranked database hit corresponded to the query accession itself (Table 1). Primary similarity values ranged from 0.544 to 0.607, with a mean of 0.583. By contrast, the second-ranked hits showed lower similarity values, ranging from 0.275 to 0.373, with a mean of 0.311. The mean top1–top2 similarity margin was 0.272, indicating clear separation between the matched accession and the closest non-identical database neighbor. These results demonstrate that, under the

standardized 5× fingerprinting strategy, Cam*K*-DB can reliably recover represented accessions and distinguish them from related but non-identical entries in the database.

Artificial two-source mixed-sample detection

We further evaluated whether Cam*K*-DB could recover known genomic contributors from artificial mixed-read datasets. Five database accessions were selected, and pairwise combinations of their resequencing reads were merged to generate ten two-source mixed datasets. The corresponding ".jac" fingerprints were generated and queried against TEA.db using the same standardized comparison framework. All ten artificial mixtures indicated two highest-ranked database records corresponded to the two accessions used to construct the mixed query dataset (Table 2). Across the ten mixtures, primary similarity values ranged from 0.386 to 0.504, with a mean of 0.434, whereas secondary similarity values ranged from 0.372 to 0.489, with a mean of 0.418. The mean top1–top2 margin was small, 0.015, consistent with the expectation that both source accessions contribute comparable genomic signals to the mixed-read fingerprint.

This analysis demonstrates that Cam*K*-DB can recover dual-source signals from artificial mixed-read datasets when both contributing accessions are represented in the database.

However, this *in silico* assessment should be interpreted as mixed-sample or two-source signal detection rather than formal biological parentage or hybrid-origin inference. A physical mixture of reads from two accessions does not recapitulate the genomic structure of a true biological hybrid, because hybrids inherit recombined haplotypes generated through meiosis and segregation. Therefore, formal inference of parentage, hybrid origin, or genealogical ancestry would require validated hybrid material, segregating progeny, or inheritance-aware analytical models.

Concordance with phylogenetic distance and out-of-database query behavior

To assess whether CamK-DB similarity reflected biologically meaningful genetic relationships, we compared the similarity scores with phylogenetic distances obtained from the previously published Camellia phylogenetic tree of Kong et al. [12]. Most accessions showed negative correlations between CamK-DB similarity and phylogenetic distance, indicating that genetically closer accessions generally exhibited higher fingerprint similarity (Fig. 2D). These results support the biological relevance of CamK-DB similarity scores. We further assessed Cam*K*-DB behavior using an out-of-database query sample, SRR11672521, not being in the standardized TEA.db release. Although the exact accession was absent from the database, higher Cam*K*-DB similarity was associated with lower phylogenetic distance to database accessions ($r = -0.37$, $P = 4.79 \times 10^{-15}$; Fig. 2C). This result indicates that, when an

exact database match is unavailable, CamK-DB can identify the closest available genomic relatives. Such results should be interpreted as nearest-neighbor relationships rather than definitive accession identification.

Sequencing-depth robustness and sketch-size sensitivity

Because MIKE-derived fingerprints depend on the subset of *k*-mer-derived elements recovered from sequencing reads, sequencing depth can influence the resulting fingerprint composition and Jaccard similarity estimates. We therefore evaluated the effect of coverage matching on fingerprint similarity. Coverage-comparison analysis showed that similarity was highest when query and database fingerprints were generated at matched genome coverage and decreased when coverage differed (Fig. 2A–B). This result supports the use of standardized 5× genome-coverage fingerprints for both database construction and query processing, thereby reducing technical bias caused by sequencing-depth heterogeneity.

We also evaluated the effect of sketch size on similarity-estimation accuracy and runtime. Sketch/pre_cnt values of 100, 500, 1000, 2000, 5000, 8000, and 10000 were tested by comparing sketched similarity estimates with full fingerprint comparisons. Small sketch sizes produced higher root mean square error (RMSE), whereas increasing the sketch size substantially reduced estimation error. The RMSE distribution reached a low and stable level around sketch/pre_cnt = 2000, while larger sketch sizes did not provide proportional improvement in accuracy. Runtime remained comparable among moderate sketch sizes but increased markedly at very large values. Together, these results support sketch/pre_cnt = 2000 as an appropriate balance between similarity-estimation accuracy and computational efficiency for standardized Cam*K*-DB queries (Fig. S2A–B).

Group-level representation of the standardized database

Finally, we evaluated the distribution of accessions across *Camellia* groups in the standardized 418-accession database. The retained accessions were distributed across multiple taxonomic and genetic groups, with a median group size of 16 accessions (Fig. S2C). This indicates that the standardized Cam*K*-DB release preserves broad group-level representation for reference-free accession comparison. Nevertheless, some groups remain less densely represented than others, and future database expansions should prioritize additional sampling of underrepresented groups to improve resolution across the full diversity of *Camellia* germplasm.

Methods

Source resequencing datasets and licensing

Cam*K*-DB integrates whole-genome resequencing data from 418 *Camellia* accessions. Newly generated data for 320 accessions were produced as part of a recent large-scale genomic survey of *Camellia* germplasm and are deposited in the GSA database [23] (BioProject, accession PRJCA026608 [12]). Previously published resequencing data for 415 accessions were downloaded from the GSA and NCBI SRA repositories [23, 25] under BioProjects PRJNA665594 [15] , and in the GSA database under the accession PRJCA003090 [15], PRJNA646044 [16], PRJNA716079 [17], and PRJNA597714 [18]. All of these datasets were obtained from repositories that explicitly permit reuse for research purposes. In Cam*K*-DB, we only redistribute derived MinHash fingerprints, the SQLite database file, and sample-level metadata; we do not redistribute the original raw reads from third-party studies, thereby complying with the terms and conditions of the original data providers (Table S1, S5).

Sequencing data processing

For the construction of database, this study utilized a dataset of 418 *Camellia* accessions, based on our recently published research on *Camellia's* evolutionary history [12]. This publication encompassed an expanded dataset that included 802 newly sequenced reads and 523 previously sequenced reads. *Camellia* accessions selected in this work originated from 13 countries: Azerbaijan, China, Georgia, Japan, Kenya, Laos, Myanmar, Sri Lanka, and the USA.

However, we used 320 newly sequenced accessions and 98 publicly available accessions (total n = 418) after QC for Cam*K*-DB. The dataset included diverse *Camellia* species: *C. oleifera*, *C. quinquelocularis*, *C. sasanqua*, *C. sinensis* var. *assamica*, *C. sinensis* var. *pubilimba*, *C. sinensis* var. *sinensis*, *C. tachangensis* var. *remotiserrata*, and *C. taliensis*. Importantly, the *C. sinensis* accessions represent a mix of wild, elite, and landrace varieties, whereas the remaining other species were included as wild samples. Among 418 selected accessions, we used 320 newly sequenced *Camellia* accessions from multiple countries, including Azerbaijan, China, Georgia, Iran, Laos, Myanmar, Sri Lanka, and Russia, from our sequenced resource (originally sequencing depth ranging between 9-23). Genomic DNA from the leaves was extracted using the DNeasy Plant Mini Kit (Qiagen), followed by 5 µg to construct libraries with an insert size of 500 bp. Each sample consisted of 150 bp paired-end reads on the DNBSEQ-T7 platform (Beijing Genomics Institute). Furthermore, to increase the sample diversity, resequencing data accessions of *Camellia* were downloaded from public databases i.e., National Genomics Data Center, [24] and National Center for Biotechnology Information, [25] from the given research [15–18,19]. Raw sequencing reads were down sampled to 5× subjected to quality control and removal of low-quality reads and adapter sequences by Trimmomatic with default parameters i.e., Trimmomatic PE -phred33 ILLUMINACLIP:adapters.fa:2:30:10 LEADING:3 TRAILING:3 SLIDINGWINDOW:4:15 MINLEN:50 [20]. The resulting high-quality reads

were processed using the MIKE tool to generate minimum hash signatures for each sample (Table S1). Across the 418 datasets, the mean percentages of Q20 and Q30 bases were 97.62% and 92.56%, respectively, while the mean GC content was 39.71%. The Q20, Q30, and GC-content values ranged from 92.95% to 99.12%, 85.48% to 96.83%, and 37.99% to 45.89%, respectively (Tables S3 and S4). The number of retained reads per accession ranged from approximately 50.39 million to 102.14 million, with an average of 100.44 million reads. The corresponding sequence yield was 15 Gb on an average per accession. Following adapter trimming and quality filtering, the high-quality reads were aligned to the chromosomal-scale *C. sinensis* TGY genome (~3.15 Gb) [15] by BWA [21] with default parameters. The alignment results demonstrated high sequence similarity and minimal contamination. Genome coverage was high across all samples, exceeding 88% on average, with several samples achieving a genome anchorage greater than 94% (Table S3, S4).

User guide and input data specifications

Cam*K*-DB provides both command-line and graphical interfaces for reference-free identification of *Camellia* accessions using MIKE-derived MinHash fingerprints. The current standardized release of Cam*K*-DB contains 418 *Camellia* accessions, each processed under the same 5× genome-coverage sampling strategy. This standardization is important because MIKE/Jaccard similarity can be affected by sequencing-depth differences between the database fingerprint and the query fingerprint. Therefore, query samples should be processed using the same 5× coverage-normalized workflow before comparison with the TEA.db database.

Input data

Cam*K*-DB accepts either raw sequencing files or precomputed MIKE fingerprint files. Supported input formats include FASTQ/FASTA files and MIKE-generated `.jac` files. The current release was validated primarily using short-read whole-genome resequencing data generated from Illumina-compatible or DNBSEQ paired-end platforms. For reliable comparison with the standardized TEA.db database, users should provide high-quality reads with sufficient usable depth to allow downsampling to 5× genome coverage. Query datasets with less than 5× usable coverage after quality filtering is not recommended for high-confidence identification.

Read preprocessing and coverage normalization

Before fingerprint generation, raw reads should be filtered to remove adapter contamination, low-quality bases, and short reads. In this study, reads were processed using quality-control steps equivalent to adapter trimming, low-quality base removal, sliding-window quality filtering, and exclusion of reads shorter than 50 bp. After quality filtering, query reads should

be downsampled to 5× genome coverage using the same genome-size estimate applied during database construction. This step is required because fingerprints generated at unmatched coverage levels may show reduced similarity even when they originate from the same accession.

MIKE fingerprint generation

After preprocessing and 5× downsampling, query reads are converted into MIKE `.jac` fingerprint files using the same parameters used for database construction. The *k*-mer length should be set to *k*=21, following the MIKE framework. The recommended sketch/pre_cnt value for Cam*K*-DB queries is 2000, based on sensitivity analysis showing a favorable balance between similarity-estimation accuracy and runtime. Query fingerprints should therefore be generated using *k*=21, 5× genome coverage, and sketch/pre_cnt=2000 to ensure consistency with the standardized TEA.db database.

Command-line query

The command-line interface is suitable for batch analysis and high-throughput screening. A typical query uses the TEA.db database, a query `.jac` file, and the recommended sketch/pre_cnt value:

`query_db TEA.db query_sample.jac 2000`

The program compares the query fingerprint against the standardized Cam*K*-DB database and returns the top-ranked accessions according to Jaccard similarity. The output table includes accession identifiers, names, taxonomic or genetic group information, geographic metadata, and similarity scores.

Graphical user interface

The graphical user interface provides an interactive workflow for users who prefer not to run command-line queries. Users first select the standardized TEA.db database and then provide either a precomputed `.jac` fingerprint or a processed query file for fingerprint generation. After running the query, the GUI displays the top-ranked database accessions with their metadata and similarity scores. Results can be sorted by similarity to identify the closest database matches Fig. S1.

Interpretation of query results

Cam*K*-DB should be interpreted as a nearest-neighbor genomic fingerprinting system. A high top-ranked similarity score together with a clear separation between the first and second hits supports a confident exact or near-clonal match, provided that the accession is represented in

the database. Moderate similarity may indicate a close relative or accession from the same genetic group. Low similarity or a small top1–top2 margin suggests that the query accession may be absent from the current database or poorly represented by available database accessions.

For samples whose exact identity or close relatives are not present in Cam*K*-DB, the top-ranked hits should not be interpreted as definitive identity assignments. Instead, they represent the closest available genomic neighbors within the standardized database. Users should therefore consider both the absolute similarity value and the top-hit margin when interpreting results.

Limitations

The current standardized Cam*K*-DB release contains 418 accessions processed under a matched 5× genome-coverage strategy. Query fingerprints generated at arbitrary or unmatched coverage should not be directly compared with the standardized database, because coverage differences can reduce Jaccard similarity. Formal biological parentage or hybrid-origin inference is not supported by the current release; mixed-read analyses should be interpreted only as dual-source or mixed-sample signal detection. Future releases may incorporate additional accessions and further optimize parameters for specific *Camellia* groups or sequencing platforms.

Supplementary information

Supplementary Figure S1. CamK-DB graphical user interface workflow.
Overview of the graphical workflow for selecting a query input, loading the standardized TEA.db database, processing or selecting a MIKE .jac fingerprint, performing the similarity search, and displaying the top-ranked accessions together with their identifiers, metadata, and Jaccard similarity values.

Supplementary Figure S2. Effects of sketch size on CamK-DB performance and group-level representation.
(A) Root mean square error between similarity estimates generated using different sketch/pre_cnt values and those obtained from the full fingerprint comparison. (B) Query runtime across the tested sketch/pre_cnt values. The combined accuracy and runtime analyses support sketch/pre_cnt = 2000 as the recommended setting for CamK-DB queries. (C) Distribution of the 418 standardized accessions among the 14 genetic

groups represented in CamK-DB. Group sizes ranged from 1 to 106 accessions, with a median of 16, demonstrating broad but uneven representation across groups.

Supplementary Table S1. Metadata and sequencing information for the *Camellia* accessions included in CamK-DB.

List of the 418 accessions used for database construction, including accession identifiers, names, taxonomic assignments, genetic-group classifications, geographic origins, sequencing-data sources, and associated database information.

Supplementary Table S2. Distribution of CamK-DB accessions among genetic groups and SQLite tables.

Summary of the 14 genetic groups represented in the standardized database, including the corresponding SQLite table names and the number and percentage of accessions assigned to each group.

Supplementary Table S3. Sequencing-read quality statistics for the 418 *Camellia* accessions.

Accession-level quality-control metrics, including the number of retained reads, total retained bases, Q20 and Q30 percentages, GC content, and estimated post-processing sequencing depth.

Supplementary Table S4. Reference-mapping and genome-coverage statistics for the 418 Camellia accessions.

Accession-level alignment statistics generated against the *C. sinensis* TGY reference genome, including mapping rate, mapped-read counts, genome-coverage breadth, and other relevant alignment-quality measurements.

Supplementary Table S5. List of Archive_Metadata, downloading links and relevant information

Author Contributions

Conceptualization: X.Z., F.W. and B.Z.; Methodology and algorithm development: F.W. and Noor; Database and GUI development: S.Z.; Validation and analysis: W.K., Y.S. and H.F.; Writing: Noor and F.W: Author B; Writing-review & editing: F.W. and X.Z.

Data Availability

The CamK-DB dataset, including the TEA.db SQLite database, per-sample MinHash fingerprint files, and the main program, is archived in Zenodo [22]. Newly generated raw whole-genome resequencing data are publicly available in the Genome Sequence Archive (GSA) [23] under BioProject accession PRJCA026608. Previously published resequencing data used to construct CamK-DB are available under BioProject accessions PRJNA665594, PRJCA003090, PRJNA646044, PRJNA716079, and PRJNA597714 through the GSA and NCBI Sequence Read Archive (SRA) [23,25].

Availability of Source Code and Requirements

Project name: CamK-DB

Project version: v1.0.1

Project home page: https://github.com/sc-zhang/CamK-DB

Operating system(s): Windows 10/11 and Linux (x86_64; glibc ≥ 2.27)

Programming language: C++17 and Python

Other requirements: SQLite3 and Qt 6; CMake ≥ 3.24 is required for source compilation. MIKE-generated .jac fingerprint files are required for CamK-DB queries and input samples should be down samples to 5x depth for authenticated comparisons.

License: MIT License

RRID: Not applicable

bio.tools ID: Not applicable

All software and analytical scripts developed for this study are publicly available under open-source licenses via GitHub repositories. Supporting software resources are available through the MIKE [26] and CamK-DB [27] GitHub repositories.

Competing Interests

The authors declare no competing interests.

Funding Information

This work was supported by National Natural Science Foundation of China grant (No. 32222019 to X.Z.) and Shenzhen Science and Technology Program (Grant No. RCYX20210706092103024 to X.Z.), the Open Fund of Collaborative Innovation Center of Chinese Oolong Tea Industry (2025W 01), the Project of Science and Technology Plan of Nanping city (N2024Z007), the Project of Science and Technology Plan of Fujian Province (2023N5013, 2024N3013), the Scientific Research Fund of Wuyi University (2018N2004, 2021L3058), the Special Funds for Colleges and Universities in Fujian Province to Improve the Level of Running Schools (No.77 [2022]).

References

1. Zhang ZB, Xiong T, Chen JH, Ye F, Cao JJ, Chen YR, et al. Understanding the Origin and Evolution of Tea (*Camellia sinensis* [L.]): Genomic Advances in Tea. *J Mol Evol*. 2023;91:156–168. http://dx.doi.org/10.1007/s00239-023-10099-z
2. Shen Y, He X, Zu F, Huang X, Yin S, Wang L, et al. Development of Genome-Wide Intron Length Polymorphism (ILP) Markers in Tea Plant (*Camellia sinensis*) and Related Applications for Genetics Research. *Int J Mol Sci*. 2024;25:3241. http://dx.doi.org/10.3390/ijms25063241
3. Wang X, Xin B, Guo M, Yu G, Wang J. GWASTool: A web pipeline for detecting SNP-phenotype associations. *Fundam Res*. 2024;4:761–769. http://dx.doi.org/10.1016/j.fmre.2024.03.005
4. Baird NA, Etter PD, Atwood TS, Currey MC, Shiver AL, Lewis ZA, et al. Rapid SNP Discovery and Genetic Mapping Using Sequenced RAD Markers. *PLoS One*. 2008;3:e3376. http://dx.doi.org/10.1371/journal.pone.0003376
5. Elshire RJ, Glaubitz JC, Sun Q, Poland JA, Kawamoto K, Buckler ES, et al. A Robust, Simple Genotyping-by-Sequencing (GBS) Approach for High Diversity Species. *PLoS One*. 2011;6:e19379. http://dx.doi.org/10.1371/journal.pone.0019379
6. Boratyn GM, Camacho C, Cooper PS, Coulouris G, Fong A, Ma N, et al. BLAST: a more efficient report with usability improvements. *Nucleic Acids Res*. 2013;41:W29–W33. http://dx.doi.org/10.1093/nar/gkt282
7. Liu S, Koslicki D. CMash: fast, multi-resolution estimation of k-mer-based Jaccard and containment indices. *Bioinformatics*. 2022;38:i28–i35. http://dx.doi.org/10.1093/bioinformatics/btac237
8. Wang F, Wang Y, Zeng X, Zhang S, Yu J, Li D, et al. MIKE: an ultrafast, assembly-, and alignment-free approach for phylogenetic tree construction. *Bioinformatics*. 2024;40:btae154. http://dx.doi.org/10.1093/bioinformatics/btae154
9. Lin P, Wang K, Wang Y, Hu Z, Yan C, Huang H, et al. The genome of oil-Camellia and population genomics analysis provide insights into seed oil domestication. *Genome Biol*. 2022;23:14. http://dx.doi.org/10.1186/s13059-021-02599-2
10. Zhang L, Shi Y, Gong W, Zhao G, Xiao S, Lin H, et al. The tetraploid *Camellia oleifera* genome provides insights into evolution, agronomic traits, and genetic architecture of oil Camellia plants. *Cell Rep*. 2024;43:114902. http://dx.doi.org/10.1016/j.celrep.2024.114902
11. Gong W, Xiao S, Wang L, Liao Z, Chang Y, Mo W, et al. Chromosome-level genome of *Camellia lanceoleosa* provides a valuable resource for understanding

genome evolution and self-incompatibility. *Plant J*. 2022;110:881–898. http://dx.doi.org/10.1111/tpj.15739

12. Kong W, Kong X, Xia Z, Li X, Wang F, Shan R, et al. Genomic analysis of 1,325 *Camellia* accessions sheds light on agronomic and metabolic traits for tea plant improvement. *Nat Genet*. 2025;57:997–1007. http://dx.doi.org/10.1038/s41588-025-02135-z
13. An Y, Zhang X, Jiang S, Zhao J, Zhang F. TeaPVs: a comprehensive genomic variation database for tea plant (*Camellia sinensis*). *BMC Plant Biol*. 2022;22:513. http://dx.doi.org/10.1186/s12870-022-03901-5
14. Chen JD, He WZ, Chen S, Chen QY, Ma JQ, Jin JQ, et al. TeaGVD: A comprehensive database of genomic variations for uncovering the genetic architecture of metabolic traits in tea plants. *Front Plant Sci*. 2022;13:1056891. http://dx.doi.org/10.3389/fpls.2022.1056891
15. Zhang X, Chen S, Shi L, Gong D, Zhang S, Zhao Q, et al. Haplotype-resolved genome assembly provides insights into evolutionary history of the tea plant *Camellia sinensis*. *Nat Genet*. 2021;53:1250–1259. http://dx.doi.org/10.1038/s41588-021-00895-y
16. Wang X, Feng H, Chang Y, Ma C, Wang L, Hao X, et al. Population sequencing enhances understanding of tea plant evolution. *Nat Commun*. 2020;11:4447. http://dx.doi.org/10.1038/s41467-020-18228-8
17. Lu L, Chen H, Wang X, Zhao Y, Yao X, Xiong B, et al. Genome-level diversification of eight ancient tea populations in the Guizhou and Yunnan regions identifies candidate genes for core agronomic traits. *Hortic Res*. 2021;8:190. http://dx.doi.org/10.1038/s41438-021-00617-9
18. Xia E, Tong W, Hou Y, An Y, Chen L, Wu Q, et al. The Reference Genome of Tea Plant and Resequencing of 81 Diverse Accessions Provide Insights into Its Genome Evolution and Adaptation. *Mol Plant*. 2020;13:1013–1026. http://dx.doi.org/10.1016/j.molp.2020.04.010
19. Lei Y, Yang L, Duan S, Ning S, Li D, Wang Z, et al. Whole-genome resequencing reveals the origin of tea in Lincang. *Front Plant Sci*. 2022;13:984422. http://dx.doi.org/10.3389/fpls.2022.984422
20. Bolger AM, Lohse M, Usadel B. Trimmomatic: a flexible trimmer for Illumina sequence data. *Bioinformatics*. 2014;30:2114–2120. http://dx.doi.org/10.1093/bioinformatics/btu170
21. Li H, Durbin R. Fast and accurate short read alignment with Burrows–Wheeler transform. *Bioinformatics*. 2009;25:1754–1760. http://dx.doi.org/10.1093/bioinformatics/btp324
22. Zhang S. sc-zhang/CamK-DB [Database].2026.CamK-DB. Zenodo. http://dx.doi.org/10.5281/zenodo.21204832
23. Genome Sequence Archive (GSA). National Genomics Data Center, China National Center for Bioinformation. https://ngdc.cncb.ac.cn/gsa/. Accessed 14 Jan 2025.
24. National Genomics Data Center (NGDC), China National Center for Bioinformation. https://ngdc.cncb.ac.cn/. Accessed 11 Feb 2025.
25. NCBI Sequence Read Archive (SRA). National Center for Biotechnology Information. https://www.ncbi.nlm.nih.gov/sra/. Accessed 17 Aug 2025.
26. MIKE toolkit. GitHub repository. https://github.com/Argonum-Clever2/mike. Accessed 22May 2025.
27. CamK-DB (version 1.0.1). GitHub repository. https://github.com/sc-zhang/CamK-DB. Accessed 4 June 2025.

Figure Captions

Figure 1. MIKE-based fingerprint generation and Jaccard similarity calculation. Quality-filtered reads are downsampled to 5× genome coverage and decomposed into 21-mers. Identical 21-mers are deduplicated, and each 21-mer is divided into a 10-bp prefix and an 11-bp suffix. Similar prefix sharing *k*-mers are grouped, and MIKE records a minimized hash-derived value from the corresponding suffix group. The resulting (prefix, MinHash (suffix)) elements are stored as ".jac" fingerprints and compared using Jaccard similarity. Query samples are processed using the same k=21 and sketch/pre_cnt=2000 settings before comparison with TEA.db.

Figure 2. Validation of coverage standardization and biological relevance of CamK-DB similarity.

(A) Effect of matched and unmatched sequencing coverage on MIKE fingerprint similarity for accession CRR1173463. (B) Random-subsampling analysis showing the effect of query subsampling level and database fingerprint coverage on similarity.

(C) Relationship between Cam*K*-DB similarity and phylogenetic distance for the out-of-database accession SRR11672521. Phylogenetic distances were obtained from the published Camellia phylogenetic tree reported by Kong et al. [12].

(D) Distribution of Pearson correlation coefficients between Cam*K*-DB similarity and published phylogenetic distances. Negative values indicate that higher fingerprint similarity generally corresponds to shorter phylogenetic distance.

Figure 3. Architecture of the standardized Cam*K*-DB database. Cam*K*-DB contains 418 standardized *Camellia accessions* represented by MIKE-derived MinHash fingerprints generated at 5× genome coverage. The SQLite database includes a central tea_info metadata table linked to group-specific fingerprint tables. The fingerprint field stores encoded MIKE prefix-MinHash features used for Jaccard similarity calculation, rather than precomputed pairwise similarity values.

Figure S1. Cam*K*-DB graphical user interface (GUI) workflow for varietal identification (steps 1-6). Pictures are arranged from top-to-bottom. Step 1 - Choose query input. Select either a local FASTQ/FASTA file or an existing sample from the database, designated as the query. Step 2 automates database detection or prompts for creating "Tea.db" file and GUI interface provides a selection bar for the following step. Step 3- Open ".MinHash.jac "file for processing. Step 5 - Run similarity search and inspect results. Launch the search to obtain nearest neighbors. Step 6 - The results table shows each candidate's sample ID, name, and Jaccard similarity (higher is closer).

Figure S2. Effect of sketch size on Cam*K*-DB accuracy and runtime.

(A) RMSE between sketched similarity estimates and full fingerprint comparison across sketch sizes. RMSE decreased with increasing sketch size and stabilized around 2000. (B) Runtime across sketch sizes. Query time remained low at moderate sketch sizes but increased sharply at very large values. Together, these results support sketch/pre_cnt = 2000 as a suitable balance between similarity-estimation accuracy and computational efficiency. (C) . The database includes multiple taxonomic and genetic groups, with a median group size of 16 accessions, indicating higher genetic diversity. The open database-construction source codes also allow users to incorporate additional accessions and generate customized Cam*K*-DB databases for specific germplasm collections.

Table 1. Self-identification performance of standardized Cam*K*-DB for accessions represented in TEA.db. Ten accessions represented in the standardized database were queried against TEA.db using the same 5× MIKE fingerprint-generation workflow. In all cases, the top-ranked hit corresponded to the same accession as the query sample.

Sample	Primary match		Secondary match	
	**ID**	**Similarity**	**ID**	**Similarity**
**PT57B**	PT57B	0.573086	PT44B	0.275166
**SRR12265461**	SRR12265461	0.605142	K78A	0.301827
**SRR12265466**	SRR12265466	0.59301	K78A	0.311545
**Y12A**	Y12A	0.559994	K47A	0.318512
**Y28A**	Y28A	0.604648	Y15A	0.372634
**Y30A**	Y30A	0.603477	K78A	0.314028
**Y31A**	Y31A	0.579548	K47A	0.28647
**Y38A**	Y38A	0.607403	K78A	0.309742
**YN6A**	YN6A	0.543805	YN4A	0.33486
**ZH5A**	ZH5A	0.559406	K78A	0.289278

Table 2. Cam*K*-DB query results for artificial two-source mixed-read datasets.

Artificial mixed-read datasets were generated by combining reads from pairs of accessions represented in the standardized Cam*K*-DB database. For all ten mixtures, the two highest-ranked database records corresponded to the two source accessions used to generate the query dataset. This result demonstrates that Cam*K*-DB can recover dual-source signals from artificial mixed-read data when both source accessions are present in the database. This analysis should be interpreted as mixed-sample signal detection and not as formal biological parentage or hybrid-origin inference.

Sample1	***Sample2***	Primary match		Secondary match	
		**ID**	**Similarity**	**ID**	**Similarity**
**Y12A**	PT57B	Y12A	0.385688	PT57B	0.377445
**Y12A**	Y28A	Y28A	0.500619	Y12A	0.481872
**Y12A**	Y38A	Y38A	0.504148	Y12A	0.482033
**Y12A**	YN6A	Y12A	0.414585	YN6A	0.410418
**Y28A**	PT57B	Y28A	0.39659	PT57B	0.373197
**Y28A**	Y38A	Y38A	0.492228	Y28A	0.488699
**Y28A**	YN6A	Y28A	0.426494	YN6A	0.406801
**Y38A**	PT57B	Y38A	0.398635	PT57B	0.371728
**Y38A**	YN6A	Y38A	0.427172	YN6A	0.403255
**YN6A**	PT57B	YN6A	0.389391	PT57B	0.385629

MS-track changes

Click here to access/download;Manuscript;Article-Final - Trackchanges.pdf

Cam*K*-DB: A *k*-mer MinHash fingerprint database for reference-free genotyping of *Camellia* accessions

Feiquan Wang[1,#], Noor-ul-Áin[2,#], Fang Wang[2,#], Shengcheng Zhang[2,#], Weilong Kong[2], Yutao Shi[1], Hua Feng[1], Bo Zhang[1,*], Xingtan Zhang[2,*]

[1] College of Tea and Food Sciences, Collaborative Innovation Center of Chinese Oolong Tea Industry, Wuyi University, Tea Engineering Research Center of Fujian Higher Education, Wuyishan Fujian, 354300, China

[2] State Key Laboratory of Tropical Crop Breeding, Shenzhen Branch, Guangdong Laboratory for Lingnan Modern Agriculture, Genome Analysis Laboratory of the Ministry of Agriculture and Rural Affairs, Agricultural Genomics Institute at Shenzhen, Chinese Academy of Agricultural Sciences, Shenzhen, 518120, China

# These authors contributed equally

* ~~Correspondance~~Correspondence to Xingtan Zhang (zhangxingtan@caas.cn) and Bo Zhang (Zhangbo@wuyiu.edu.cn)

Abstract

Tea (*Camellia sinensis* L.), a major global economic crop in Asia, poses challenges for genetic identification because its highly heterozygous, repetitive genome reduces the efficacy of conventional single-nucleotide polymorphism (SNP) and microsatellite markers, and interspecific hybridization further complicates the situation. ~~Interspecific hybridization further blurs genetic boundaries, promotes adulteration, and compromises breeding programs and commercial supply chains.~~ To address these issues, ~~we developed Cam*K*-DB, a DNA fingerprinting database employing the MinHash-based *k*-mer algorithm (MIKE).~~ CamK-DB was developed as a reference-free *Camellia* fingerprinting database built on MIKE MinHash sketches. We curated 418 candidate resequencing datasets, and built a database using standardized 5× genome-coverage fingerprints. Each accession is stored as a MIKE .jac fingerprint generated with k=21 and recommended sketch/pre_cnt=2000. ~~The repository integrates genomic signatures from 735 tea accessions spanning diverse geographical origins and phenotypic characteristics, derived from whole-genome resequencing data. Each accession is represented by compact MinHash fingerprints (*k*-mer size = 21, sketch size = 1000 hash values per genome) stored in a standardized SQLite relational database with associated~~

~~taxonomic metadata.~~ Cam*K*-DB provides a command-line interface for data management and a custom C++ query engine that computes top-10 matches using Jaccard similarity, complemented by a QT-based graphical interface for interactive analysis. This resource offers a robust and scalable framework for precise and routine germplasm identification, genomic phylogenetic inference, and strategic breeding program design.

Availability and implementation: Cam*K*-DB (database and code) is publicly available at (https://github.com/sc-zhang/CamK-DB). Cam*K*-DB binaries are provided for Windows 10/11 and Linux (x86_64, glibc ⩾ 2.27)

Keywords: MinHash; *k*-mer, *Camellia sinensis*, alignment-free genomics, Jaccard similarity, germplasm identification, SQLite database

Background

Tea plants (*Camellia sinensis*), famous for their outstanding foliage, are among the world's most cherished non-alcoholic beverages. Beyond its scope as a beverage crop, the tea industry extends to different sectors, such as finished products, tea ware, and global expansions. Technically, *C. sinensis* (L.) is the principal cultivated species for tea production, sectioned as *C. sinensis* var. *sinensis* (small-leaf tea) and *C. sinensis* var. *assamica* (large-leaf tea). Additionally, several wild relatives, including *C. taliensis*, *C. irrawadiensis*, *C. tachangensis*, and *C. grandibracteata*, account for the genetic diversity in China. Tea leaves face many challenges in phenotypic characterization due to their dense foliage, architecture, and phenotypic similarity. Additionally, tea plants exhibit complex genomic features, including high heterozygosity (>2%), frequent interspecific hybridization, extensive vegetative propagation, and a genome dominated by repetitive sequences such as long terminal repeat (LTR) retrotransposons (approximately 70% of the genome)[1]. These characteristics complicate traditional genetic analysis and cultivar identification. Thus, mislabeling and fraudulent substitution of tea varieties undermine consumer trust and economic stability, necessitating a robust anti-counterfeiting measure for the rapid and accurate identification of tea varieties.

Current methods for tea variety identification, such as morphological analysis and chemical profiling, are often time-consuming and lack the precision to distinguish closely related species and varieties [2,3]. Conventional methods of DNA fingerprinting

relied on molecular markers (e.g., SSRs/microsatellites, Amplified fragment length polymorphism ~~AFLPs~~), allowing cultivar identification and population genetics, but showed limited genome-wide resolution. Reduced representation approaches such as RAD-seq and genotyping-by-sequencing (GBS) based on short reads matured into alignment-based pipelines that infer SNPs against a reference genome [4,5]. Molecular markers, such as Simple Sequence Repeats (SSRs) and Single Nucleotide Polymorphisms (SNPs), are accurate and efficient but ~~pose high costs and complexity in genotyping~~ entail high genotyping costs and complexity [2,3]. These methods benefited from multiple-sequence-alignment tools (e.g., BLAST, ClustalW) that remain foundational and widely used [6]. Recently, *k*-mer-based analytical approaches have been extensively utilized in computational biology; however, the determination of an optimal *k*-mer length remains contingent upon heuristic optimization methodologies. Several hashing-based techniques, such as MinHash, Bloom filter, and Count-Min Sketch [7], have been developed or adopted ~~to efficiently compute *k*-mer-based similarity methods~~to compute k-mer-based similarity methods efficiently. MinHash efficiently estimates genetic similarity by representing genomes as compact sketches, thereby significantly reducing the computational demands, rather than to perform membership testing or frequency estimation. Although Bloom filters are highly memory-efficient and useful for filtering or indexing large *k*-mer collections, they do not directly provide an unbiased estimate of set similarity; false positives may inflate the apparent overlap between two samples unless additional correction or processing is applied. Similarly, Count-Min Sketch is primarily designed for approximate frequency estimation in streaming data and is therefore more appropriate for abundance-related queries than for direct set similarity comparisons.~~.~~ Inspired by these advancements, we previously developed the MinHash-based *k*-mer algorithm MIKE, tailored to process extensive genomic data, generate compact genetic signatures, and enable rapid similarity calculations [8]. Accordingly, CamK-DB adopts the MIKE MinHash framework for reference-free generation and comparison of compact genomic fingerprints.

Tea genomics has recently been endowed with two chromosome-scale assemblies of *C. lanceoleosa* and wild oil-*Camellia* and a haplotype-resolved tetraploid *C. oleifera*

genome, providing valuable insights into genome evolution, population structure, self-incompatibility, and agronomic traits [9–12]. Owing to the availability of large-scale genomic datasets, there is a dire need for a centralized and standardized repository that can facilitate the identification of different accessions and varieties irrespective of phenotypic and chemical methods. Many recent studies have contributed to the compilation of valuable datasets in various aspects of tea, such as an atlas of genomic variation, metabolites of tea cultivars, or aspects of tea plant biology [13,14]. Despite these recent advances, a publicly available *k*-mer fingerprint database for identifying tea accessions is still lacking, and Cam*K*-DB has been developed to address this gap. This alignment-free approach compares samples directly via their *k*-mer content, avoiding read mapping and multiple alignment. MinHash sketches transform large datasets into compact "signatures," enabling rapid, accurate similarity queries with small storage footprints. As it helps in (i) reference-free operation, (ii) good performance at low-to-moderate coverage, (iii) fast search across many accessions, and (iv) easy sharing/indexing of compact sketches. Benefitting from this, we created a *k*-mer based, low-cost, user-friendly database for germplasm identification. The base dataset is a spatially diverse resource of ~~735~~ 418 accessions of different species of *Camellia*. Built upon QT architecture and SQLite, the database enables rapid identification of tea tree varieties, offering unprecedented accuracy and efficiency in cultivar authentication, hybrid detection, and evolutionary research.

Theory behind Database Construction

Database construction was based on two key elements.

1. DNA Markers (*k*-mers): The foundation of the database lies in the genetic information obtained from ~~735~~ 418 accessions of tea resequencing data~~. We used an~~ followed by using an alignment-free and more accurate approach, i.e., *k*-mer similarity for building a database.~~, by creating substrings of length k = 21 in MIKE.~~ These fingerprints were generated using *k*=21 based on our previously developed method MIKE, which employed rigorous evaluation with 40× sequence coverage using various k=17, 19, 21, 25, and 27, and k=21 provided the most stable balance between similarity-estimation accuracy and robustness. Larger k values showed increasing deviation from the expected values, likely because longer k-mers are more sensitive to sequencing errors and nucleotide variation. This trade-off is especially relevant for *Camellia* genomes, which

are large, highly heterozygous, and repeat-rich. Shorter *k*-mers may increase chance matches in repetitive regions, whereas longer *k*-mers may reduce sensitivity because single-base differences disrupt the entire *k*-mer. Therefore, k=21 was retained as the MIKE-supported foundational setting for standardized CamK-DB fingerprint generation. ~~We used the algorithm's default and recommended setting k=21 to generate the fingerprint. Real *k*-mers, which are short DNA sequences of length *k* that appear in a single location (e.g., at a single genomic position, in a single species from a larger set of species), are called "*unique k-mers*" [15]. We then created MinHash fingerprints of these unique k-mers by generating prefixes and suffixes and creating a one-hot vector. The process was extended to create an index of nonzero elements, followed by a permutation to reduce dimensionality and estimate genetic similarity~~In this study, a *k*-mer refers to a contiguous DNA sequence of length *k* extracted from sequencing reads named as MIKE-derived unique fingerprints. Thus, Cam*K*-DB compares compact standardized MIKE fingerprints. Briefly, after quality filtering and 5× coverage standardization, reads were decomposed into 21-mers. Identical 21-mers were deduplicated, and each 21-mer was divided into a 10-base prefix and an 11-base suffix. *K*-mers sharing the same 10-base prefix were grouped together, giving a fixed theoretical prefix space of 4^10= 1,048,576 possible groups. For each prefix group, MIKE records a minimized hash-derived value from the corresponding suffixes. The resulting `.jac` fingerprint is therefore a compact prefix-MinHash representation of the sample rather than a table of MIKE-derived fingerprint elements. Because the fingerprint is represented in a standardized prefix feature space, the number of possible retained fingerprint elements is approximately fixed across samples, whereas sequencing-depth differences mainly affect which *k*-mers are recovered before minimization and therefore can influence similarity scores. To reduce this technical bias, all retained database accessions were standardized to 5× genome coverage before MIKE fingerprint generation Coverage-matching analysis showed that similarity was highest when query and database fingerprints were generated at the same coverage Fig. 2A-B.

2. Jaccard Similarity (MinHash Signatures): MIKE harnessed the MinHash algorithm to create a compact and exclusive MinHash signature for each biological sample of tea. These MinHash fingerprints were processed to calculate the Jaccard coefficient, which is the ratio of the count of shared to unique *k*-mers between two samples. MIKE used this

approximate Jaccard coefficient to calculate the evolutionary distance (d) between samples using a formula derived from the Poisson distribution, assuming single-base substitutions ~~(Figure 1)~~.

The Jaccard ~~Similarity~~ similarity is calculated with the formula below:

$$Jacard\ Similarity = \frac{|S_1 \cap S_2|}{|S_1 \cup S_2|}$$

~~Where S_1 denoted the *k*-mers set from sample 1, while S_2 denoted the *k*-mers set from sample2.~~

For two samples, S_1 and S_2 represent the sets of MIKE-derived fingerprint elements generated from the two read datasets. A shared element is counted only when both the prefix and the MinHash (suffix) value match exactly between the two samples. Mismatches are not allowed. Where S_1 denoted the *k*-mers set from sample 1, while S_2 denoted the *k*-mers set from sample 2. The MIKE-derived fingerprint construction and Jaccard comparison are summarized in Fig. 1.

Processing reads in MIKE

MIKE was developed to estimate sequence similarity based on *k*-mer frequency. We employed MIKE to enumerate all possible *k*-mers within a ~~1000~~2000-sketch size and then calculated the frequency of each *k*-mer. MinHash fingerprints were then derived from the *k*-mer representations of the lowest hash values. This signature fingerprint serves as a heuristic representation of a sequence, capturing its essential features. The resulting ".jac" files contained integer-count pairs encoding MinHash signatures, specific to each sample's signature, comprising up to 1,048,576 hash buckets to capture both the presence and frequency of hashed genomic elements (Fig~~ure~~ 2).

Sketch-size optimization

To evaluate the effect of sketch size on similarity estimation, we tested sketch/pre_cnt values of 100, 500, 1000, 2000, 5000, 8000, and 10000. For each setting, similarity estimates from the sketched MIKE fingerprint were compared with the full fingerprint comparison, and RMSE was calculated across samples. Small sketch sizes produced higher RMSE, whereas increasing the sketch size reduced the error. The median RMSE became low and stable around sketch size 2000, while larger values provided little

additional improvement. Runtime analysis showed that moderate sketch sizes had similar computational cost, whereas very large sketch sizes substantially increased query time. Therefore, sketch/pre_cnt = 2000 was selected as the recommended setting for the standardized Cam*K*-DB release Fig.S2 A, B.

Construction of the database, GUI, and basic features

Following data collection, the database schema was constructed using an in-house Python script (create_db.py), which processed two primary inputs: a tab-delimited metadata file containing sample identifiers (ID), nomenclature (Chinese and English names), taxonomic classification (species and group), geographic provenance (country and region), and a directory of". jac" files, each corresponding to a unique sample ID. The script generated an SQLite database file (Cam*K*-*K*-DB) designed to facilitate efficient fingerprint data retrieval through an SQLite-compatible graphical user interface (GUI). The schema featured a central tea_info table for storing dataset metadata, alongside individual tables for each sample organized by ID, which housed MinHash signatures and precomputed similarity strings. This architecture minimized reliance on cross-table joins during query filtering, thereby enhancing the computational efficiency for per-sample analytical operations, while maintaining structural clarity and scalability for downstream applications.

The core data of Cam*K*-DB (~2.33.9 GB, (2,258,092,0323,901,136,896 bytes)) consists of MinHash fingerprints derived from unique *k*-mers using Mike-generated ".jac" files that encode MinHash signatures as integer–count pairs. Each sample's signature includes up to 1,048,576 hash buckets, capturing the presence and frequency of the hashed elements. This approach enables the efficient representation and comparison of genomic features through MinHash-based similarity estimation. This database was designed for efficient storage and retrieval. It uses a master table (tea_info) containing general information about each tea tree sample (ID, name, classification, and origin). To reduce storage space and improve query speed, the database was divided into 14 sub-tables (one master table and 13 grouping tables), each storing the MinHash signatures for a specific group of tea trees. This allows faster searches within particular groups. The master table and sub-tables were linked using a tea tree ID as a foreign key. This database design was optimized for fast searching and comparison of the MinHash signatures (Figure 3).***Effect of sequencing depth on fingerprint stability***

Fingerprints were generated from accession CRR1173463 at 1×, 2×, 3×, 4×, 5×, 10×, 15×, and 20× genome coverage and compared across coverage levels Fig2A. Similarity values were highest when query and database fingerprints were generated at the same coverage, whereas mismatched coverage levels reduced similarity. This result supports the use of matched coverage during database construction and query processing.

Data Records

Cam~~K~~K-DB is distributed as a single SQLite database file (TEA.db), which stores all derived MinHash fingerprints and sample-level metadata for the ~~735~~ 418 *Camellia* accessions. The core data of Cam*K*-DB (~2.258 GB, (2,258,092,032 bytes)) consists of MinHash fingerprints derived from *k*-mers using Mike-generated ".jac" files that encode MinHash signatures as integer–count pairs. Each sample's signature includes up to 1,048,576 hash buckets, capturing the presence and frequency of the hashed elements. This approach enables the efficient representation and comparison of genomic features through MinHash-based similarity estimation. This database was designed for efficient storage and retrieval. It uses a master table (tea_info) containing general information about each tea tree sample (ID, name, classification, and origin). The tea_info table contains one record for each of the 418 Camellia accessions and includes seven fields: id, name, English name, species, groups, country, and area. The 14 group-specific tables correspond to the genetic groups CQ, CSA.G1, CSA.G1–CSS.G3, CSA.G2, CSS.G1, CSS.G1–G2, CSS.G2, CSS.G3, CSS.G4, CSS. Mixed, CS.CA, CT, CTR, and Out1 (Table S2). Group names are recorded using dot notation in the tea_info table, whereas underscore-compatible names are used for the corresponding SQLite tables. ~~The central table, tea_info, contains one row per accession and includes fields such as sample ID, cultivar names, species, variety or group, country and region of origin, and other basic metadata. For each logical group (for example species, region, or other user-defined subset), an additional table was created with three fields: id, name, and similarity. The id field is the primary key (PK) of the group table and, at the same time, acts as a foreign key referencing the corresponding accession in tea_info. The similarity field stores the MinHash *k*-mer sketch generated by MIKE and is used to compute the Jaccard's similarity between each stored sample and a query.~~ The TEA.db file, together with the complete Cam~~K~~K-DB source code, sample data, and GUI resources, is archived at Zenodo

(https://zenodo.org/records/21204832/files/TEA.db?download=1~~DOI: 10.5281/zenodo.15588935~~).

Technical Validation

~~To assess the self-identification performance of Cam*K*-DB, we randomly selected seven accessions (C5A, ZH-1A, SRR12322236, SRR12322243, SRR12333999, SRR12337253, and SRR12337254) that were already present in the database. For each accession, we downloaded the resequencing data and constructed the corresponding ".jac" file. When these files were used as queries against Cam*K*-DB, the primary hit was always the same accession as the query sample, with Jaccard similarity values ranging from 0.961 to 0.998 (mean = 0.975; Table 1). Contrary~~On the contrary~~, the secondary matches showed markedly lower similarity, ranging from 0.295 to 0.605 (mean = 0.389). These results show that Cam*K*-DB can distinguish identical from non-identical samples and can be used to determine whether a newly sequenced sample is already contained in the database. Table 1.~~

~~To validate whether Cam*K*-DB can be used to identify parental accessions of a sample when both parents are contained in Cam*K*-DB, we randomly selected 5 accessions from Cam*K*-DB, and down-sampled their resequencing data to 10x coverage. For every pair, we combined their down-sampled reads to generate mixed two-sample resequencing datasets and constructed the ".jac" file. The two database records with the highest similarity scores always corresponded to the two constituent parental accessions (Table 2). Across all ten two-sample mixtures, the primary similarity values ranged from 0.509 to 0.578 (mean ≈ 0.532), and the corresponding secondary similarity values ranged from 0.488 to 0.554 (mean ≈ 0.510). These results indicate that Cam*K*-DB can be used to trace recent ancestry and to infer parental accessions of a sample, provided that the parental lines are represented in the database.~~We evaluated the reliability, and biological interpretability of the standardized Cam*K*-DB release through a series of complementary validation analyses. These included accession-level self-identification, artificial two-source mixed-sample detection, concordance between Cam*K*-DB similarity and phylogenetic distance, out-of-database query assessment, sequencing-depth robustness, sketch-size sensitivity, and group-level representation.

Self-identification of accessions represented in Cam*K*-DB

As an internal consistency assessment, we first accessed whether Cam*K*-DB could correctly recover accessions already represented in the standardized TEA.db database.

Ten accessions, including PT57B, SRR12265461, SRR12265466, Y12A, Y28A, Y30A, Y31A, Y38A, YN6A, and ZH5A, were selected as query samples. For each accession, resequencing reads (WGS) were queried against TEA.db. In all ten cases, the top-ranked database hit corresponded to the query accession itself (Table 1). Primary similarity values ranged from 0.544 to 0.607, with a mean of 0.583. By contrast, the second-ranked hits showed lower similarity values, ranging from 0.275 to 0.373, with a mean of 0.311. The mean top1–top2 similarity margin was 0.272, indicating clear separation between the matched accession and the closest non-identical database neighbor. These results demonstrate that, under the standardized 5× fingerprinting strategy, Cam*K*-DB can reliably recover represented accessions and distinguish them from related but non-identical entries in the database.

Artificial two-source mixed-sample detection

We further evaluated whether Cam*K*-DB could recover known genomic contributors from artificial mixed-read datasets. Five database accessions were selected, and pairwise combinations of their resequencing reads were merged to generate ten two-source mixed datasets. The corresponding ".jac" fingerprints were generated and queried against TEA.db using the same standardized comparison framework. All ten artificial mixtures indicated two highest-ranked database records corresponded to the two accessions used to construct the mixed query dataset (Table 2). Across the ten mixtures, primary similarity values ranged from 0.386 to 0.504, with a mean of 0.434, whereas secondary similarity values ranged from 0.372 to 0.489, with a mean of 0.418. The mean top1–top2 margin was small, 0.015, consistent with the expectation that both source accessions contribute comparable genomic signals to the mixed-read fingerprint. This analysis demonstrates that Cam*K*-DB can recover dual-source signals from artificial mixed-read datasets when both contributing accessions are represented in the database.

However, this *in silico* assessment should be interpreted as mixed-sample or two-source signal detection rather than formal biological parentage or hybrid-origin inference. A physical mixture of reads from two accessions does not recapitulate the genomic structure of a true biological hybrid, because hybrids inherit recombined haplotypes generated through meiosis and segregation. Therefore, formal inference of parentage, hybrid origin, or genealogical ancestry would require validated hybrid material, segregating progeny, or inheritance-aware analytical models.

Concordance with phylogenetic distance and out-of-database query behavior

To assess whether CamK-DB similarity reflected biologically meaningful genetic relationships, we compared the similarity scores with phylogenetic distances obtained from the previously published Camellia phylogenetic tree of Kong et al. [12]. Most accessions showed negative correlations between CamK-DB similarity and phylogenetic distance, indicating that genetically closer accessions generally exhibited higher fingerprint similarity (Fig. 2D). These results support the biological relevance of CamK-DB similarity scores. We further assessed Cam*K*-DB behavior using an out-of-database query sample, SRR11672521, not being in the standardized TEA.db release. Although the exact accession was absent from the database, higher Cam*K*-DB similarity was associated with lower phylogenetic distance to database accessions ($r = -0.37$, $P = 4.79 \times 10^{-15}$; Fig. 2C). This result indicates that, when an exact database match is unavailable, CamK-DB can identify the closest available genomic relatives. Such results should be interpreted as nearest-neighbor relationships rather than definitive accession identification.

Sequencing-depth robustness and sketch-size sensitivity

Because MIKE-derived fingerprints depend on the subset of *k*-mer-derived elements recovered from sequencing reads, sequencing depth can influence the resulting fingerprint composition and Jaccard similarity estimates. We therefore evaluated the effect of coverage matching on fingerprint similarity. Coverage-comparison analysis showed that similarity was highest when query and database fingerprints were generated at matched genome coverage and decreased when coverage differed (Fig. 2A–B). This result supports the use of standardized 5× genome-coverage fingerprints for both database construction and query processing, thereby reducing technical bias caused by sequencing-depth heterogeneity.

We also evaluated the effect of sketch size on similarity-estimation accuracy and runtime. Sketch/pre_cnt values of 100, 500, 1000, 2000, 5000, 8000, and 10000 were tested by comparing sketched similarity estimates with full fingerprint comparisons. Small sketch sizes produced higher root mean square error (RMSE), whereas increasing the sketch size substantially reduced estimation error. The RMSE distribution reached a low and stable level around sketch/pre_cnt = 2000, while larger sketch sizes did not provide proportional improvement in accuracy. Runtime remained comparable among moderate sketch sizes but increased markedly at very large values. Together, these results support sketch/pre_cnt = 2000 as an appropriate balance between similarity-estimation accuracy and computational efficiency for standardized Cam*K*-DB queries

(Fig. S2A–B).

Group-level representation of the standardized database

Finally, we evaluated the distribution of accessions across *Camellia* groups in the standardized 418-accession database. The retained accessions were distributed across multiple taxonomic and genetic groups, with a median group size of 16 accessions (Fig. S2C). This indicates that the standardized Cam*K*-DB release preserves broad group-level representation for reference-free accession comparison. Nevertheless, some groups remain less densely represented than others, and future database expansions should prioritize additional sampling of underrepresented groups to improve resolution across the full diversity of *Camellia* germplasm.

Methods

Source resequencing datasets and licensing

Cam~~*K*-~~*K*-DB integrates whole-genome resequencing data from ~~735~~ 418 *Camellia* accessions. Newly generated data for 320 accessions were produced as part of a recent large-scale genomic survey of *Camellia* germplasm and are deposited in the GSA database (https://bigd.big.ac.cn/gsa/, BioProject, accession PRJCA026608 [12]). Previously published resequencing data for 415 accessions were downloaded from the GSA and NCBI SRA repositories under BioProjects PRJNA665594 [16] ~~and PRJCA011312 [17]~~, and in the GSA database under the accession PRJCA003090 [16], PRJNA646044 [18], PRJNA716079 [19], and PRJNA597714 [20]. All of these datasets were obtained from repositories that explicitly permit reuse for research purposes. In Cam~~*K*-~~*K*-DB, we only redistribute derived MinHash fingerprints, the SQLite database file, and sample-level metadata; we do not redistribute the original raw reads from third-party studies, thereby complying with the terms and conditions of the original data providers (Table S1, S5).

Sequencing data processing

For the construction of database, this study utilized a ~~large-scale~~ dataset of ~~735~~ 418 *Camellia* accessions, based on our recently published research on *Camellia's* evolutionary history. (DOI: 10.1038/s41588-025-02135-z) [21]. This publication encompassed an expanded dataset that included 802 newly sequenced reads and 523 previously sequenced reads. *Camellia* accessions selected in this work originated from 13 countries: Azerbaijan, China,

Georgia, ~~Iran,~~ Japan, Kenya, Laos, Myanmar, ~~Nepal, Russia~~, Sri Lanka, and the USA~~, and Vietnam~~.

-However, we used 320 newly sequenced accessions and ~~415~~ 98 publicly available accessions (total n = ~~735~~418) after QC for Cam~~K~~ *K*-DB. The dataset included diverse *Camellia* species: *C. oleifera*, *C. quinquelocularis*, *C. sasanqua*, *C. sinensis* var. *assamica*, *C. sinensis* var. *pubilimba*, *C. sinensis* var. *sinensis*, *C. tachangensis* var. *remotiserrata*, and *C. taliensis*. Importantly, the *C. sinensis* accessions represent a mix of wild, elite, and landrace varieties, whereas the remaining other species were included as wild samples. Among ~~735~~ 418 selected accessions, we used 320 newly sequenced *Camellia* accessions from multiple countries, including Azerbaijan, China, Georgia, Iran, Laos, Myanmar, Sri Lanka, and Russia, from our sequenced resource (originally sequencing depth ranging between 9-23)~~ (Table S1)~~. Genomic DNA from the leaves was extracted using the DNeasy Plant Mini Kit (Qiagen), followed by 5 µg to construct libraries with an insert size of 500 bp. Each sample consisted of 150 bp paired-end reads on the DNBSEQ-T7 platform (Beijing Genomics Institute). Furthermore, to increase the sample diversity, ~~415 deep~~ resequencing data~~ with more than 8× sequencing depth~~ were downloaded from public *Camellia* accessions (National Genomics Data Center, https://ngdc.cncb.ac.cn/) and (National Center for Biotechnology Information, https://www.ncbi.nlm.nih.gov/) from the given research [16–20]. Raw sequencing reads were down sampled to 5× subjected to quality control and removal of low-quality reads and adapter sequences by Trimmomatic with default parameters i.e., Trimmomatic PE -phred33 ILLUMINACLIP:adapters.fa:2:30:10 LEADING:3 TRAILING:3 SLIDINGWINDOW:4:15 MINLEN:50 [22]. The resulting high-quality reads were processed using the MIKE tool to generate minimum hash signatures for each sample (Table S1). Across the 418 datasets, the mean percentages of Q20 and Q30 bases were 97.62% and 92.56%, respectively, while the mean GC content was 39.71%. The Q20, Q30, and GC-content values ranged from 92.95% to 99.12%, 85.48% to 96.83%, and 37.99% to 45.89%, respectively (Tables S3 and S4). The number of retained reads per accession ranged from approximately 50.39 million to 102.14 million, with an average of 100.44 million reads. The corresponding sequence yield was 15 Gb on an average per accession.~~Quality assessment revealed high read quality, with Q20 and Q30 scores averaging 98.0% and 93.5%, respectively. The average GC content was approximately 40.1%, which was consistent with the expected GC content of *C. sinensis* genomes. The number of high-~~

~~quality reads per sample ranged from ~205 million to ~580 million, yielding a total base count per sample ranging from ~30 Gb to ~87 Gb.~~ Following adapter trimming and quality filtering, the high-quality reads were aligned to the chromosomal-scale *C. sinensis* TGY genome (~3.15 Gb) [23] by BWA [24] with default parameters. The alignment results demonstrated high sequence similarity and minimal ~~contamination.~~contamination. Genome coverage was ~~uniformly~~ high across all samples, exceeding 88% on average, with several samples achieving a genome anchorage greater than 94% (Table ~~S1~~S3, S4).

User guide and input data specifications

Cam*K*-DB provides both command-line and graphical interfaces for reference-free identification of *Camellia* accessions using MIKE-derived MinHash fingerprints. The current standardized release of Cam*K*-DB contains 418 *Camellia* accessions, each processed under the same 5× genome-coverage sampling strategy. This standardization is important because MIKE/Jaccard similarity can be affected by sequencing-depth differences between the database fingerprint and the query fingerprint. Therefore, query samples should be processed using the same 5× coverage-normalized workflow before comparison with the TEA.db database.

Input data

Cam*K*-DB accepts either raw sequencing files or precomputed MIKE fingerprint files. Supported input formats include FASTQ/FASTA files and MIKE-generated `.jac` files. The current release was validated primarily using short-read whole-genome resequencing data generated from Illumina-compatible or DNBSEQ paired-end platforms. For reliable comparison with the standardized TEA.db database, users should provide high-quality reads with sufficient usable depth to allow downsampling to 5× genome coverage. Query datasets with less than 5× usable coverage after quality filtering is not recommended for high-confidence identification.

Read preprocessing and coverage normalization

Before fingerprint generation, raw reads should be filtered to remove adapter contamination, low-quality bases, and short reads. In this study, reads were processed using quality-control steps equivalent to adapter trimming, low-quality base removal, sliding-window quality filtering, and exclusion of reads shorter than 50 bp. After quality filtering, query reads should be downsampled to 5× genome coverage using the same

genome-size estimate applied during database construction. This step is required because fingerprints generated at unmatched coverage levels may show reduced similarity even when they originate from the same accession.

MIKE fingerprint generation

After preprocessing and 5× downsampling, query reads are converted into MIKE `.jac` fingerprint files using the same parameters used for database construction. The *k*-mer length should be set to *k*=21, following the MIKE framework. The recommended sketch/pre_cnt value for Cam*K*-DB queries is 2000, based on sensitivity analysis showing a favorable balance between similarity-estimation accuracy and runtime. Query fingerprints should therefore be generated using *k*=21, 5× genome coverage, and sketch/pre_cnt=2000 to ensure consistency with the standardized TEA.db database.

Command-line query

The command-line interface is suitable for batch analysis and high-throughput screening. A typical query uses the TEA.db database, a query `.jac` file, and the recommended sketch/pre_cnt value:

`query_db TEA.db query_sample.jac 2000`

The program compares the query fingerprint against the standardized Cam*K*-DB database and returns the top-ranked accessions according to Jaccard similarity. The output table includes accession identifiers, names, taxonomic or genetic group information, geographic metadata, and similarity scores.

Graphical user interface

The graphical user interface provides an interactive workflow for users who prefer not to run command-line queries. Users first select the standardized TEA.db database and then provide either a precomputed `.jac` fingerprint or a processed query file for fingerprint generation. After running the query, the GUI displays the top-ranked database accessions with their metadata and similarity scores. Results can be sorted by similarity to identify the closest database matches Fig. S1.

Interpretation of query results

Cam*K*-DB should be interpreted as a nearest-neighbor genomic fingerprinting system.

A high top-ranked similarity score together with a clear separation between the first and second hits supports a confident exact or near-clonal match, provided that the accession is represented in the database. Moderate similarity may indicate a close relative or accession from the same genetic group. Low similarity or a small top1–top2 margin suggests that the query accession may be absent from the current database or poorly represented by available database accessions.

For samples whose exact identity or close relatives are not present in Cam*K*-DB, the top-ranked hits should not be interpreted as definitive identity assignments. Instead, they represent the closest available genomic neighbors within the standardized database. Users should therefore consider both the absolute similarity value and the top-hit margin when interpreting results.

Limitations

The current standardized Cam*K*-DB release contains 418 accessions processed under a matched 5× genome-coverage strategy. Query fingerprints generated at arbitrary or unmatched coverage should not be directly compared with the standardized database, because coverage differences can reduce Jaccard similarity. Formal biological parentage or hybrid-origin inference is not supported by the current release; mixed-read analyses should be interpreted only as dual-source or mixed-sample signal detection. Future releases may incorporate additional accessions and further optimize parameters for specific *Camellia* groups or sequencing platforms.

User guide

A dual-interface system comprising both a graphical user interface (GUI) and a command-line interface (CLI) was developed to enable flexible, efficient querying of the Cam*K*-DB database. CLI, accessible via the syntax /path/to/query_db Cam*K*-DBsample.jac [pre_cnt], complements GUI by enabling high-throughput scriptable database interactions. Here, Cam*K*-DB specifies the target database, sample.jac denotes the query fingerprint, and the optional [pre_cnt] parameter constrains the number of hash buckets compared, thereby reducing the computational overhead for preliminary analyses. Underlying both interfaces is the query_db module, which employs three core functions to optimize the similarity calculations. A max-heap-based algorithm dynamically retains the top 10 most identical samples during database scans, thus minimizing the sorting costs.

The GUI, built using the QT framework, provides an intuitive platform for users to query the database by name, taxonomic classification, geographic region, or sequencing data (in the FASTQ format) to identify unknown tea tree varieties and their closest genetic relatives. Query results are displayed in a sortable table, detailing key attributes, such as tea tree name (Chinese and English), species, taxonomic group, geographic origin, and similarity score. To streamline computational workflows, the GUI automates database detection, prompting users to locate or regenerate the db/Cam*K*-Db file if missing. Query execution begins by selecting an ".jac" fingerprint file via the "Select" button, followed by parallelized processing initiated by the "Query" button. During the analysis, user inputs are temporarily disabled, and a frameless model loading dialog provides visual feedback, ensuring a simplistic and user-friendly experience (Figure 2).

Supplementary information

Supplementary Figure S1. CamK-DB graphical user interface workflow.
Overview of the graphical workflow for selecting a query input, loading the standardized TEA.db database, processing or selecting a MIKE .jac fingerprint, performing the similarity search, and displaying the top-ranked accessions together with their identifiers, metadata, and Jaccard similarity values.

Supplementary Figure S2. Effects of sketch size on CamK-DB performance and group-level representation.
(A) Root mean square error between similarity estimates generated using different sketch/pre_cnt values and those obtained from the full fingerprint comparison. (B) Query runtime across the tested sketch/pre_cnt values. The combined accuracy and runtime analyses support sketch/pre_cnt = 2000 as the recommended setting for CamK-DB queries. (C) Distribution of the 418 standardized accessions among the 14 genetic groups represented in CamK-DB. Group sizes ranged from 1 to 106 accessions, with a median of 16, demonstrating broad but uneven representation across groups.

Supplementary Table S1. Metadata and sequencing information for the Camellia accessions included in CamK-DB.
List of the 418 accessions used for database construction, including accession

identifiers, names, taxonomic assignments, genetic-group classifications, geographic origins, sequencing-data sources, and associated database information.

Supplementary Table S2. Distribution of CamK-DB accessions among genetic groups and SQLite tables.
Summary of the 14 genetic groups represented in the standardized database, including the corresponding SQLite table names and the number and percentage of accessions assigned to each group.

Supplementary Table S3. Sequencing-read quality statistics for the 418 Camellia accessions.
Accession-level quality-control metrics, including the number of retained reads, total retained bases, Q20 and Q30 percentages, GC content, and estimated post-processing sequencing depth.

Supplementary Table S4. Reference-mapping and genome-coverage statistics for the 418 Camellia accessions.
Accession-level alignment statistics generated against the *C. sinensis* TGY reference genome, including mapping rate, mapped-read counts, genome-coverage breadth, and other relevant alignment-quality measurements.

Supplementary Table S5. List of Archive_Metadata, downloading links and relevant information

Supplementary data

Supplementary Table S1. List of all tea accessions, quality metrics, and their related information used in database construction.

Author Contributions

Conceptualization: X.Z., F.W. and B.Z.; Methodology and algorithm development: F.W. and Noor; Database and GUI development: S.Z.; Validation and analysis: W.K., Y.S. and H.F.; Writing: Noor and F.W: Author B; Writing-review & editing: F.W. and X.Z.

Data availability

The CamK-*K*-DB dataset, including the TEA.db SQLite database, per-sample MinHash fingerprint files and the main program, is archived on Zenodo (DOI:

10.5281/zenodo.15588935).

Newly generated raw whole-genome resequencing data are publicly available in the GSA database (https://bigd.big.ac.cn/gsa/) under BioProject accession PRJCA026608. Previously published resequencing data used to construct Cam~~K-~~K-DB can be accessed under BioProjects PRJNA665594, ~~PRJCA011312,~~ PRJCA003090, PRJNA646044, PRJNA716079 and PRJNA597714 in GSA and NCBI SRA.

Code Availability

All software and analytical scripts developed for this study are publicly available under open-source licenses via GitHub repositories:

-MIKE toolkit:

(https://github.com/Argonum-Clever2/mike)

Cam~~K-~~K-DB database and GUI:

(https://github.com/sc-zhang/CamK-DB)

Data access note

We have uploaded a database file "TEA.db" for Cam~~K-~~K-DB and the main program to zenodo, the doi is:

https://doi.org/10.5281/zenodo.15588935

Competing Interests

The authors declare no competing interests.

Funding Information

This work was supported by National Natural Science Foundation of China grant (No. 32222019 to X.Z.) and Shenzhen Science and Technology Program (Grant No. RCYX20210706092103024 to X.Z.), the Open Fund of Collaborative Innovation Center of Chinese Oolong Tea Industry (2025W 01), the Project of Science and Technology Plan of Nanping city (N2024Z007), the Project of Science and Technology Plan of Fujian Province (2023N5013, 2024N3013), the Scientific Research Fund of Wuyi University (2018N2004, 2021L3058), the Special Funds for Colleges and Universities in Fujian Province to Improve the Level of Running Schools (No.77 [2022]).

References

1. Zhang, Z. B. *et al.* Understanding the Origin and Evolution of Tea (*Camellia sinensis* [L.]): Genomic Advances in Tea. *J Mol Evol* 91, 156–168 (2023).
2. Shen, Y. *et al.* Development of Genome-Wide Intron Length Polymorphism (ILP) Markers in Tea Plant (*Camellia sinensis*) and Related Applications for Genetics Research. *Int J Mol Sci* 25, 3241 (2024).
3. Wang, X., Xin, B., Guo, M., Yu, G. & Wang, J. GWASTool: A web pipeline for detecting SNP-phenotype associations. *Fundamental Research* 4, 761–769 (2024).
4. Baird, N. A. *et al.* Rapid SNP Discovery and Genetic Mapping Using Sequenced RAD Markers. *PLoS One* 3, e3376 (2008).
5. Elshire, R. J. *et al.* A Robust, Simple Genotyping-by-Sequencing (GBS) Approach for High Diversity Species. *PLoS One* 6, e19379 (2011).
6. Boratyn, G. M. *et al.* BLAST: a more efficient report with usability improvements. *Nucleic Acids Res* 41, W29–W33 (2013).
7. Liu, S. & Koslicki, D. CMash: fast, multi-resolution estimation of k-mer-based Jaccard and containment indices. *Bioinformatics* 38, i28–i35 (2022).
8. Wang, F. *et al.* MIKE: an ultrafast, assembly-, and alignment-free approach for phylogenetic tree construction. *Bioinformatics* 40, (2024).
9. Lin, P. *et al.* The genome of oil-*Camellia* and population genomics analysis provide insights into seed oil domestication. *Genome Biol* 23, 1–21 (2022).
10. Zhang, L. *et al.* The tetraploid *Camellia oleifera* genome provides insights into evolution, agronomic traits, and genetic architecture of oil Camellia plants. *Cell Rep* 43, (2024).
11. Gong, W. *et al.* Chromosome-level genome of *Camellia* lanceoleosa provides a valuable resource for understanding genome evolution and self-incompatibility. *Plant Journal* 110, 881–898 (2022).
12. Kong, W. *et al.* Genomic analysis of 1,325 *Camellia* accessions sheds light on agronomic and metabolic traits for tea plant improvement. *Nat Genet* 57, 997–1007 (2025).
13. An, Y., Zhang, X., Jiang, S., Zhao, J. & Zhang, F. TeaPVs: a comprehensive genomic variation database for tea plant (*Camellia sinensis*). *BMC Plant Biol* 22, 1–7 (2022).
14. Chen, J. D. *et al.* TeaGVD: A comprehensive database of genomic variations for uncovering the genetic architecture of metabolic traits in tea plants. *Front Plant Sci* 13, (2022).
15. ~~Zentgraf, J. & Rahmann, S. Swiftly Identifying Strongly Unique k-Mers. in *Leibniz International Proceedings in Informatics, LIPIcs* vol. 312 (Schloss Dagstuhl- Leibniz-Zentrum fur Informatik GmbH, Dagstuhl Publishing, 2024).~~
16. Zhang, X. *et al.* Haplotype-resolved genome assembly provides insights into evolutionary history of the tea plant *Camellia sinensis*. *Nat Genet* 53, 1250–1259 (2021).
17. ~~Lei, Y. *et al.* Whole genome resequencing reveals the origin of tea in Lincang. *Front Plant Sci* 13, 984422 (2022).~~
18. Wang, X. *et al.* Population sequencing enhances understanding of tea plant evolution. *Nature Communications 2020 11:1* 11, 1–10 (2020).
19. Lu, L. *et al.* Genome-level diversification of eight ancient tea populations in the Guizhou and Yunnan regions identifies candidate genes for core agronomic traits. *Hortic Res* 8, 1–10 (2021).
20. Xia, E. *et al.* The Reference Genome of Tea Plant and Resequencing of 81 Diverse Accessions Provide Insights into Its Genome Evolution and Adaptation.

Mol Plant 13, 1013–1026 (2020).

21. Kong, W. *et al.* Genomic analysis of 1,325 *Camellia accessions* sheds light on agronomic and metabolic traits for tea plant improvement. *Nat Genet* https://doi.org/10.1038/s41588-025-02135-z (2025) doi:10.1038/s41588-025-02135-z.
22. Bolger, A. M., Lohse, M. & Usadel, B. Trimmomatic: a flexible trimmer for Illumina sequence data. *Bioinformatics* 30, 2114–2120 (2014).
23. Zhang, X. *et al.* Haplotype-resolved genome assembly provides insights into evolutionary history of the tea plant *Camellia sinensis*. *Nature Genetics 2021 53:8* 53, 1250–1259 (2021).
24. Li, H. & Durbin, R. Fast and accurate short read alignment with Burrows–Wheeler transform. *Bioinformatics* 25, 1754–1760 (2009).

Figure Captions

Figure 1. The relationships among all tables are organized around the central tea_info table, which stores metadata for every sample. For each group (e.g. species, region or other logical subset), an additional table was created with three fields: id, name and similarity. The id field is the primary key (PK) of the group table and simultaneously acts as a foreign key referencing the corresponding sample in tea_info. The similarity field stores the MinHash *k*-mer sketch produced by MIKE, which is used to compute Jaccard similarity between each stored sample and a query.

Figure 2. Regional information of the dataset and framework for the construction and implementation of the database. **Figure 1.** MIKE-based fingerprint generation and Jaccard similarity calculation.

Quality-filtered reads are downsampled to 5× genome coverage and decomposed into 21-mers. Identical 21-mers are deduplicated, and each 21-mer is divided into a 10-bp prefix and an 11-bp suffix. Similar prefix sharing *k*-mers are grouped, and MIKE records a minimized hash-derived value from the corresponding suffix group. The resulting (prefix, MinHash (suffix)) elements are stored as ".jac" fingerprints and compared using Jaccard similarity. Query samples are processed using the same k=21 and sketch/pre_cnt=2000 settings before comparison with TEA.db.

Figure 2. Validation of coverage standardization and biological relevance of CamK-DB similarity.

(A) Effect of matched and unmatched sequencing coverage on MIKE fingerprint similarity for accession CRR1173463. (B) Random-subsampling analysis showing the

effect of query subsampling level and database fingerprint coverage on similarity.
(C) Relationship between Cam*K*-DB similarity and phylogenetic distance for the out-of-database accession SRR11672521. Phylogenetic distances were obtained from the published Camellia phylogenetic tree reported by Kong et al. [12].
(D) Distribution of Pearson correlation coefficients between Cam*K*-DB similarity and published phylogenetic distances. Negative values indicate that higher fingerprint similarity generally corresponds to shorter phylogenetic distance.

Figure 3. Architecture of the standardized Cam*K*-DB database.
Cam*K*-DB contains 418 standardized *Camellia accessions* represented by MIKE-derived MinHash fingerprints generated at 5× genome coverage. The SQLite database includes a central tea_info metadata table linked to group-specific fingerprint tables. The fingerprint field stores encoded MIKE prefix-MinHash features used for Jaccard similarity calculation, rather than precomputed pairwise similarity values.

Figure ~~3~~S1. Cam*K*-DB graphical user interface (GUI) workflow for varietal identification (steps 1-6). Pictures are arranged from top-to-bottom. Step 1 - Choose query input. Select either a local FASTQ/FASTA file or an existing sample from the database, designated as the query. Step 2 automates database detection or prompts for creating “Tea.db” file and GUI interface provides a selection bar for the following step. Step 3- Open “.M~~m~~inH~~h~~ash.jac “file for processing. Step 5 - Run similarity search and inspect results. Launch the search to obtain nearest neighbors. Step 6 - The results table shows each candidate’s sample ID, name, and Jaccard similarity (higher is closer).

Figure S2. Effect of sketch size on Cam*K*-DB accuracy and runtime.
(A) RMSE between sketched similarity estimates and full fingerprint comparison across sketch sizes. RMSE decreased with increasing sketch size and stabilized around 2000. (B) Runtime across sketch sizes. Query time remained low at moderate sketch sizes but increased sharply at very large values. Together, these results support sketch/pre_cnt = 2000 as a suitable balance between similarity-estimation accuracy and computational efficiency. (C) . The database includes multiple taxonomic and genetic groups, with a median group size of 16 accessions, indicating higher genetic diversity. The open database-construction source codes also allow users to incorporate additional accessions and generate customized Cam*K*-DB databases for specific germplasm collections.

Table 1. Self-identification performance of standardized Cam*K*-DB for accessions represented in TEA.db. Ten accessions represented in the standardized database were

queried against TEA.db using the same 5× MIKE fingerprint-generation workflow. In all cases, the top-ranked hit corresponded to the same accession as the query sample.

Table1. Query result of samples contained in the database

Sample	*Primary match*		*Secondary match*	
	**ID**	**similarity**	**ID**	**similarity**
C5A	C5A	0.997897	C3A	0.338155
ZH-1A	ZH-1A	0.992727	ZH-4A	0.364367
SRR12322236	SRR12322236	0.968909	SRR11672538	0.605417
SRR12322243	SRR12322243	0.968369	SRR12265472	0.366153
SRR12333999	SRR12333999	0.965834	SRR12329554	0.35455
SRR12337253	SRR12337253	0.960978	SRR12322233	0.295263
SRR12337254	SRR12337254	0.96791	SRR12328812	0.401559

Table2. Query results for two-sample mixed resequencing data

Sample1	*Sample2*	*Primary match*		*Secondary match*	
		**ID**	**similarity**	**ID**	**similarity**
SRR12322236	**SRR12322243**	SRR12322243	0.578255	SRR12322236	0.553881
SRR12322236	**SRR12333999**	SRR12333999	0.526315	SRR12322236	0.488366
SRR12322236	**SRR12337253**	SRR12337253	0.537477	SRR12322236	0.492161
SRR12322236	**SRR12337254**	SRR12337254	0.549704	SRR12322236	0.513578
SRR12322243	**SRR12333999**	SRR12333999	0.516018	SRR12322243	0.505456
SRR12322243	**SRR12337253**	SRR12337253	0.531123	SRR12322243	0.506955
SRR12322243	**SRR12337254**	SRR12337254	0.538876	SRR12322243	0.525018
SRR12333999	**SRR12337253**	SRR12337253	0.509444	SRR12333999	0.501213
SRR12333999	**SRR12337254**	SRR12333999	0.511309	SRR12337254	0.508364
SRR12337253	**SRR12337254**	SRR12337253	0.518642	SRR12337254	0.507205

Sample	Primary match		Secondary match	
	**ID**	**Similarity**	**ID**	**Similarity**
**PT57B**	PT57B	0.573086	PT44B	0.275166
**SRR12265461**	SRR12265461	0.605142	K78A	0.301827
**SRR12265466**	SRR12265466	0.59301	K78A	0.311545
**Y12A**	Y12A	0.559994	K47A	0.318512
**Y28A**	Y28A	0.604648	Y15A	0.372634
**Y30A**	Y30A	0.603477	K78A	0.314028
**Y31A**	Y31A	0.579548	K47A	0.28647
**Y38A**	Y38A	0.607403	K78A	0.309742
**YN6A**	YN6A	0.543805	YN4A	0.33486
**ZH5A**	ZH5A	0.559406	K78A	0.289278

Table 2. Cam*K*-DB query results for artificial two-source mixed-read datasets. Artificial mixed-read datasets were generated by combining reads from pairs of accessions represented in the standardized Cam*K*-DB database. For all ten mixtures, the two highest-ranked database records corresponded to the two source accessions used to generate the query dataset. This result demonstrates that Cam*K*-DB can recover

dual-source signals from artificial mixed-read data when both source accessions are present in the database. This analysis should be interpreted as mixed-sample signal detection and not as formal biological parentage or hybrid-origin inference.

Sample1	***Sample2***	Primary match		Secondary match	
		**ID**	**Similarity**	**ID**	**Similarity**
**Y12A**	PT57B	Y12A	0.385688	PT57B	0.377445
**Y12A**	Y28A	Y28A	0.500619	Y12A	0.481872
**Y12A**	Y38A	Y38A	0.504148	Y12A	0.482033
**Y12A**	YN6A	Y12A	0.414585	YN6A	0.410418
**Y28A**	PT57B	Y28A	0.39659	PT57B	0.373197
**Y28A**	Y38A	Y38A	0.492228	Y28A	0.488699
**Y28A**	YN6A	Y28A	0.426494	YN6A	0.406801
**Y38A**	PT57B	Y38A	0.398635	PT57B	0.371728
**Y38A**	YN6A	Y38A	0.427172	YN6A	0.403255
**YN6A**	PT57B	YN6A	0.389391	PT57B	0.385629

Figure1

Click here to access/download;Figure;Figure-1.pdf

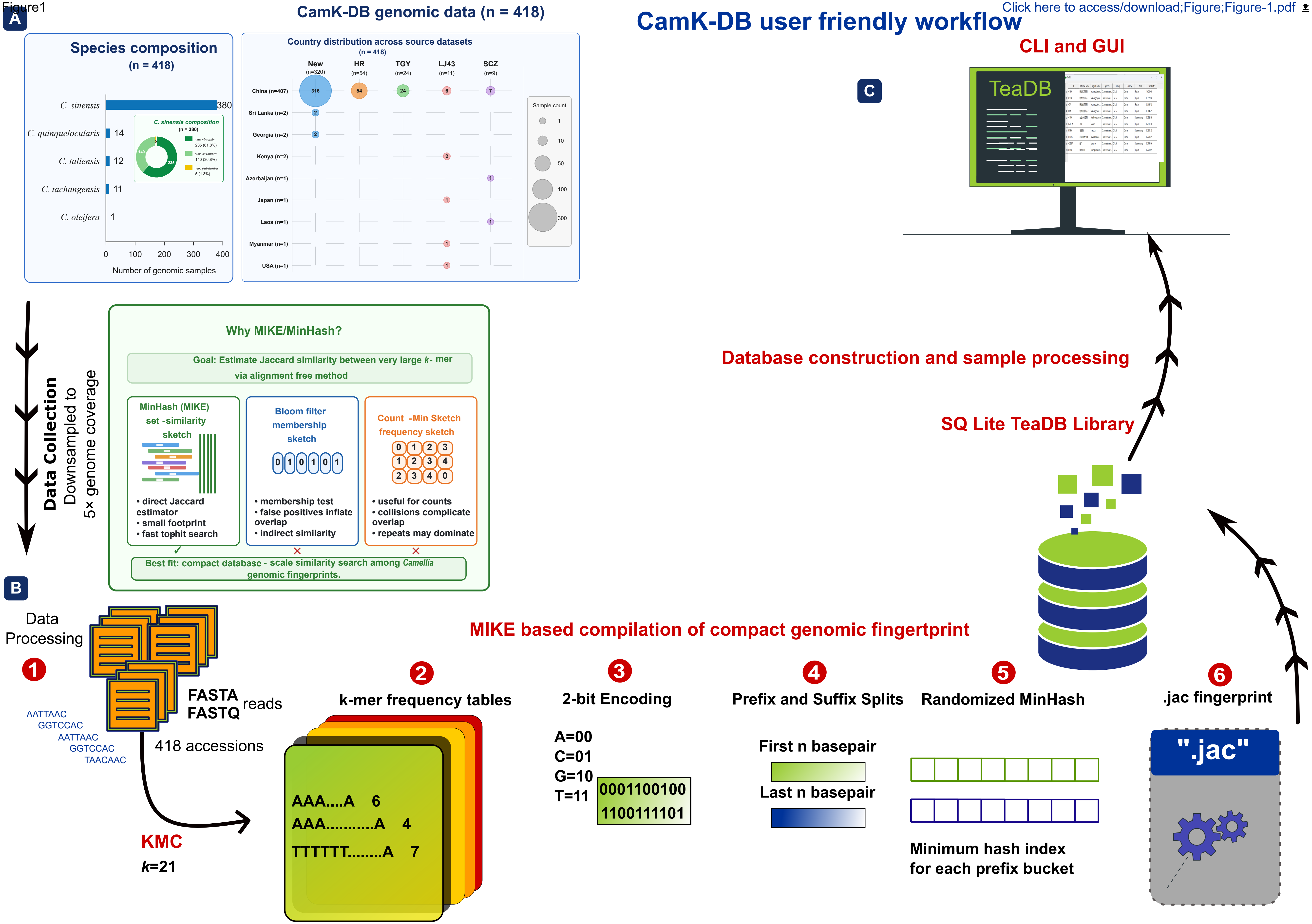

Figure2

Click here to access/download;Figure;Figure-2.pdf

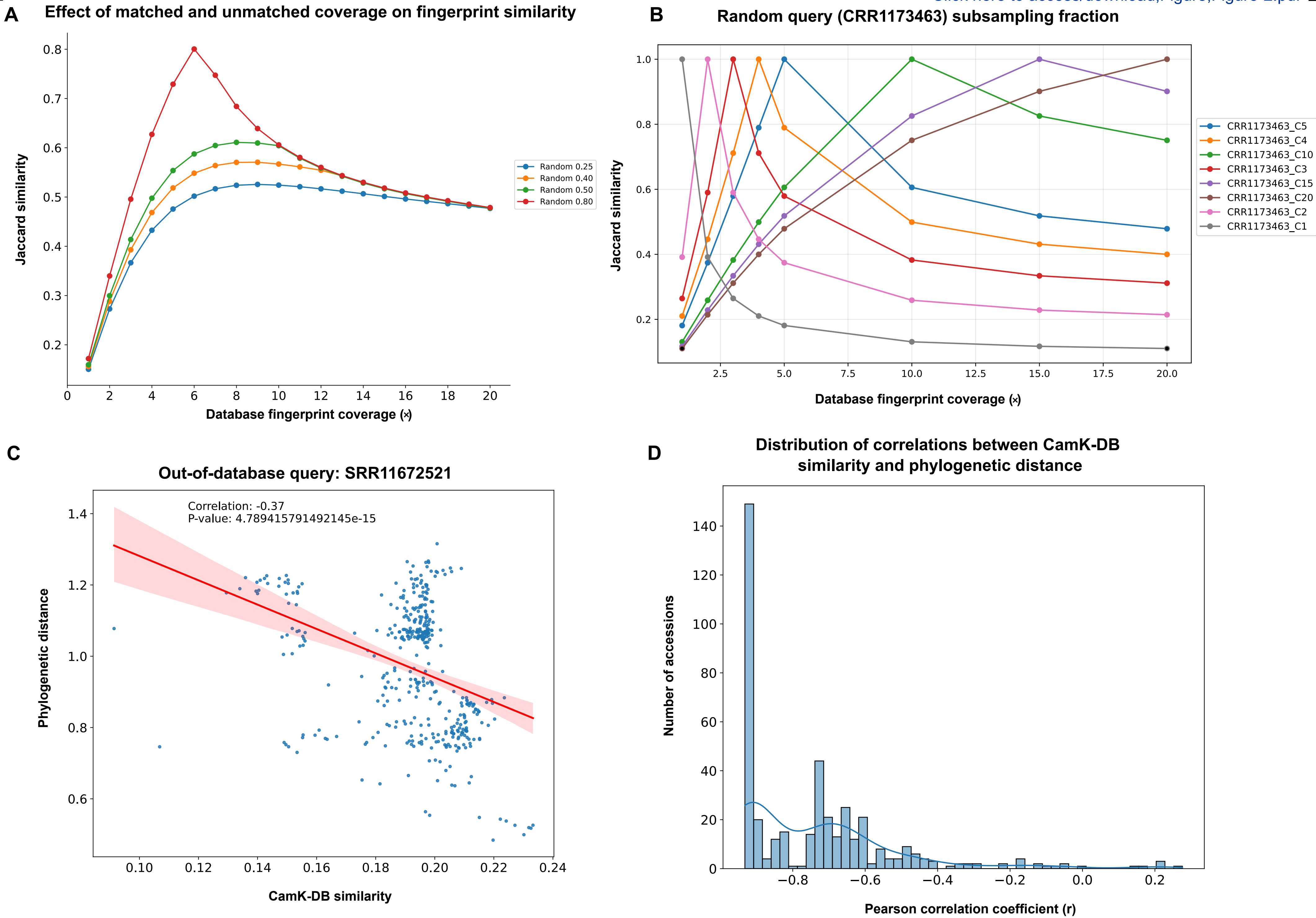

Figure3

Click here to access/download;Figure;Figure-3.pdf

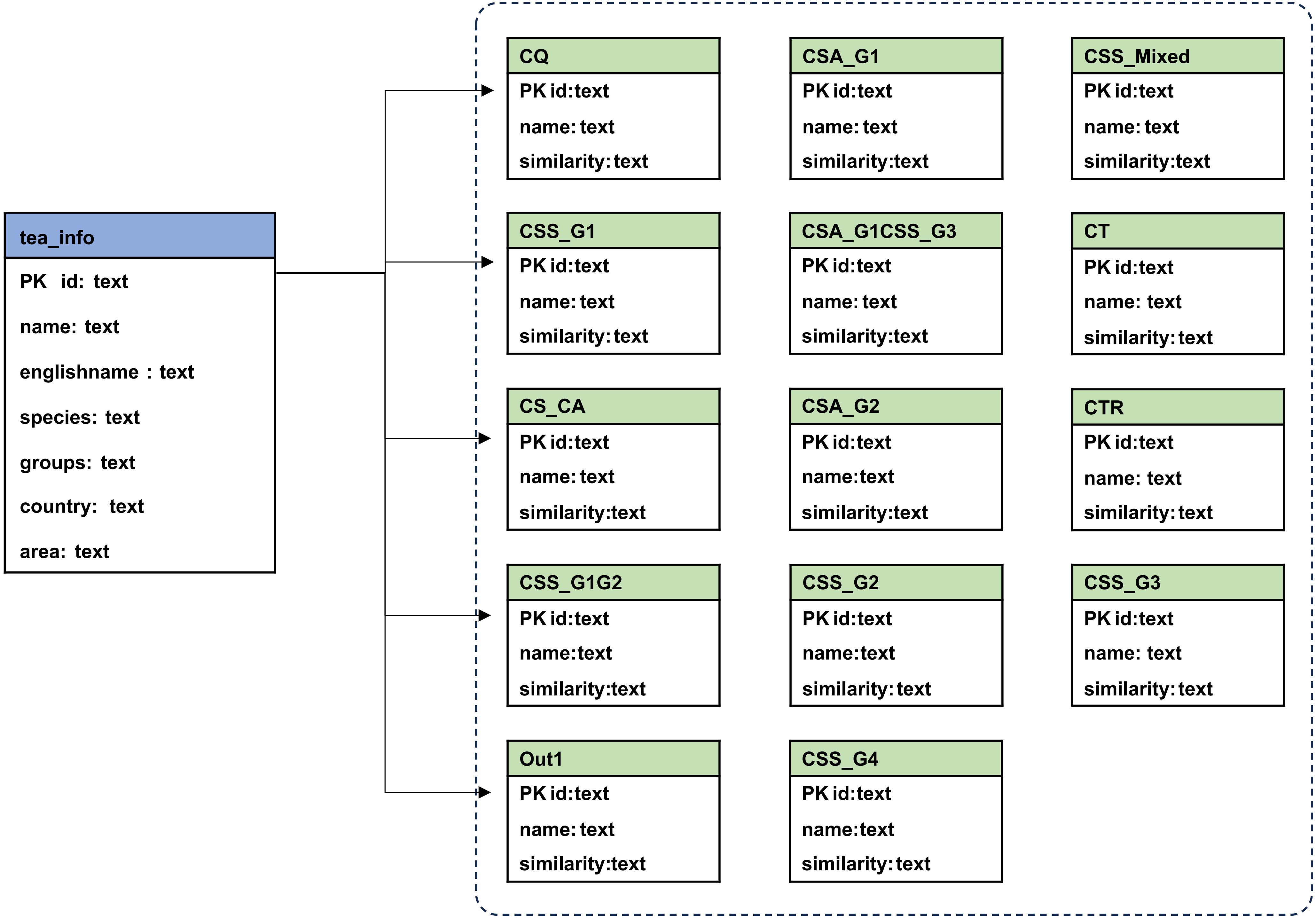

FigureS1

Click here to access/download;Figure;Figure-S1.pdf

SQLite based TeaDB framework

Step1

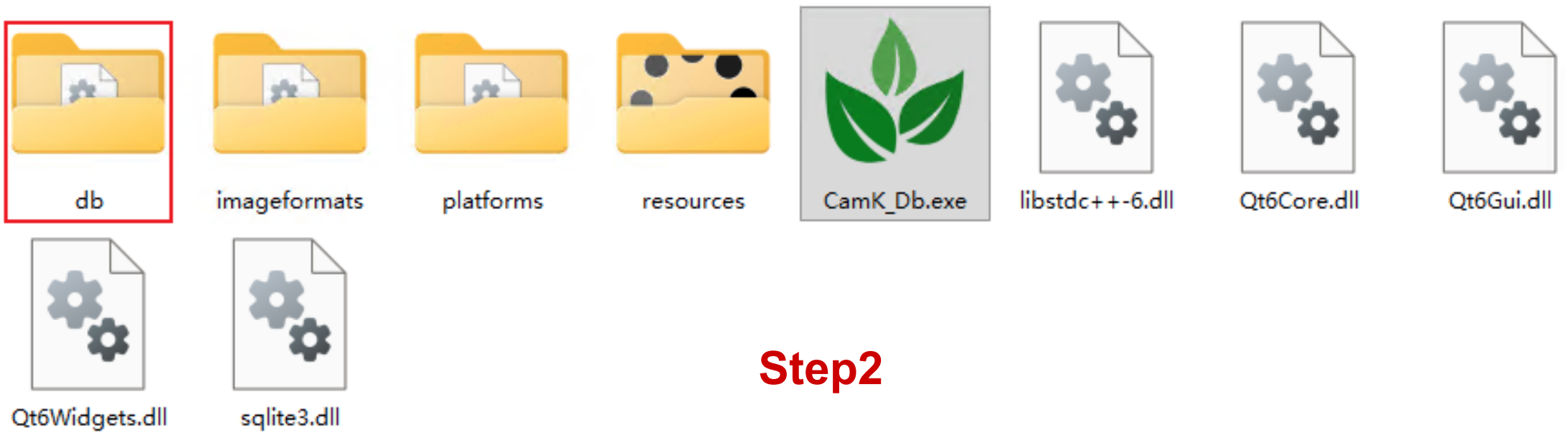

Step2

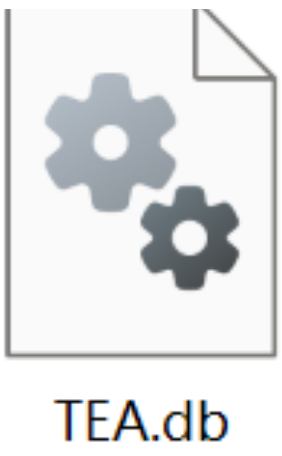

Step3

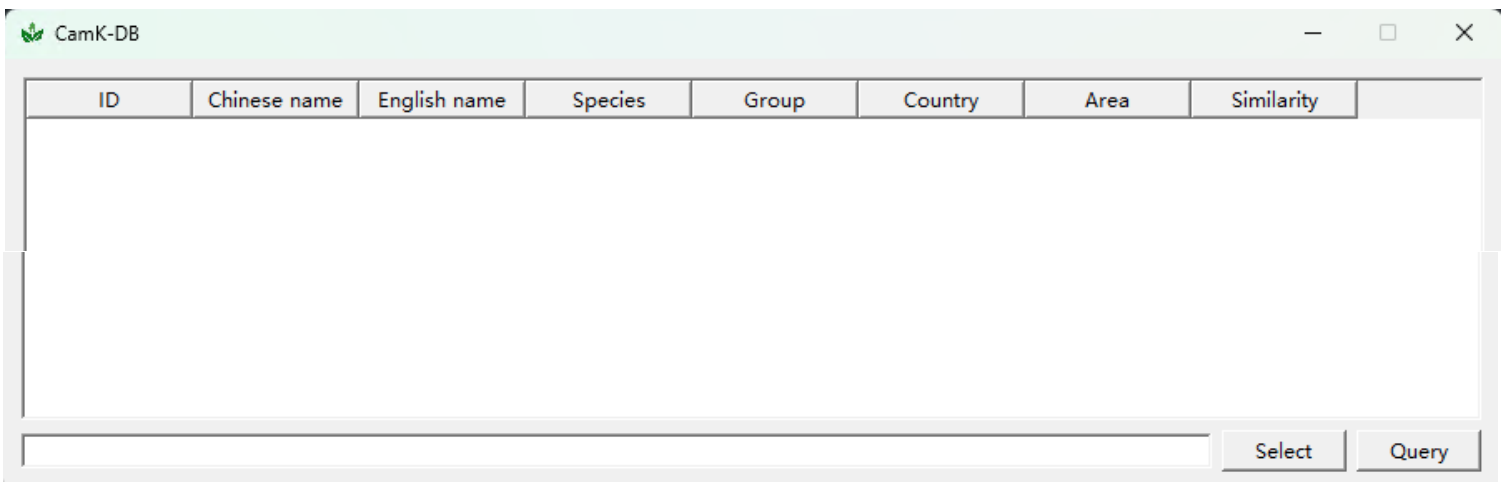

Step 4

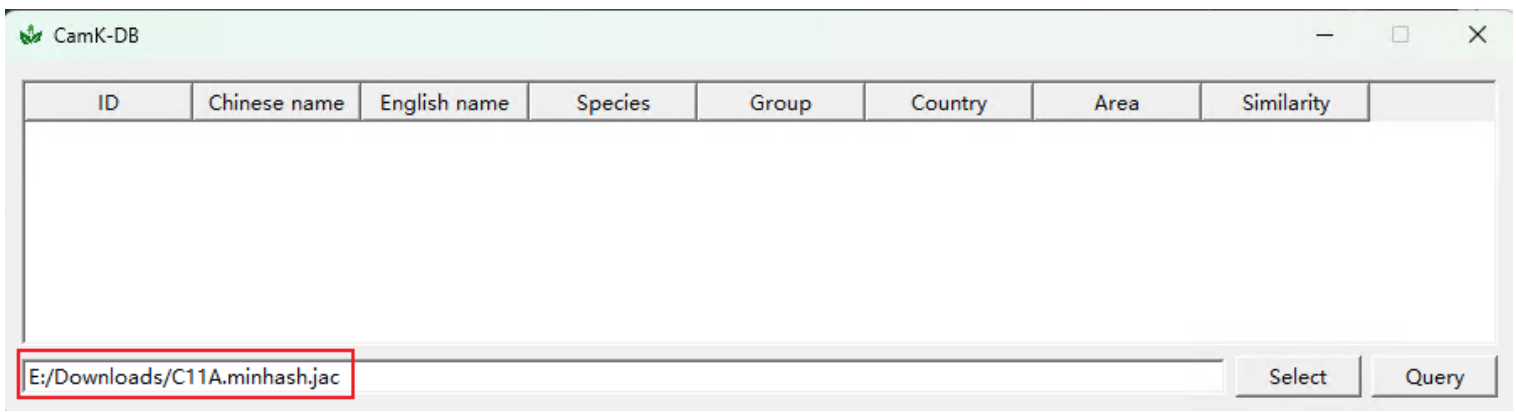

Step5

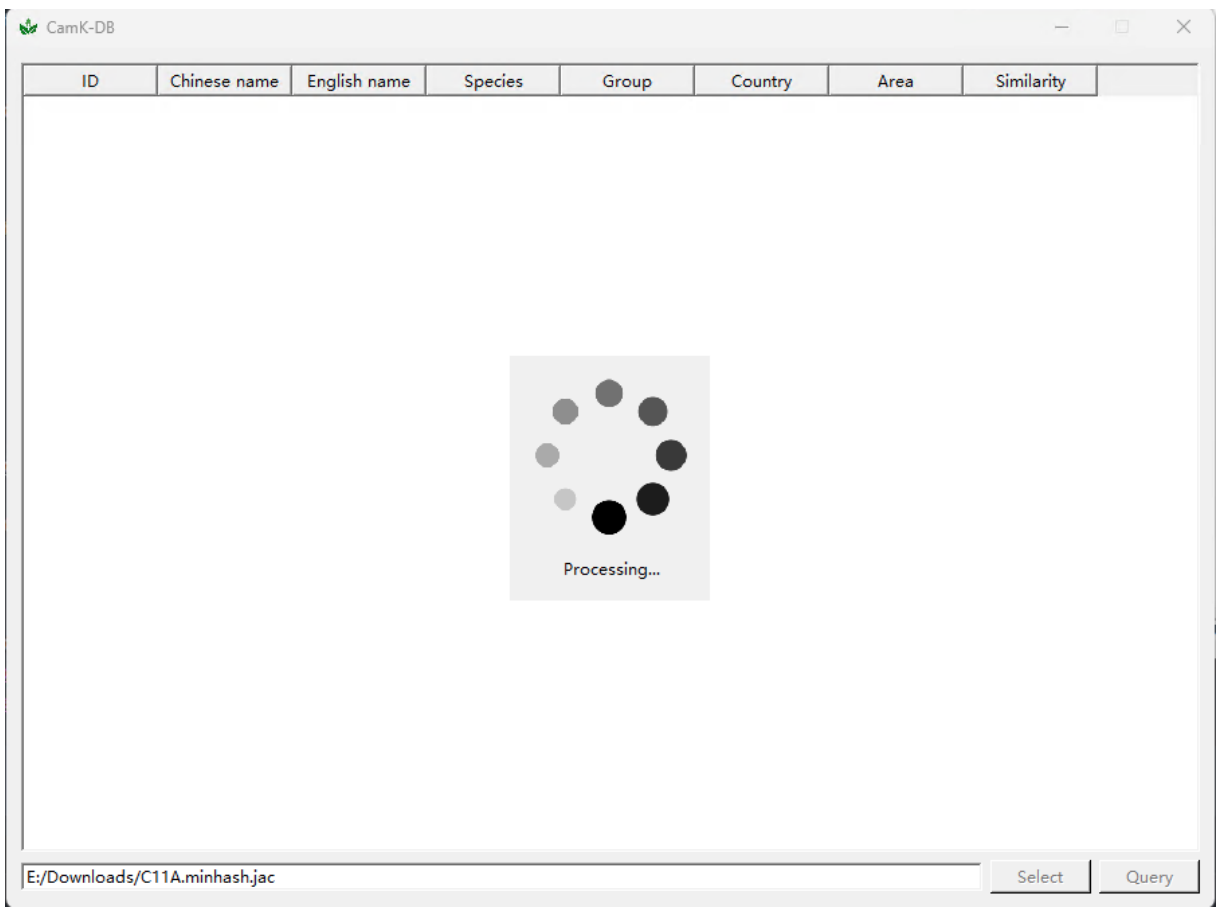

Step6

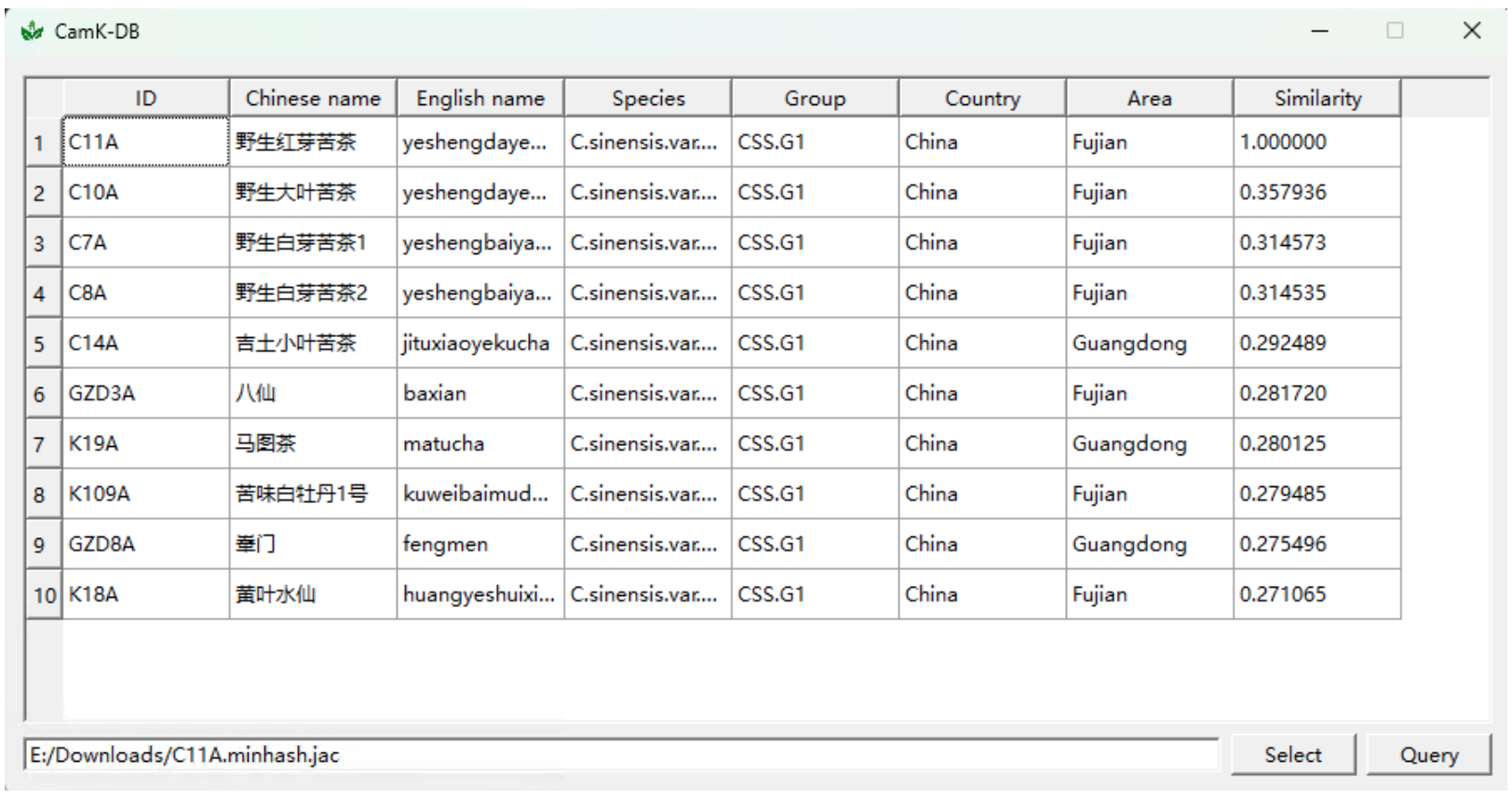

	ID	Chinese name	English name	Species	Group	Country	Area	Similarity
1	C11A	野生红芽苦茶	yeshengdaye...	C.sinensis.var....	CSS.G1	China	Fujian	1.000000
2	C10A	野生大叶苦茶	yeshengdaye...	C.sinensis.var....	CSS.G1	China	Fujian	0.357936
3	C7A	野生白芽苦茶1	yeshengbaiya...	C.sinensis.var....	CSS.G1	China	Fujian	0.314573
4	C8A	野生白芽苦茶2	yeshengbaiya...	C.sinensis.var....	CSS.G1	China	Fujian	0.314535
5	C14A	吉土小叶苦茶	jituxiaoyekucha	C.sinensis.var....	CSS.G1	China	Guangdong	0.292489
6	GZD3A	八仙	baxian	C.sinensis.var....	CSS.G1	China	Fujian	0.281720
7	K19A	马图茶	matucha	C.sinensis.var....	CSS.G1	China	Guangdong	0.280125
8	K109A	苦味白牡丹1号	kuweibaimud...	C.sinensis.var....	CSS.G1	China	Fujian	0.279485
9	GZD8A	峯门	fengmen	C.sinensis.var....	CSS.G1	China	Guangdong	0.275496
10	K18A	黄叶水仙	huangyeshuixi...	C.sinensis.var....	CSS.G1	China	Fujian	0.271065

FigureS2

Click here to access/download;Figure;Figure-S2.pdf

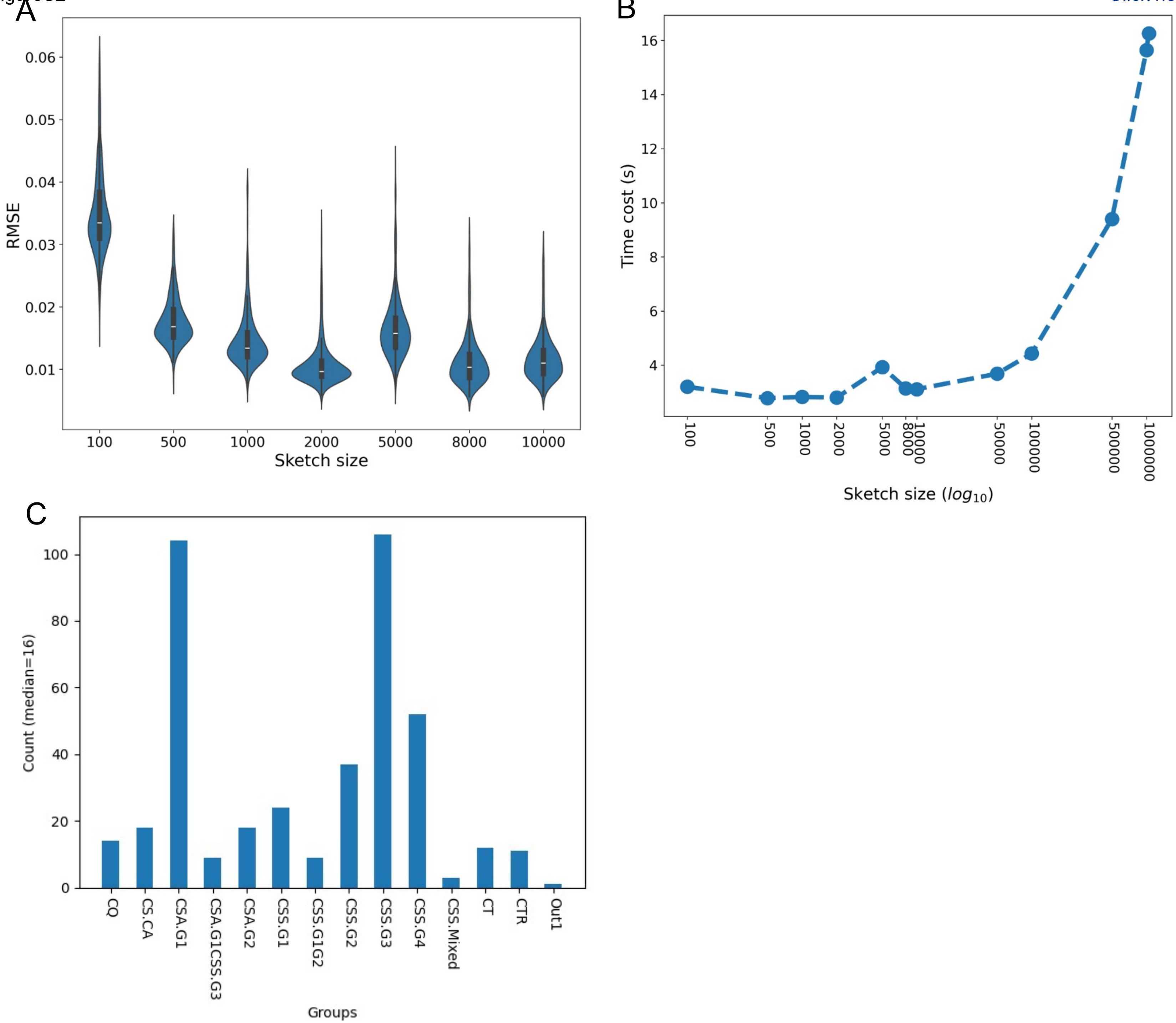

Click here to access/download
Supplementary Material
Response to Reviewers.docx

Supplementary file

Click here to access/download
Supplementary Material
Final-supplementry.xlsx

Click here to access/download
Supplementary Material
Cover-letter.pdf
