# Supplementary material for: CamK-DB: A k-mer MinHash fingerprint database for reference-free genotyping of Camellia accessions
Source: Gigascience. 2026 Aug 21;15:giag088. doi: 10.1093/gigascience/giag088 (PMC13548848; doi:10.1093/gigascience/giag088)
Supplement: Supplementary file 4 [file giag088_reviewer_1_report_original_submission.pdf]

Reviewer Report

Title: CamK-DB: A k-mer MinHash fingerprint database for reference-free genotyping of Camellia accessions

Version: Original Submission **Date:** 1/20/2026

Reviewer name: Reviewer 1

Reviewer Comments to Author:

This manuscript presents CamK-DB, a novel, alignment-free k-mer MinHash fingerprint database for reference-free genotyping of *Camellia sinensis* and related accessions. The work addresses a genuine need in tea genomics for a scalable, efficient tool for germplasm identification, phylogenetic inference, and breeding support. The database integrates a substantial dataset of 735 accessions, provides both CLI and GUI interfaces, and demonstrates promising validation results. The study is well-motivated, the methodology is clearly described, and the resource appears to be technically sound and publicly accessible. The manuscript is generally well-written and falls within the scope of the journal. However, several methodological and presentational aspects require clarification, expansion, or correction to strengthen the paper's rigor, reproducibility, and impact.

Major Comments:

1. Justification for Algorithm Selection (Lines 66-68):

The manuscript mentions several hashing-based techniques (MinHash, Bloom filter, Count-Min Sketch) but provides no rationale for selecting MinHash over others, particularly Bloom filters, which are also known for efficiency in handling large genomic datasets. Please clarify the comparative advantages of MinHash in this specific context (e.g., for similarity estimation via Jaccard index vs. membership queries) and whether a formal comparison was conducted to justify this choice as optimal for the stated objectives of rapid, accurate germplasm identification and comparison.

2. Choice of k-mer Size (k=21) (Lines 98-100):

The selection of k=21 is stated as the default/recommended setting in MIKE but is not biologically or computationally justified within this study. Please provide the reasoning behind this specific value. Were other k-mer lengths evaluated (e.g., 15, 31, 51)? If so, please present a brief analysis (e.g., in supplementary material) showing how different k values affect discrimination power, runtime, or database size. If not, please acknowledge this as a limitation or standard parameter from prior work and discuss its suitability for tea genomes.

3. Use of Unique k-mers and Data Heterogeneity (Lines 100-103; 225-234):

The manuscript states that unique k-mers are used to build MinHash fingerprints. Given the noted variability in sequencing depth, data volume, mapping rate, and genome coverage among the 735 accessions (Table S1), it is crucial to address:

ï¬ What is the approximate count of unique k-mers per sample, and how does it vary?

ï¬ How does variability in sequencing depth and data quality impact the number and stability of unique k-mers extracted?

ï¬ What normalization or standardization steps (if any) were applied to mitigate biases introduced by

such technical heterogeneity before sketch generation and similarity calculation?
4. Jaccard Similarity Calculation and Its Robustness (Lines 107-115):
The method for calculating Jaccard similarity based on shared unique k-mers requires further elaboration:
ï¬ How is a "shared unique k-mer" defined? Does it require an exact sequence match (presumably yes for k-mers), or are mismatches allowed?
ï¬ Are the sets S1 and S2 (the unique k-mer sets from two samples) required to be of comparable size? If two samples have vastly different numbers of unique k-mers (e.g., due to different coverage), how does this affect the Jaccard index? Have the authors evaluated the sensitivity of the similarity score to such disparities?
Please describe any preprocessing, filtering, or scaling procedures implemented to ensure fair comparisons between samples with differing k-mer set sizes.
5. Rationale for Sketch Size (1000) (Lines 118-120):
The use of a "1000-sketch size" is mentioned without justification. This parameter controls the resolution and accuracy of the MinHash approximation.
ï¬ What was the basis for choosing a sketch size of 1000? Was a sensitivity analysis performed with different sketch sizes (e.g., 500, 2000, 5000)?
ï¬ Please provide evidence or references showing that a size of 1000 provides an optimal balance between accuracy, storage, and computational speed for this specific dataset and application.
6. Clarity on Input Requirements and Tool Robustness (User Guide Section):
The user guide effectively describes how to use CamK-DB but lacks critical specifications on what data is suitable as input.
Minimum Requirements: Please explicitly state the required sequencing platform (or compatibility), recommended minimum sequencing depth, and necessary read length for reliable analysis.
Data Quality Thresholds: Specify the tool's tolerances or requirements for input data quality (e.g., Q-score thresholds, adapter contamination levels). What QC steps are mandatory before running MIKE to generate the ".jac" file?
Performance under Variable Depth: How do sequencing depth and coverage influence runtime and, more importantly, the accuracy of identification (e.g., top-hit confidence, similarity score stability)? A brief analysis or statement on the tool's robustness to suboptimal data would greatly enhance its practical utility guide.
Minor Comments:
1. Dataset Representativeness and Selection Criteria (Line 188):
Please state the average sequencing depth for the entire set of 735 accessions. More importantly, justify the selection of these specific 735 accessions. Do they comprehensively represent the genetic, geographical, and phenotypic diversity of cultivated and wild *Camellia*? A brief discussion on the representativeness and potential biases of the dataset would be valuable.
2. Terminology Accuracy (Lines 217-220):
The phrase "deep resequencing data with more than 8Ã— sequencing depth" is misleading. In current genomics standards, "deep" resequencing typically implies coverage â‰¥30x. Please rephrase to "resequencing data with a minimum depth of 8Ã—" or similar to avoid overstatement.
3. Completeness of Table S1:

Table S1 appears to contain detailed metrics only for newly sequenced data, leaving fields blank for publicly sourced accessions. To ensure transparency and reproducibility, please supplement the table with available metrics (e.g., SRA accession-linked depth, read counts if obtainable) for all 735 accessions or clearly note the source of each datum.
4. Expanded User Guide:
As noted in Major Comment 6, the User Guide section should be expanded to include a dedicated subsection on "Input Data Specifications" detailing platform, depth, quality control prerequisites, and file format requirements.
5. Validity for Samples Outside the Database (Technical Validation):
The validation convincingly shows performance for samples and mixtures within the database. Please comment briefly on the expected behavior or interpretive value when querying a sample whose true parents or close relatives are *not* in CamK-DB. How should users interpret the top hits in such scenarios?
6. Abbreviation Formatting (Lines 58-59):
The first instance of an abbreviation (e.g., likely "AFLPs" or similar from context) should be spelled out in full.
7. Formatting Consistency:
Line 103: "k-mers" - the 'k' should be italicized (k-mers).
Line 107: "MinHash" - ensure consistent formatting; it should not be italicized if it is not standard elsewhere.
Line 115: "Sâ‚‚" should use subscript for the numeral. Add a space: "sample 2".
8. Figure Reference (Lines 110-112):
The text referencing "Figure 1" describes a mathematical formula, but the current Figure 1 appears to depict database schema. Please verify all figure citations and ensure they accurately correspond to the described content. The formula for Jaccard similarity might be better placed in the main text or a dedicated methodology figure.

Methods

Are the methods appropriate to the aims of the study, are they well described, and are necessary controls included? Choose an item.

Conclusions

Are the conclusions adequately supported by the data shown? Choose an item.

Reporting Standards

Does the manuscript adhere to the journal's guidelines on [minimum standards of reporting?]() Choose an item.

Choose an item.

Statistics

Are you able to assess all statistics in the manuscript, including the appropriateness of statistical tests used? Choose an item.

Quality of Written English

Please indicate the quality of language in the manuscript: Choose an item.

Declaration of Competing Interests

Please complete a declaration of competing interests, considering the following questions:

- Have you in the past five years received reimbursements, fees, funding, or salary from an organisation that may in any way gain or lose financially from the publication of this manuscript, either now or in the future?
- Do you hold any stocks or shares in an organisation that may in any way gain or lose financially from the publication of this manuscript, either now or in the future?
- Do you hold or are you currently applying for any patents relating to the content of the manuscript?
- Have you received reimbursements, fees, funding, or salary from an organization that holds or has applied for patents relating to the content of the manuscript?
- Do you have any other financial competing interests?
- Do you have any non-financial competing interests in relation to this paper?

If you can answer no to all of the above, write 'I declare that I have no competing interests' below. If your reply is yes to any, please give details below.

I declare that I have no competing interests.

I agree to the open peer review policy of the journal. I understand that my name will be included on my report to the authors and, if the manuscript is accepted for publication, my named report including any attachments I upload will be posted on the website along with the authors' responses. I agree for my report to be made available under an Open Access Creative Commons CC-BY license (http://creativecommons.org/licenses/by/4.0/). I understand that any comments which I do not wish to be included in my named report can be included as confidential comments to the editors, which will not be published.

Choose an item.

To further support our reviewers, we have joined with Publons, where you can gain additional credit to further highlight your hard work (see: https://publons.com/journal/530/gigascience). On publication of this paper, your review will be automatically added to Publons, you can then choose whether or not to claim your Publons credit. I understand this statement.

Yes Choose an item.
