# Supplementary material for: CamK-DB: A k-mer MinHash fingerprint database for reference-free genotyping of Camellia accessions
Source: Gigascience. 2026 Aug 21;15:giag088. doi: 10.1093/gigascience/giag088 (PMC13548848; doi:10.1093/gigascience/giag088)
Supplement: Supplementary file 6 [file giag088_reviewer_2_report_original_submission.pdf]

Reviewer Report

Title: CamK-DB: A k-mer MinHash fingerprint database for reference-free genotyping of Camellia accessions

Version: Original Submission **Date:** 1/27/2026

Reviewer name: Reviewer 2

Reviewer Comments to Author:

This manuscript introduces CamK-DB, a database and software suite designed for the rapid genetic identification of tea (Camellia sinensis) germplasm. By leveraging the MinHash-based k-mer algorithm (MIKE), the authors integrated whole-genome resequencing data from 735 accessions to construct a lightweight genomic fingerprinting repository. The provision of both a Graphical User Interface (GUI) and a Command-Line Interface (CLI) facilitates accessible varietal identification.

While the resource is potentially valuable for the tea breeding community, several critical areas regarding the theoretical justification of parameters, experimental rigor, and data structural clarity require further address.

Major Comments

1. Justification of Algorithmic Parameters.

The manuscript utilizes the MIKE algorithm with default parameters (k=21 and sketch size=1000) but falls short of providing a sufficient theoretical or empirical basis for these choices. Given that k-mer-based analyses are highly sensitive to parameter selectionâ€”particularly in the context of the tea genome, which is characterized by high heterozygosity and a high proportion of repetitive elementsâ€—the mere adoption of default settings is inadequate. The authors should elaborate on the underlying algorithmic mechanics, such as the specific hash functions employed, and discuss how the selected k-mer size balances the trade-off between specificity and the noise introduced by sequencing errors or genomic complexity.

2. Rigor of Validation Strategy.

The technical validation presented in the study is currently insufficient as it relies heavily on "self-identification," where accessions used to construct the database are simply re-queried against it. This circular approach essentially confirms the database's internal consistency but fails to demonstrate the tool's robustness and generalizability when faced with independent, external datasets. To truly validate the utility of CamK-DB in a real-world setting, the authors should perform cross-validation or, ideally, test the system with newly sequenced samples that were strictly excluded from the database construction process to prove it can accurately identify samples it has not "seen" before.

3. Limitations in Parentage Inference Methodology.

The assessment of parentage inference is methodologically limited by the use of in silico mixed reads to simulate hybridization. While this approach effectively demonstrates the tool's ability to deconvolve mixed genomic signals from two different sources, it does not accurately reflect true biological hybridization. Biological hybrids undergo meiotic recombination and segregation, resulting in a unique k-mer composition that differs significantly from a simple linear physical mixture of parental reads. The

authors must clarify this distinction in the text and explicitly acknowledge that their current simulation primarily tests the detection of mixed samples rather than the inference of complex genealogical ancestry or hybrid origins.

Minor Comments

4. Database Architecture Clarity (Figure 1).

The description of the database architecture in Figure 1 requires significantly more detail to be transparent and reproducible. Specifically, the manuscript does not adequately explain the rationale behind partitioning the database into 13 distinct grouping tables, nor does it define the cryptic abbreviations used for these sub-tables. A clear mapping key or a more descriptive legend is necessary to help readers understand the logical organization and taxonomy of the stored data.

5. Quality of Visualizations (Figure 2).

Figure 2 is currently too schematic and lacks the necessary technical depth to fully illustrate the study's workflow. It fails to capture critical details of the MIKE algorithm, such as the hashing process or sketch generation, and the visual presentation is somewhat rudimentary for a high-impact publication. The authors should refine this figure to provide a more comprehensive and professionally polished representation of the computational pipeline that visually communicates the complexity of the methods used.

Methods

Are the methods appropriate to the aims of the study, are they well described, and are necessary controls included? Choose an item.

Conclusions

Are the conclusions adequately supported by the data shown? Choose an item.

Reporting Standards

Does the manuscript adhere to the journal's guidelines on minimum standards of reporting? Choose an item.

Choose an item.

Statistics

Are you able to assess all statistics in the manuscript, including the appropriateness of statistical tests used? Choose an item.

Quality of Written English

Please indicate the quality of language in the manuscript: Choose an item.

Declaration of Competing Interests

Please complete a declaration of competing interests, considering the following questions:

- Have you in the past five years received reimbursements, fees, funding, or salary from an organisation that may in any way gain or lose financially from the publication of this manuscript, either now or in the future?
- Do you hold any stocks or shares in an organisation that may in any way gain or lose financially from the publication of this manuscript, either now or in the future?
- Do you hold or are you currently applying for any patents relating to the content of the manuscript?
- Have you received reimbursements, fees, funding, or salary from an organization that holds or has applied for patents relating to the content of the manuscript?
- Do you have any other financial competing interests?
- Do you have any non-financial competing interests in relation to this paper?

If you can answer no to all of the above, write 'I declare that I have no competing interests' below. If your reply is yes to any, please give details below.

I declare that I have no competing interests

I agree to the open peer review policy of the journal. I understand that my name will be included on my report to the authors and, if the manuscript is accepted for publication, my named report including any attachments I upload will be posted on the website along with the authors' responses. I agree for my report to be made available under an Open Access Creative Commons CC-BY license (http://creativecommons.org/licenses/by/4.0/). I understand that any comments which I do not wish to be included in my named report can be included as confidential comments to the editors, which will not be published.

Choose an item.

To further support our reviewers, we have joined with Publons, where you can gain additional credit to further highlight your hard work (see: https://publons.com/journal/530/gigascience). On publication of this paper, your review will be automatically added to Publons, you can then choose whether or not to claim your Publons credit. I understand this statement.

Yes Choose an item.
