# Supplementary material for: CamK-DB: A k-mer MinHash fingerprint database for reference-free genotyping of Camellia accessions
Source: Gigascience. 2026 Aug 21;15:giag088. doi: 10.1093/gigascience/giag088 (PMC13548848; doi:10.1093/gigascience/giag088)
Supplement: Supplementary file 8 [file giag088_supplemental_files.zip › Figure-S1.pdf]

SQLite based TeaDB framework

Step1

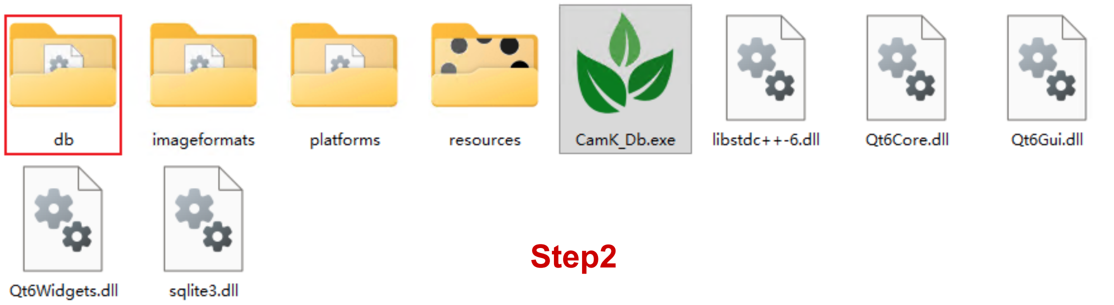

Step2

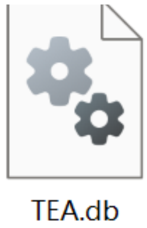

Step3

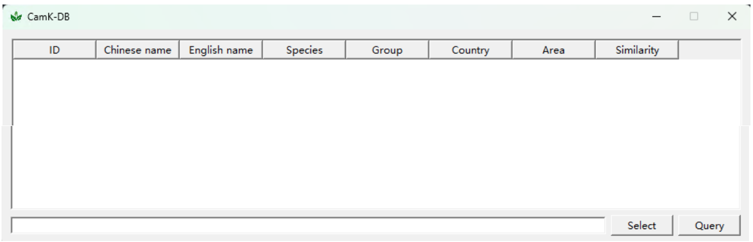

Step 4

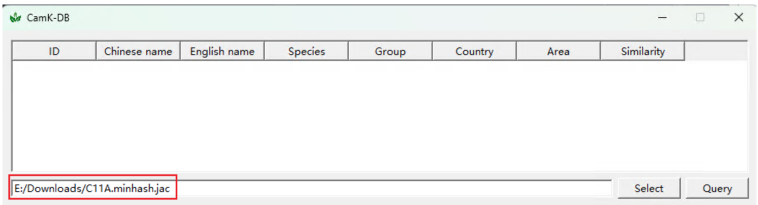

Step5

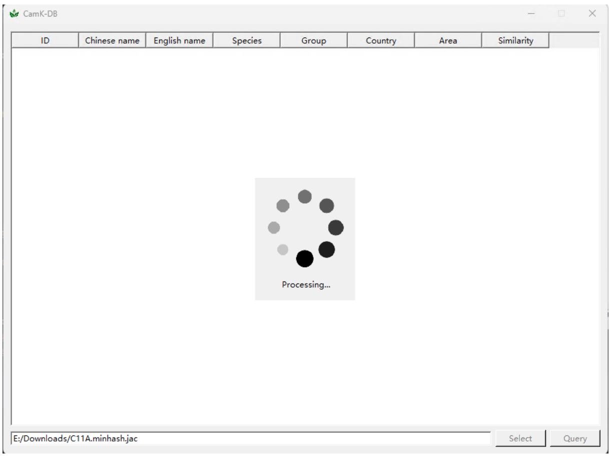

Step6

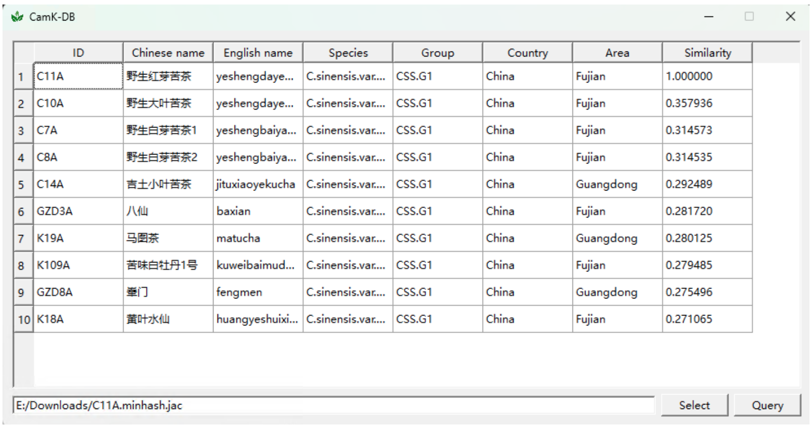

	ID	Chinese name	English name	Species	Group	Country	Area	Similarity
1	C11A	野生红芽苦茶	yeshengdaye...	C.sinensis.var....	CSS.G1	China	Fujian	1.000000
2	C10A	野生大叶苦茶	yeshengdaye...	C.sinensis.var....	CSS.G1	China	Fujian	0.357936
3	C7A	野生白芽苦茶1	yeshengbaiya...	C.sinensis.var....	CSS.G1	China	Fujian	0.314573
4	C8A	野生白芽苦茶2	yeshengbaiya...	C.sinensis.var....	CSS.G1	China	Fujian	0.314535
5	C14A	吉土小叶苦茶	jituxiaoyekucha	C.sinensis.var....	CSS.G1	China	Guangdong	0.292489
6	GZD3A	八仙	baxian	C.sinensis.var....	CSS.G1	China	Fujian	0.281720
7	K19A	马图茶	matucha	C.sinensis.var....	CSS.G1	China	Guangdong	0.280125
8	K109A	苦味白牡丹1号	kuweibaimud...	C.sinensis.var....	CSS.G1	China	Fujian	0.279485
9	GZD8A	峯门	fengmen	C.sinensis.var....	CSS.G1	China	Guangdong	0.275496
10	K18A	黄叶水仙	huangyeshuixi...	C.sinensis.var....	CSS.G1	China	Fujian	0.271065
