# Supplementary material for: CamK-DB: A k-mer MinHash fingerprint database for reference-free genotyping of Camellia accessions
Source: Gigascience. 2026 Aug 21;15:giag088. doi: 10.1093/gigascience/giag088 (PMC13548848; doi:10.1093/gigascience/giag088)
Supplement: Supplementary file 8 [file giag088_supplemental_files.zip › Figure-S2.pdf]

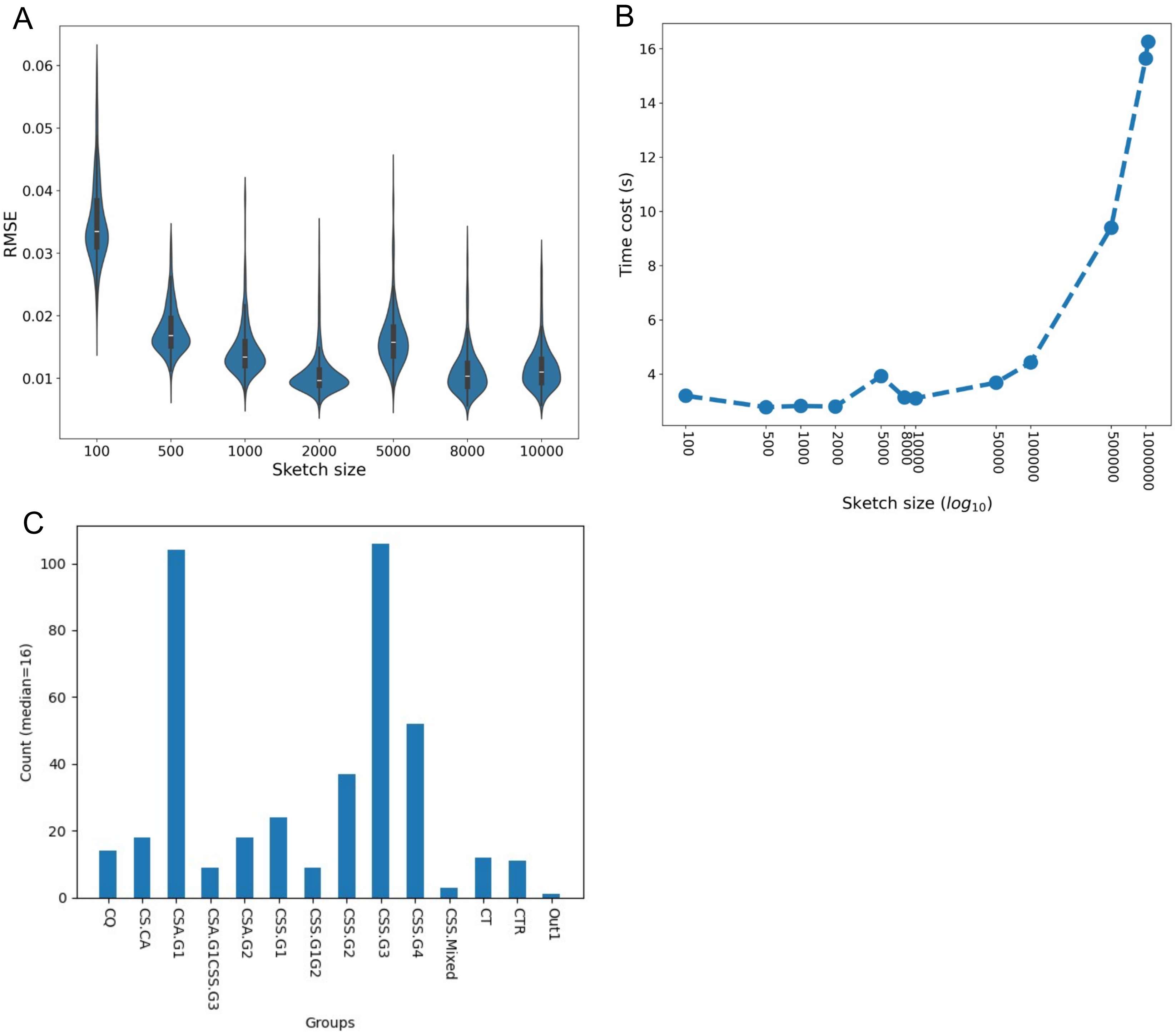
A
RMSE
0.01
0.02
0.03
0.04
0.05
0.06
100
500
1000
2000
5000
8000
10000
Sketch size
B
Time cost (s)
4
6
8
10
12
14
16
100
500
1000
2000
5000
8000
10000
50000
100000
500000
1000000
Sketch size (log_{10})
C
Count (median=16)
0
20
40
60
80
100
CQ
CS.CA
CSA.G1
CSA.G1CSS.G3
CSA.G2
CSS.G1
CSS.G1G2
CSS.G2
CSS.G3
CSS.G4
CSS.Mixed
CT
CTR
Out1
Groups
